# IntelliCage: the development and perspectives of a mouse- and user-friendly automated behavioral test system

**DOI:** 10.3389/fnbeh.2023.1270538

**Published:** 2024-01-03

**Authors:** Hans-Peter Lipp, Sven Krackow, Emir Turkes, Seico Benner, Toshihiro Endo, Holger Russig

**Affiliations:** ^1^Faculty of Medicine, Institute of Evolutionary Medicine, University of Zürich, Zürich, Switzerland; ^2^Institute of Pathology and Molecular Pathology, University Hospital Zürich, Zürich, Switzerland; ^3^Queen Square Institute of Neurology, University College London, London, United Kingdom; ^4^Center for Health and Environmental Risk Research, National Institute for Environmental Studies, Ibaraki, Japan; ^5^Phenovance, Chiba, Japan; ^6^TSE-Systems International, Berlin, Germany

**Keywords:** home-cage testing, animal welfare, automated behavioral analysis, standardization, ethology and ecology, comparative and evolutionary neuroscience, marmoset *(Callithrix jacchus)*, reproducibility

## Abstract

IntelliCage for mice is a rodent home-cage equipped with four corner structures harboring symmetrical double panels for operant conditioning at each of the two sides, either by reward (access to water) or by aversion (non-painful stimuli: air-puffs, LED lights). Corner visits, nose-pokes and actual licks at bottle-nipples are recorded individually using subcutaneously implanted transponders for RFID identification of up to 16 adult mice housed in the same home-cage. This allows for recording individual in-cage activity of mice and applying reward/punishment operant conditioning schemes in corners using workflows designed on a versatile graphic user interface. IntelliCage development had four roots: (i) dissatisfaction with standard approaches for analyzing mouse behavior, including standardization and reproducibility issues, (ii) response to handling and housing animal welfare issues, (iii) the increasing number of mouse models had produced a high work burden on classic manual behavioral phenotyping of single mice. and (iv), studies of transponder-chipped mice in outdoor settings revealed clear genetic behavioral differences in mouse models corresponding to those observed by classic testing in the laboratory. The latter observations were important for the development of home-cage testing in social groups, because they contradicted the traditional belief that animals must be tested under social isolation to prevent disturbance by other group members. The use of IntelliCages reduced indeed the amount of classic testing remarkably, while its flexibility was proved in a wide range of applications worldwide including transcontinental parallel testing. Essentially, two lines of testing emerged: sophisticated analysis of spontaneous behavior in the IntelliCage for screening of new genetic models, and hypothesis testing in many fields of behavioral neuroscience. Upcoming developments of the IntelliCage aim at improved stimulus presentation in the learning corners and videotracking of social interactions within the IntelliCage. Its main advantages are (i) that mice live in social context and are not stressfully handled for experiments, (ii) that studies are not restricted in time and can run in absence of humans, (iii) that it increases reproducibility of behavioral phenotyping worldwide, and (iv) that the industrial standardization of the cage permits retrospective data analysis with new statistical tools even after many years.

## 1 Introduction

IntelliCage® is a home-cage system with four operant conditioning boxes integrated into the corners of the housing cage and has been marketed since 2003. The design of the IntelliCage was developed by neurobehavioral scientists experienced in mouse testing since 1978, which then was turned into an industrialized product by NewBehavior AG (Zürich). The need for such a system was rooted in four initially independent threads.

### 1.1 Dissatisfaction with standard phenotyping approaches

Firstly, there was a growing dissatisfaction, or more poetically, a disenchantment, with the interpretation of classic mouse behavioral tests, as discussed in detail by Lipp and Wolfer (2022). This was not based on lack of publications. On the contrary, in the early 1990s, the group of Hans-Peter Lipp and David Wolfer at the University of Zürich (Switzerland) ran one of the few behavioral laboratories specialized in testing mice, which resulted in many cooperative projects that were published in high-ranking journals. However, conceptually, the field disintegrated rapidly. One reason was the uncritical adaptation of tests designed originally for rats and then transferred to mouse neuroscience and behavioral genetics. By and large, a mouse test battery included a mix of operant and fear conditioning tasks with various maze procedures reflecting different cognitive theories. Yet, the tests employed were presented in a piece-meal fashion depending on whether they fitted a specific interpretation. In extreme cases, behavioral outcomes after genetic manipulations were considered by molecular biologists merely as an icing on the cake, not infrequently accompanied by withholding behavioral data questioning the hypothesis, or by not citing contradictory publications. Unfortunately, no one wondered how mice behaved and learned naturally and how this might fit with the laboratory data. At least in rats, the work of the Blanchards in Hawaii permitted interpreting various rat behaviors in ethological terms (Blanchard and Blanchard, 1988), while early approaches of assessing the behavior and interactions of electronically identified mice in interconnected mouse cages were never followed up (Ely et al., 1972, 1976). Thus, interpreting mouse behavior became largely a theory of how mice ought to behave, categorizing movements of mice as proxies for hypothetical brain processes.

For example, a series of studies had focused on behavioral differences associated with a minor variation of the hippocampal mossy fiber system, the extent of the infrapyramidal mossy fiber (IIP-MF) distribution. Earlier studies had shown that genetic and epigenetic variations of this trait were correlated with behavioral test scores as observed after hippocampal lesions (Lipp et al., 1989; Schöpke et al., 1991; Bernasconi-Guastalla et al., 1994; Hausheer-Zarmakupi et al., 1996), yet other studies showed that the IIP-MF were also correlated with strength of handedness (Gruber et al., 1991; Hausheer-Zarmakupi et al., 1996) and intermale aggression (Guillot et al., 1994; Sluyter et al., 1994). The latter findings did not fit well with theories perceiving the hippocampus as a substrate for spatial memory and processing but were instead compatible with earlier theories postulating a generally inhibitory role of the hippocampus for behavior. Because of such ambiguity, the hippocampal community apparently lost interest and, for more than 25 years, the role of the IIP-MF in behavioral control remained mainly obscure and overlooked. Interestingly, the relation between mossy fibers and behavior has recently been investigated through IntelliCage (Bramati et al., 2023).

Similarly to the case of mossy fibers, our behavioral studies of knockout mice missing the prion protein PrP (Büeler et al., 1992) did not reveal any significant behavioral changes, in accordance with other functional studies (Weissmann, 2004; Castle and Gill, 2017). Given that we had used only a few classic tests, it was not clear whether the removal of the PrP gene had hidden negative side-effects preventing the use of the knockout technique as a method protecting animals from prionic infections, as shown much later for cattle (Richt et al., 2007).

Since much information about the ecological validity of behavioral data obtained in the laboratory was missing, a NATO conference was launched to discuss studying brain and behavior in semi-naturalistic environments (Alleva et al., 1995; Lipp and Wolfer, 1995; Nadel, 1995). Eventually, the Lipp/Wolfer group research group decided to set-up outdoor pens in Russia (Lipp and Wolfer, 2013), realized with the support of behavior geneticist Inga I. Poletaeva and bear researcher Valentin S. Pazhetnov. The first goal was to monitor natural selection as a tool to estimate the functional importance of missing genes or hippocampal mossy fiber variations. Later, they used the same pens to study learning processes of feralized mice outdoors (Dell'Omo et al., 2000; Lewejohann et al., 2004), which reinforced their intention to develop a test system more compatible with real world conditions. After all, house mice (*Mus musculus*) show amazing problem-solving abilities enabling them to adapt even to urban environments (Lipp and Wolfer, 2013; Vrbanec et al., 2021).

To be fair, the actual situation has changed by the rediscovery that the key to understanding mouse behavior in standard phenotyping and translational research is to study how mice act in social contexts and naturalistic environments (Smith, 2023), combined with analyzing their variable problem-solving strategies (Le et al., 2023). Most recently, the importance of an “ethological neuroscience” based on ethologically relevant behavioral tests has been emphasized by behavioral neuroscientist Raffaele d’Isa and neuroethologist Robert Gerlai (d’Isa and Gerlai, 2023). This interest in natural behavior of animals is now transferred to studies in humans, boosted by an NIH budget of 25 million USD to develop outdoor tracking of human daily activities (Smith, 2023).

### 1.2 Animal-unfriendly testing

The second reason to develop a more realistic yet animal-friendly test system was animal welfare. The field of behavioral testing of genetically modified mice emerged around 1990, facing the need to adapt test systems for mice that had been developed and used predominantly in rats. Among these tests, two did not fit mice’s evolutionary behavioral framework (the collection of instinctual behaviors) preparing them to cope with daily routines, namely the water maze (Morris et al., 1982) and shock-induced fear conditioning (Fanselow, 1994). Nonetheless, just these two rather stressing tests became standard procedures for assessing memory and learning of mice. Another main problem was the aversion of mice to being handled by humans, especially by males (Sorge et al., 2014; Georgiou et al., 2022), and their slow responding to various handling-habituation procedures. Finally, a large body of observations has shown that routine procedures such as transport to the test facility can increase plasma corticosterone levels up to 24 h after transport (Drozdowicz et al., 1990), while handling itself has mostly unpredictable effects on behavioral measures influenced by anxiety (Bailey et al., 2006; Deacon, 2006; Drude et al., 2011; Heredia et al., 2012; Lopez-Salesansky et al., 2016; Do et al., 2020; McCarson, 2020; Sensini et al., 2020; Marcotte et al., 2021; Hogue et al., 2022). Thus, minimizing handling of experimental mice would seem a useful strategy to apply in any kind of behavioral test (Wahlsten et al., 2003; Bailey et al., 2006).

### 1.3 Standardization and reproducibility problems

The field also realized soon that the results of behavioral studies could often not be replicated by other laboratories (Crabbe et al., 1999; Crabbe and Wahlsten, 2003) or, worse, failed replication in the own one, specifically for fear-related tests such as the elevated plus maze (Wahlsten et al., 2006). The simplest solution to deal with this problem was to avoid replication of experiments, as there was no foreseeable benefit in doing this, and to call for more stringent standardization, preferably by having others adopting one’s own methods. However, as most laboratories had developed their own protocols, and dimensions of apparatus differed with manufacturers, procedural standardization in the field faced resistance and strongly delayed acceptance of newly invented tests or protocols, specifically in the pharmaceutical industry with huge proprietary behavioral databases for drug testing. Thus, *procedural standardization* met a stalemate, only mitigated by the growing awareness for careful description of behavioral studies (du Sert et al., 2020). On the other hand, the progressive growth of veterinary control and services pushed toward *environmental standardization* in animal housing, resulting in strict control of illumination, temperature, and humidity, as well as minimized contact with humans and germs, thus constantly reducing environmental stimulation. It was obvious to most observers that housing single mice in cages containing only sawdust bedding represented a maximally impoverished environment, but even keeping mice in social groups was opposed considerably by reviewers of papers till a study could show that variation of group-house mice in behavioral test situations was not exceeding the statistical variation of individually housed animals (Wolfer et al., 2004).

### 1.4 Too much work with standard behavioral phenotyping

**Figure 1:**
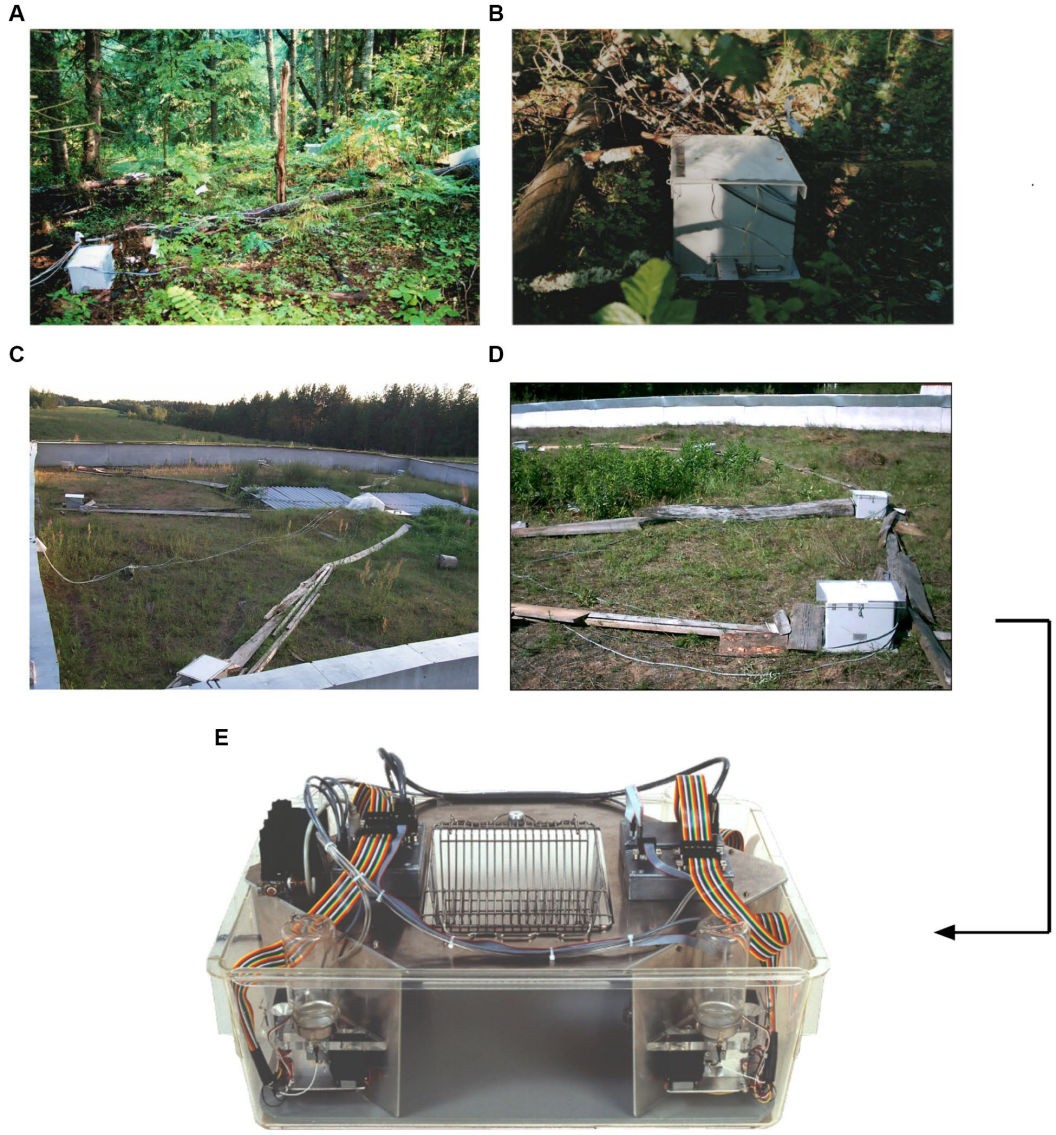
From outdoor feeder boxes in Russia to a tool in the laboratory. The conceptual origin of IntelliCage were feeder boxes placed in the forest or in outdoor pens for recording and controlling the patrolling of wild and feralized mice (Lipp and Wolfer, 2013). **(A)** Set-up of feeder boxes to study natural learning in wild mice. **(B)** Closer view of a feeder box in the forest. Experiments in the forest failed because feeder boxes were partially destroyed by roaming bear cubs smelling the mouse food. **(C)** Outdoor pen (20 × 20 m) in the Russian field station Chisti Les containing eight feeder boxes and a central computer controlling the boxes. **(D)** Closer view of an automated feeder box recording entries of mice tagged with transponder chips. Food was only delivered upon a new visit. **(E)** First prototype of an IntelliCage operating on MS-DOS, constructed by Alexei Vyssotski and Giacomo Dell’Omo. **(A,B)** Courtesy of Patricia D’Adamo.

The fourth and final reason to envision a new test system was very simple and practical. The number of mice used for behavioral research had exploded. From 1940 to 1989, a PubMed search for “mice” and “behavior” found some 1,800 papers, mostly referring to behavior genetics, drug testing and neuroscience, but only one paper reporting behavioral analysis of transgenic mice (Finger et al., 1988). From 1990 to 2023 the number of papers referring to behavioral testing of genetically modified mice alone rose to 28,000. Because of its previous activities, the Lipp/Wolfer laboratory was one of the earliest to have a comprehensive mouse test battery for collaborative efforts, but it was facing soon personnel and space limits, despite streamlining behavioral phenotyping by automated recording and data analysis. On average, the time to complete a standard manual phenotypic testing of 30–40 mice (including recording of spontaneous activity, water maze, radial maze, avoidance learning, and data analysis) took 3–6 person/months. Given the constraints of academic teaching, expanding the size and staff of the labor was not a satisfactory solution. Therefore, from 1998 to 2001, the Lipp/Wolfer laboratory intensified its efforts to develop a home-cage-based behavioral testing system that could be user- and animal-friendly by harboring mouse groups, permit efficient and automated high-throughput analysis of mouse behavior, and fulfill long-lasting standardization criteria at the procedural level. The goal was achieved in 2002 (Figure 1) when the system was first presented at the Society for Neuroscience Meeting and in journals (Bohannon, 2002; Gerlai, 2002). The IntelliCage was then marketed from 2003 to 2008 by the spin-off company NewBehavior (Zürich, Switzerland) and afterwards by TSE-Systems International (Berlin, Germany).

## 2 Review body

The following sections will:

- Briefly describe the outdoor studies which provided important input to the design of the IntelliCage
- Discuss the IntelliCage’s design features and provide a comprehensive description of the most recent IntelliCage system, currently lacking in the literature
- Review early validation studies from 2003 to 2007
- Present selected papers illustrating some principal uses of the IntelliCage and review the relations between water maze and IntelliCage
- Sketch the degree of acceptance of the system and present some past and upcoming research lines, including a discussion of its inherent limitations
- Describe extensions of the IntelliCage with other home-based analysis systems
- Outline the adaptation of the IntelliCage to larger species and the potential incorporation of new features
- Indicate the present and future state of high-level data analysis in the IntelliCage.

The final conclusions will summarize the insights that the IntelliCage system has brought to the field, chiefly from a conceptual point of view. Our review intends to complement rather than replace an earlier review of the IntelliCage system based on publications till 2018 (Kiryk et al., 2020), which includes discussion of several fundamental studies not analyzed here.

### 2.1 Three proof-of-principle outdoor studies

While discussing the potential advantages and costs of the resource-consuming project that would have later lead to the development of IntelliCage, it was clear that such a system would be met with skepticism. The foreseeable main objection would be the belief that behavior of mice must be studied by separating them, because their social interaction would be a confound factor and make the results unreliable. The origin of this idea is not documented. Likely, it reflects a tendency to standardize behavioral testing thoroughly by excluding any external distractions, possibly also the Western culture habit of separating students for exams. Hence, it was necessary to show that mouse behavior as observed in the laboratory can also be assessed reliably under uncontrollable environmental conditions and in social contexts. The condition for this approach was the identification of individual animals by means of radiofrequency identification (RFID), made possible in the mid 1990s by the availability of implantable glass transponders. The technique was refined in two studies (Dell'Omo et al., 1998, 2000). Mice lived for prolonged periods in subterranean shelters from which they could roam and visit feeder sites at varying places. The feeders were either of simple types (just a circular antenna around some mouse food) or more complex ones that could deliver (or withhold) a food upon entry of a transponder-tagged mouse. This allowed simple spatial learning and assessment of patrolling patterns by replacing the feeders. The Lipp/Wolfer group also realized that access to feeder boxes must be strictly restricted to single individuals, because they were expecting that a mouse would visit a feeder, be identified inside, and be rewarded with a small portion of grains. Yet, the mice surprisingly outsmarted the researchers by visiting the boxes in small groups and sharing the food portion (Dell'Omo et al., 2000). Lipp and collaborators conducted three outdoor studies.

In a first, only partially published, study transgenic mice ectopically expressing the neural cell adhesion molecule L1 in astrocytes (Kadmon et al., 1990) were investigated in the laboratory for water maze learning (Lipp and Wolfer, 1998). Overall, the differences were subtle but hinted to a superior flexibility of the transgenic mice after platform reversal. A batch of mice of either sex (49 transgenics and 22 wildtypes) was then transferred to Russia and released in an outdoor pen for studying survival (Vyssotski et al., 2000). A spatial learning study was then performed by placing food at variable distances from the central shelter. For 18 days, mice were fed in the shelters, then food was exclusively placed in the most distant locations, followed by some changes in placements. The first replacements showed that the transgenics appeared faster at the new sites (*p* < 0.05), thus confirming the conclusions of the water maze study (see Supplementary Figure S1).

A second study used a similar approach (Lewejohann et al., 2004). The mice had been genetically modified by eliminating a non-messenger RNA coined BC1 (Skryabin et al., 2003). BC1 RNA is a small non-messenger RNA common in dendritic microdomains of neurons in rodents and is probably an evolutionary novelty in a rodent ancestor dating back 110 million years ago. Thus, it was hypothesized that the mice should have intact evolutionary old mechanisms governing escape and spatial learning, but that mutants lacking the molecule might show deficits in exploratory behavior requiring a more finely tuning of simple spatial and escape behaviors. Different lines were tested in three laboratories, and one line was also transferred to Russia for studying long-term survival and outdoor learning abilities. The laboratory tests showed unimpaired spatial learning in the mutants, while tests aimed at assessing exploratory behavior revealed deficits in the BC1-KO mice. When tested in the Russian outdoor pen according to similar schemes as the L1 transgenics, the mice deficient in BC1 appeared significantly later at newly placed food sites, confirming the results from the three laboratories.

**Figure 2:**
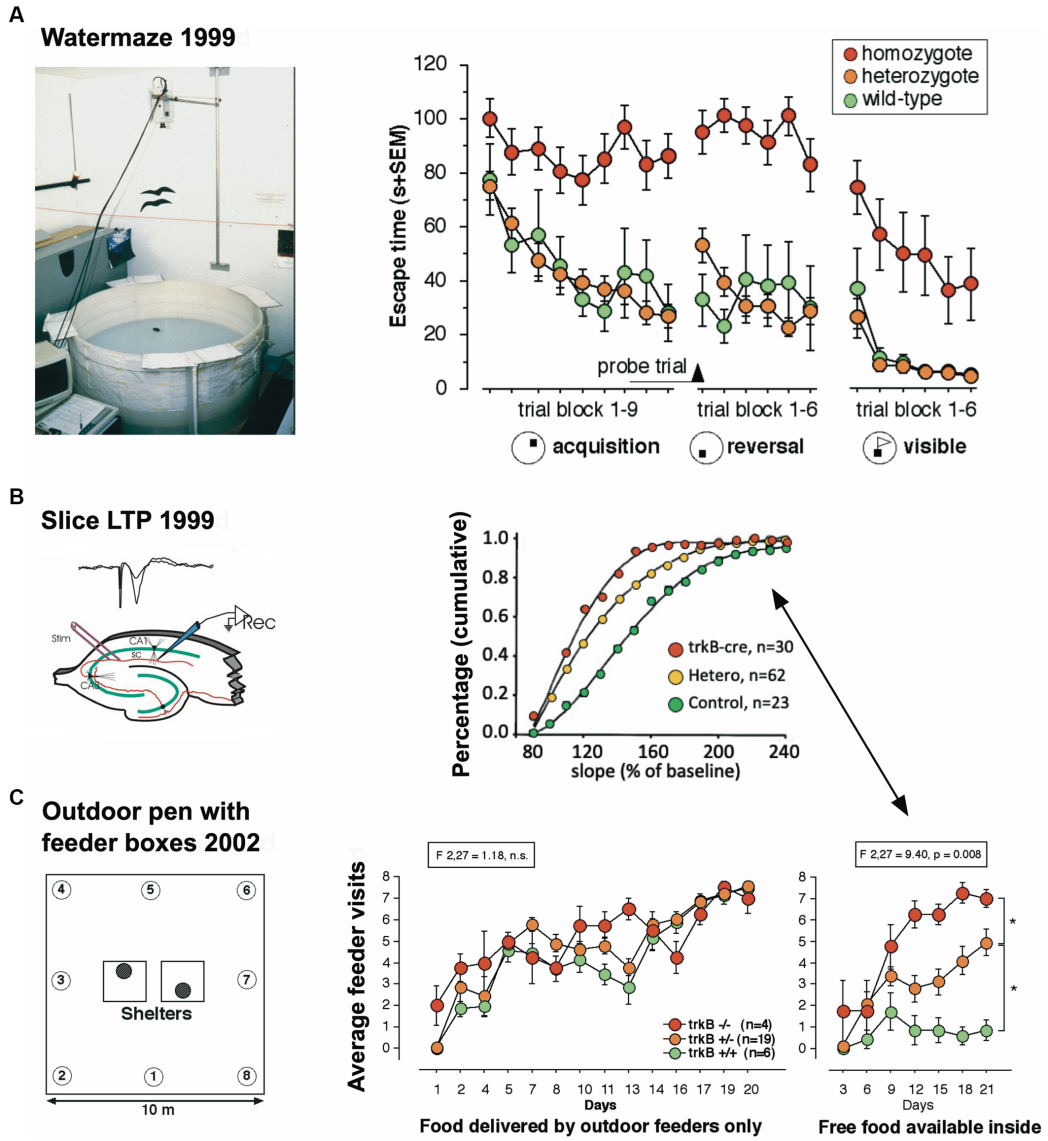
An eye-opening study comparing the spatial learning of trkB mutant mice in the lab with the behavior in a semi-naturalistic situation **(A)** trkB mutant mice were tested for water maze learning and showed a severe impairment, mostly visible in the homozygous mutants, while the heterozygous mice behaved like the controls. Modified after Minichiello et al. (1999). **(B)** In the same study, hippocampal slices had shown intermediate LTP values for the heterozygous animals. Modified after Minichiello et al. (1999). **(C)** Outdoor patrolling behavior of the same trkB mutant line in the Russian field station Chisti Les over 21 days. The mice had to patrol 8 boxes to obtain maximal food reward. Every third day, patrolling the loaded boxes was not necessary as food was placed inside the shelter, offering an opportunity for a one-day place reversal learning. Notably, the homozygous mutants ignored this opportunity, which was instead regularly exploited by the wildtype controls. Intriguingly, the heterozygous mutants felt in-between the groups, as would have been expected from the LTP data. Modified after Vyssotski et al. (2002a).

The third study focused on a mouse model in which the receptor trkB for the brain derived neurotrophin factor (BNDF) had been eliminated postnatally, resulting in mice in which the loss of trkB was restricted to the forebrain (Minichiello et al., 1999). These mice underwent standard behavioral tests in the Lipp/Wolfer laboratory, showing no differences in passive avoidance, no memory impairment in contextual freezing, and only minor impairment on the radial maze, while improvement in two-way avoidance learning hinted at hippocampal deficits (Jarrard, 1980). In the water maze, however, the homozygous mutants were unable to learn the task due to strong thigmotaxis (wall hugging) that even persisted when the escape platform was visually marked (Figure 2A), while the wildtype and heterozygous mice could not be separated statistically. Mice were also investigated for changes in long-term potentiation in hippocampal slices. Here, all genotypes were statistically different from each other (Figure 2B), suggesting that the presence of one functional allele for trkB was mitigating the LTP impairment. A batch of mice was then transferred to Russia for outdoor testing in a radial maze equivalent, in which eight boxes were grouped around two central shelters (Figure 2C). Transponder-tagged mice of all genotypes were tested over 21 days for development of correct box visits, just one visit per box/day being allowed. Because of the potential memory problem of the homozygous mutants as evidenced in the water maze, every third day food was placed inside the shelter to prevent starvation. This was not a complete reversal because the outside boxes were still active (Vyssotski et al., 2002a). All mice learned the task, but on the days with free food inside, wildtype mice quickly abandoned outside patrolling and ate the food inside, whereas the homozygous mutants just continued their usual patrolling. Intriguingly, the heterozygous animals were significantly different from both wildtype and homozygous mutants, corresponding to the earlier LTP data.

These three studies showed that genetically dependent behavioral differences observed by single mouse testing in the laboratory were replicated in outdoor studies. While the differences in the L1 and BC1 study were not dramatic, they were in the same direction. One would have expected that a weak phenotype would disappear under largely uncontrollable outdoor conditions, but this was not the case. Moreover, the outdoor testing of the trkB mutants showed much more precise results as the intermediate scores of the trkB heterozygous mice corresponded exactly to their intermediate position in LTP scores. This was unlikely a chance event. The main lesson was clear: *patrolling of feeder boxes or conditioned patrolling over 20–40 days without human interference gave the same results and came to the same conclusions as many weeks of daily single mouse testing in conventional test batteries in the laboratory*. Another lesson was that the main behavioral factor distinguishing the various genotypes in pens were *problems in spatial reversal learning and switching strategies*. Overall, the pen data reflected the real cognitive problems of mice, namely finding nutrients in a familiar territory under daily changing conditions, yet without facing shock grids or inescapable ponds, and this justified the development of test systems emulating the daily world of mice in natural conditions.

### 2.2 IntelliCage: design features for a home-cage system housing mice in a socially enriched environment

Before presenting the IntelliCage in detail, we consider here the design features derived from practical experience in the laboratory and outdoors. The outdoor studies implied that the system: (a) needed to run without human supervision for 2–3 weeks with minimal handling; (b) should present retreat opportunities allowing some separation of non-sociable mice; (c) should have at least four sites for patrolling; (d) should provide access to reward sites at which mice could be identified individually; (e) should present a simple set of sensory stimuli guiding patrolling and choices at a given location.

However, the conditions found in laboratories or mouse facilities required restrictions or changes. First, the system ought to be easily stored and cleaned. Therefore, we chose a commercially available rat cage (model 2000 of Tecniplast, Buguggiate) and equipped the IntelliCage with four red mouse houses of the same company, allowing to separate non-sociable mice during rest. Other additional equipment required (Makrolon cages, water bottles and nipples) was available from standard laboratory providers. To minimize disturbance of mice and facilitate cleaning, the plate holding all apparatus could be lifted and placed on a cage with new bedding but could be decomposed easily for maintenance. The corners to be visited needed to be controlled individually, so most of electronic circuitry was placed inside them, remaining connected to a main controller located on a plate closing the top opening of the cage. A tubular RFID antenna with an inner diameter of 30 mm limited access to a corner for a single mouse. The antenna tube was placed at a height of 58 mm which was easily accessible for climbing into it, while the corner was free from bedding material. In contrast to the outdoor pen, we decided to use liquid as reward, because this allowed for quantifiable delivery of water solutions to identified subjects for controlled periods of time. Delivery of food reward cannot be controlled that way as pellets are carried around and can be eaten by cagemates. Since most small-scaled dry mazes offering reward face problems with partial reinforcing (the mice do not care to move on after a wrong choice), we also added an air-puff system delivering a moderate, non-painful, punishment depending on adjusting the valves for pressurized air available in most laboratories. Such air-puffs can also serve to expel mice taking corners for sleeping places. In terms of controllable sensory stimuli, we decided to present them only in form of simple visual LED patterns or differently tasting liquids. This required the placement of two bottles per corner, each one freely available or, depending on the experimental protocol, potentially only accessible by nose-poking.

### 2.3 System description

#### 2.3.1 Hardware

**Figure 3:**
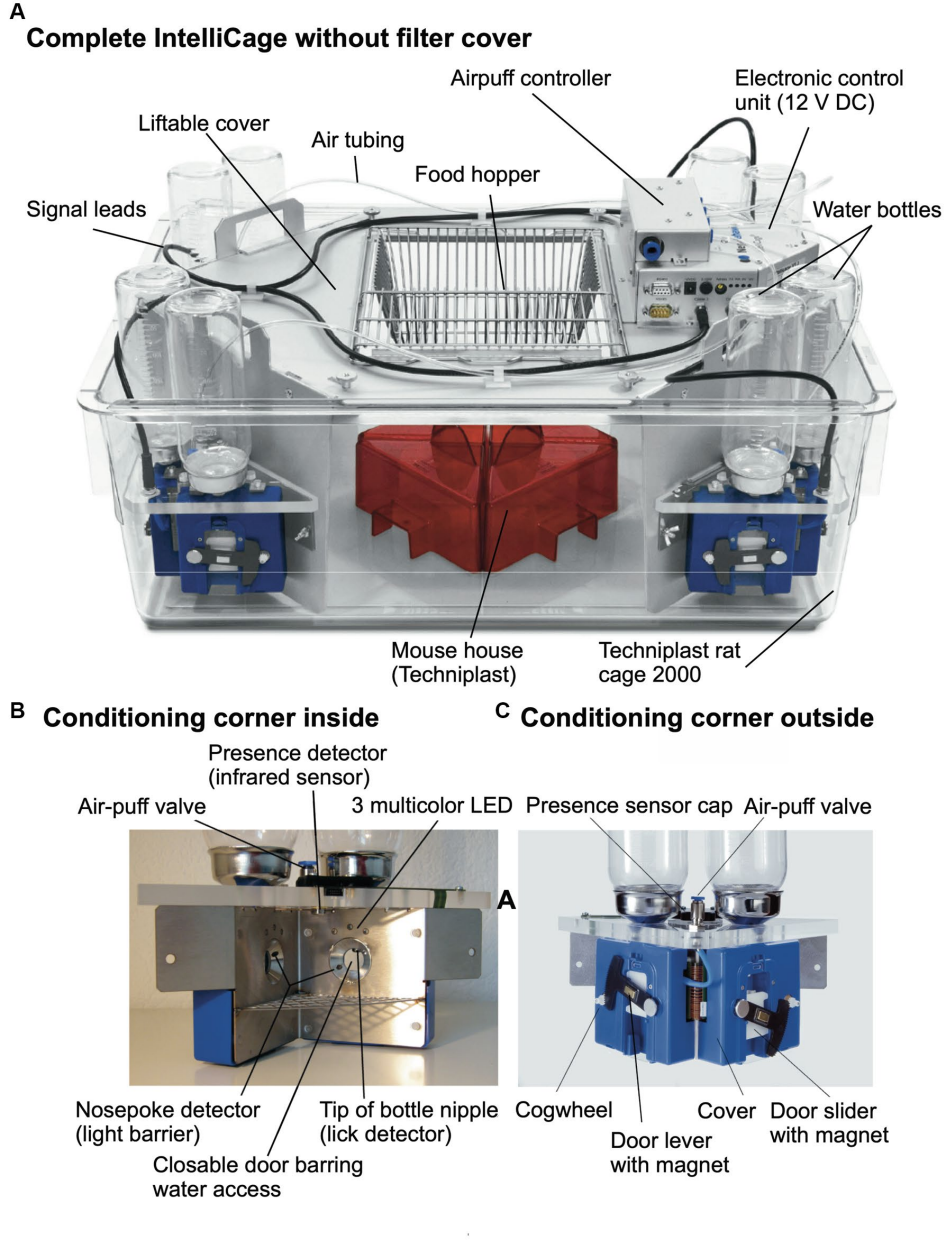
Modern IntelliCage since 2006. **(A)** Complete view of the system integrated into a commercially available polycarbonate rat cage (20.5 cm high × 58 × 40 cm at top, 55 × 37.5 cm at bottom, Tecniplast 2000, Buguggiate, Italy). The entire cover plate with the corners can be lifted for cleaning or exchanging the cage body. The electronic control unit integrates light and temperature sensors. It connects with up to 8 IntelliCages running the same or different programs. **(A)** Combination of 4 standard Tecniplast mouse-houses permits preferential huddling of mice. **(B)** Inside view of the conditioning corner faced by the mouse when advancing through the ring antenna. Walls, nose-poke-holes and grids are made from stainless steel. **(C)** Outside view of the conditioning corner. The sliding doors are moved by means of a cogwheel-operated mechanism preventing squeezing of the mouse nose. Part of the operating circuitry is integrated in the blue plastic container.

Figure 3A shows the most recent industrialized version of the IntelliCage that was developed from the prototype shown in Figure 1. Each corner contains a motherboard running a firmware that sends the signals from the sensors (RFID, temperature, light barrier, lickometer) to the main controller board on top of the cover plate and sends input from the main controller to the actors (LED, door sliders and valves delivering air-puffs). The hardware settings allow for conditioning of mice by sensing the activity of individuals and acting by applying the appropriate responses. The RFID antenna and the temperature sensor together identify the *presence* of a mouse in the corner, *nose-poking* is recorded by breaking an infrared light beam crossing the opening to the bottles and *licking* activity can be registered when the mouse uses muzzle or tongue to touch the drinking nipples of the water bottle (Figure 3B). In response, *door opening/closing* can be initiated via the door slider, *LED lightening can be induced* and some *air-puff* can be given (Figure 3C). LED and door control can be exercised independently on the two sides per corner, hence allowing for left–right, as well as gustatory, discrimination conditioning. These constant input/output options ensure replicability and standardization over time and testing with other species in different environments. More information about hardware and some of its peculiarities are found in Supplementary Figure S2 (Dos and don’ts in the IntelliCage).

#### 2.3.2 Software

**Figure 4:**
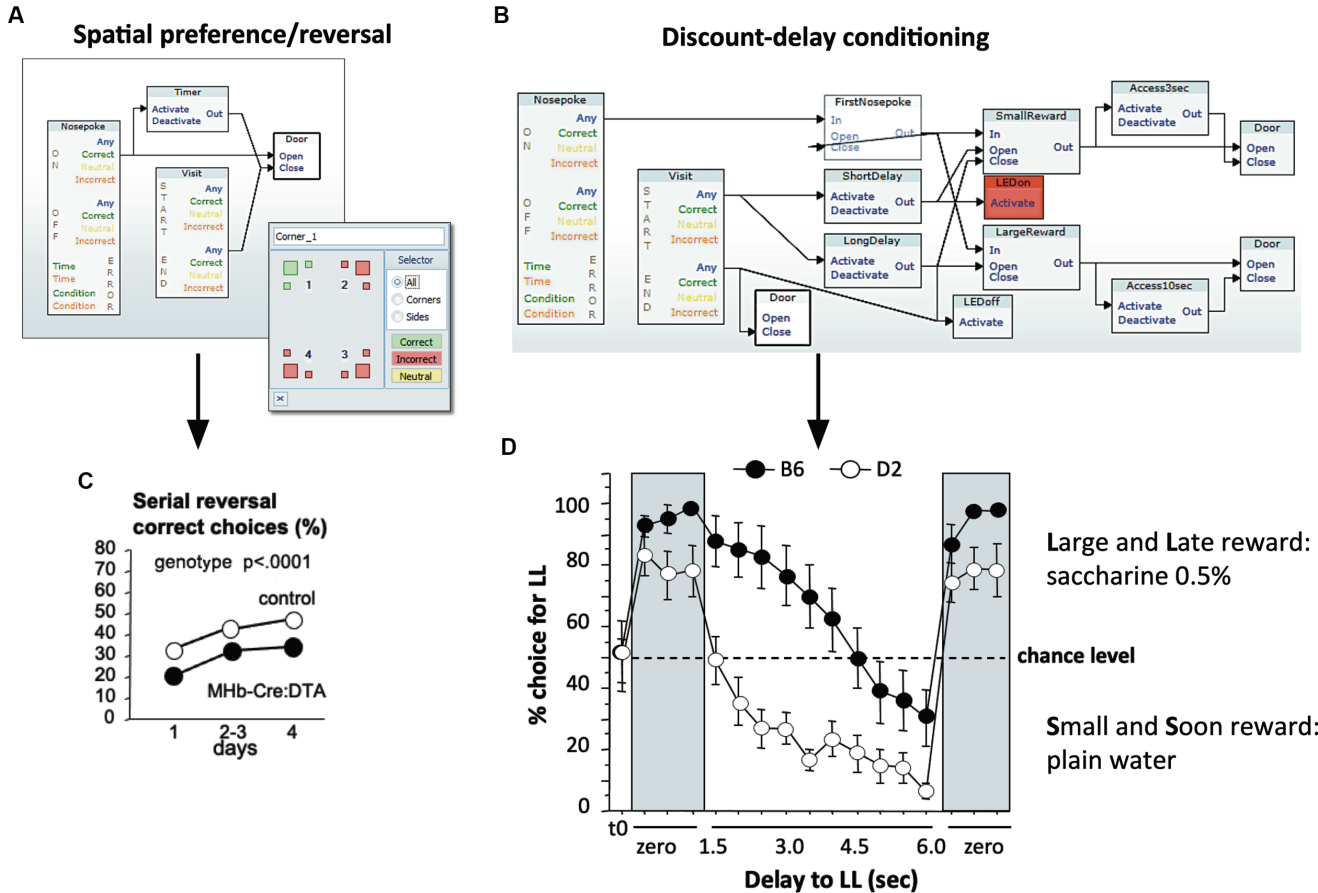
Designing simple and complex tasks in the designer program with a graphic user interface (GUI). **(A)** Graphic design for spatial learning. This requires a simple sequence: a specific corner is assigned to one or several animals. Upon identification of an assigned individual, a timer is activated and the door leading to the drinking nipple opens. The door closes after a defined period or after the mouse has left the corner. **(B)** Graphic design for discount delay-conditioning. This procedure measures how well mice can solve a conflict between easy access to plain water and the need to wait a defined time for obtaining a sucrose/saccharine reward. Upon entering a corner, the mouse is identified, two timers are activated according to the learning progress of the mouse, and an LED signal is activated to mark the beginning of the procedure. After having made a nose-poke choice towards one of the bottles, the system will deny access to the sweetened bottle if the nose-poke is too early. The recording of the animal’s actions indicates its ability to inhibit learned local movements, yet also a sense for time at short-term scales. **(C)** Data example of simple spatial programming: MHB-Cre:DTA mice carrying a mutation causing postnatal ablation of medial habenular cells are impaired in their ability of spatial reversal learning, however combined with other behavioral deficits (Kobayashi et al., 2013). **(D)** Strain comparison using discount-delay conditioning. C57BL/6 and DBA/2 mice typically differ in their ability of controlling behavior under conflicting situations (Wolfer et al., 2012). Saccharine preference was established rapidly in both strains when there was no imposed delay. Upon increasing waiting times, DBA/2 mice quickly switched to drink plain water, while C57BL/6 mice maintained a preference for saccharine, also with increasing waiting times, but eventually switched to the plain water solution. Presenting immediate reward re-established the saccharine preference in both strains. Example set up by Elisabetta Vannoni.

The unique feature of the IntelliCage system is its flexible software architecture that has remained largely unchanged for 20 years. Its central piece is the *Designer* application that sets the response of the system to mouse behavior by uploading a file generated with a proprietary graphical user interface (GUI). Here, units representing actors, sensors, and other instrumental operators can be logically connected using drag-and-drop functions. Many named designs can be constructed and stored by the user, and their activation sequence can be interconnected logically or by temporal schedules. Figure 4 shows examples for the two classes of protocols typically run in the IntelliCage, patrolling and local operant conditioning. For spatial learning, one or several mice have access to water in a defined corner only (Figure 4A). An example of data obtained in this way is shown by the results of a study using serial reversal learning behavior of MHB-Cre:DTA mice lacking medial habenular cells (Kobayashi et al., 2013). These mice showed an impaired ability of spatial reversal learning (Figure 4C), combined with other behavioral deficits, specifically higher impulsivity as also shown in the IntelliCage. To assess impulsivity and processes depending on inhibitory control, the protocols are more complex, as shown for a discount-delay procedure that measures how well mice can solve a conflict between easy access to plain water and the need to wait a defined time for obtaining a sucrose reward (Figure 4B). Interestingly, this procedure is able to identify strain differences (Figure 4D). These two programming examples demonstrate the ability of the IntelliCage to test simultaneously behaviors related to patrolling and to analyze locally the ability of problem-solving. Further graphic examples of such control files can be found under^1^.

**Figure 5:**
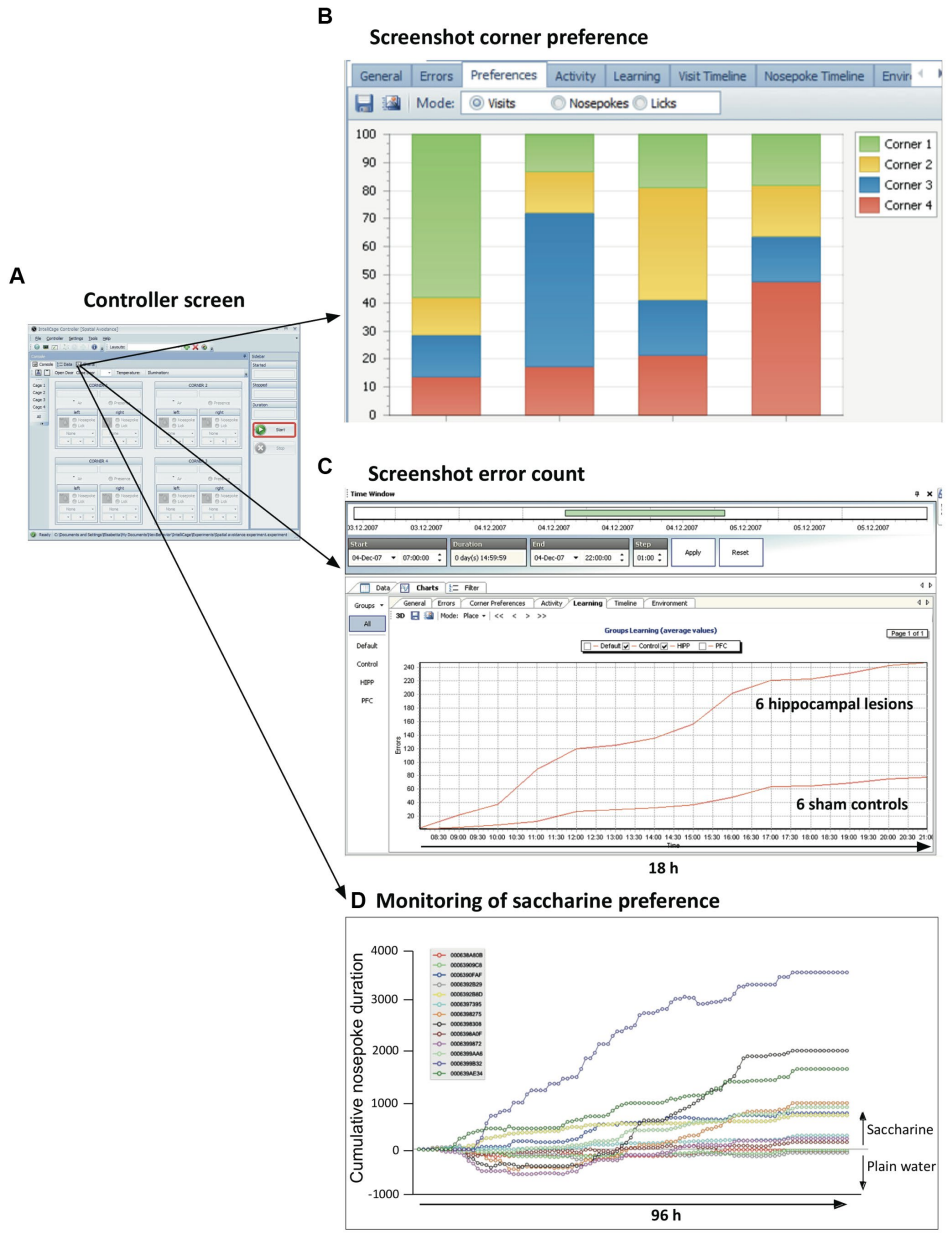
Ongoing information provided on-screen by the controller. **(A)** The default controller screen just shows the activity state of sensors and actors. Yet, the menu provides numerous opportunities to call the actual state of the data in both alphanumerical and graphic form. The graphs can be selected for single animals, subgroups, or all mice in the cage. **(B)** Quick monitoring of corner preferences by individual mice. **(C)** Continuous monitoring of behaviors considered as errors or success permits to recognize developing trends resulting from treatments. The screen shows the mean cumulative error rate in reversal learning as observed in a group of mice with hippocampal lesions. **(D)** Individual learning or preference curves can also be plotted, e.g., for saccharine preference. Note that the final mean score of the animals in the cage is around 900, because some of the mice ignored or even avoided saccharine. Also note that every experiment can be graphically replayed (from archive files), for individuals or for treatment groups, by using selectable time windows from seconds to weeks, thus recognizing the development of odd behavior patterns of treatment groups or strangely behaving animals.

The ***Controller application*** responds to the three inputs (visits, nose-pokes and licks) according to an experimental file assembled by the designer program (see above). Opening doors is seen as rewarding, closing or not opening doors as negative punishments, air-puffs as positive punishment, and LED light configuration were supposed to potentially convey information for instrumental conditioning, or can be used to deter animals. The Controller also presents to the user real-time information in the control panel (Figure 5A). The data are constantly assembled and analyzed using simple statistics showing the progress of the experiment, either for a single mouse or as group average. For example, checking the frequency of corner visits permits determining most or least preferred corners for a given mouse (Figure 5B). The controller can also present ongoing cumulative learning curves that show whether the scores of two experimental groups (such as hippocampally lesioned mice and their controls) coincide or diverge (Figure 5C). The behavior of individual mice can also be singled out. For example, plotting individually the saccharine preference (which can be obtained by presenting pairs of bottles containing either plain water or saccharine) rapidly identifies mice with strong preference, ambivalence, or even initial avoidance of the sweet taste (Figure 5D). Yet, the final collective scores indicated a weak yet significant preference for the entire sample. Other screens show actograms, separately for visits, nose-pokes and licks, and the time course of temperature and illumination. All graphs can be interactively shortened or expanded to inspect different phases of mouse activity during the experiment, from very short time windows to day-long plots. The registered data are stored continuously on the PC as text files and turned into zipped files (archives) when the experiment is stopped by exiting the Controller run. The archive files themselves cannot be manipulated, following the recommendation of good laboratory practice (GLP).

Post-experiment visualization of observations and some basic statistical views are provided by the *Analyzer application*. This can read as many archive files as intended, *and replay the entire experiment and its* var*ious stages*, while the user selects the parameters for creating the tabular outputs. These may include subsets of animals, selected time windows, or types of responses such as licks, corner visits and outputs. The tables can then be transferred to a variety of statistical programs or databases such as Excel but must be further analyzed by the user. The entire palette of graphs that were produced by the controller during the experiment can also be obtained in Analyzer.

**Figure 6:**
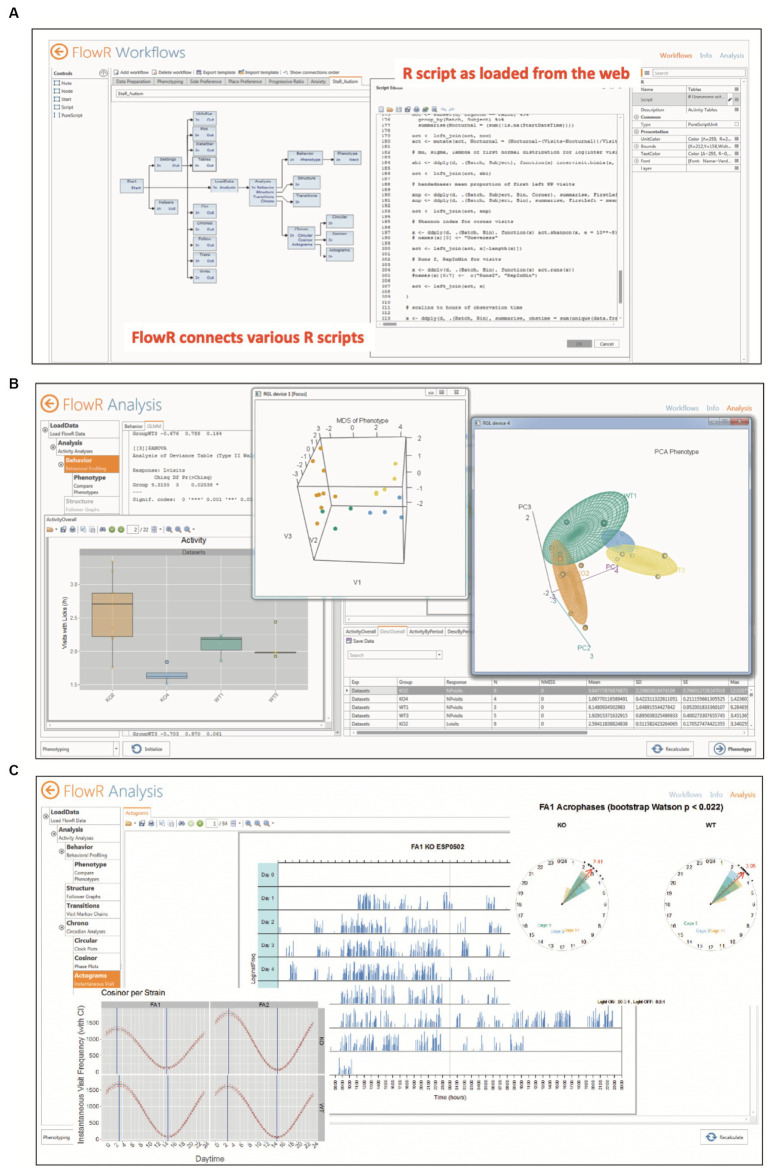
Automated statistical analysis of IntelliCage data by FlowR. **(A)** Graphic interface for creating a workflow connecting various R scripts for simple or complex statistics. The program reads in archive files from IntelliCage experiments, leaving the original data intact. **(B)** The extracted data are read-inand analyzed by pre-assembled R-routines including publication-ready graphic displays and statistical analysis in PDF format. Shown here are simple bar graphs, and 3D multidimensional scaling and principal component analysis. The analysis requires a minimum of computer experience and knowledge in R or other statistics programs. **(C)** Chronometric analysis including simple activity plots, cosinor analysis and vector rose plots of acrophases for rapid comparison of groups. Picture provided by courtesy of XBehavior.

For in-depth data analyses, however, one would turn to a *pre-assembled software* that allows for detailed data preparation and sophisticated statistical analyses even for users inexperienced in applying statistical software themselves. To our knowledge, there are three software packages facilitating such data analysis, two based on Python (Dzik et al., 2018; Ruffini et al., 2021) and one using R (Voikar et al., 2018). All of them can extract and rearrange data from IntelliCage archive files, but for Python applications, the statistical analysis is left to the user’s skills, for example Esmaeili et al. (2022). On the other hand, FlowR (XBehavior, Bänk, Switzerland) is based on a graphic GUI combining R-protocols (Figure 6A), that has been developed by the same persons having implemented the Programmer and the Designer application for the IntelliCage, being thus familiar with the architecture of the data as well as with the behavioral meaning of the protocol files. It also includes pre-assembled advanced statistics (Figures 6B,C), so that persons inexperienced in statistics can just import the archive files for getting the statistics with a few clicks^2^. Moreover, it has been used in a variety of IntelliCage studies (Fischer et al., 2017; Hardt et al., 2017; Ajonijebu et al., 2018; Vogel et al., 2020; Simmons et al., 2021a; Tran et al., 2021; Hahnefeld et al., 2022; Stephan et al., 2022; Vasić et al., 2022; Hühne-Landgraf et al., 2023).

### 2.4 Early validation studies

**Figure 7:**
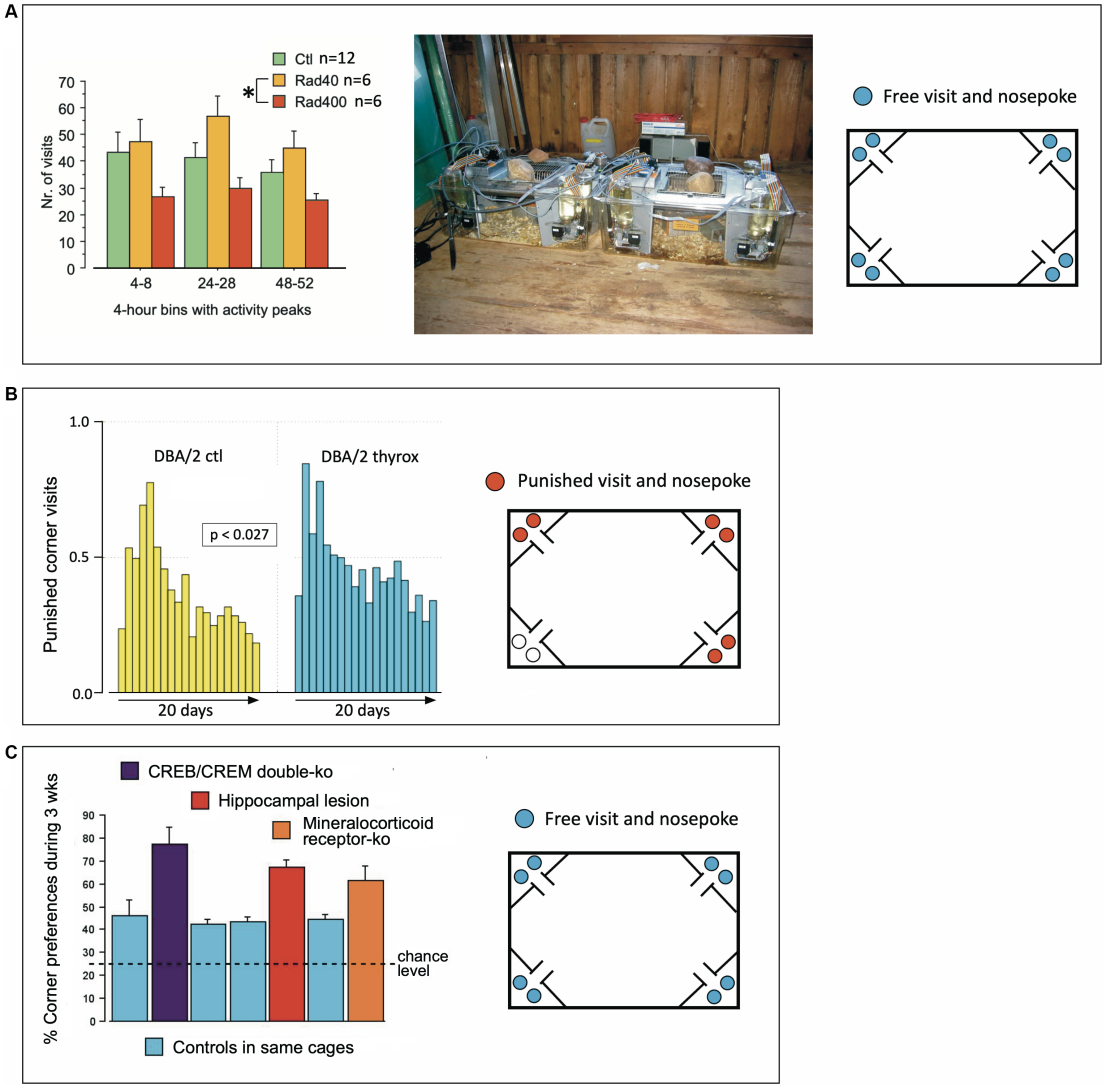
Early validation studies of IntelliCages in 2003 and 2004. **(A)** Estimating robustness of expected differences in a largely uncontrollable environment in a Russian field station. Two old-type IntelliCage were used to run a pilot study with cranially irradiated mice, but the information provided by the radiologists was lost due to the untimely death of Nada Ben Abdallah who was running the study. However, an IntelliCage data archive file could be recovered (by Pascal Zinn) and permitted to run a data analysis using the stored information only. There were clearly some differences between treatment groups that cannot be interpreted, however. On the other hand, the data demonstrate that IntelliCages can reveal significant behavioral differences between treatment groups even in noisy environments. The same cages were also used in that year to study differences between wild voles and mice (Galsworthy et al., 2005). **(B)** IntelliCages revealing extremely subtle transgenerational effects. Two IntelliCages housed 20 female DBA mice, 9 controls and 11 animals whose grand-grand-fathers (3 generations ago) had received postnatal thyroxine injections that changed brain and body features that were transmitted paternally (yet variably) over 3 generations of dam-raised DBA/2 mice. For details see Vyssotski et al. (2002b) and Vyssotski (2011). The observed behavior was how frequently the mice were visiting corners where they received air-puffs, which was rarely observed in other studies. The cages were situated in a non-climatized laboratory. Given the heat of summer 2003, we suspect that some mice were actively seeking the air-blows, which in this context provided a rewarding cooling. Modified after Lipp (2005) and Lipp et al. (2005). **(C)** Pooled presentation of non-systematic IntelliCage tests with knockout mice provided by collaborators and not being used in conventional tests, including a few mice with hippocampal lesions available for pilot studies. CREB/CREM double mutants and mice with knockout of the mineralocorticoid receptor were provided by Peter Gass and Thomas Lemberger in Heidelberg. Data were presented repeatedly by Lipp (2006) and Wolfer et al. (2012).

Novel systems need time to be accepted by peers or reviewers. In a phase from 2000 to 2004, the earlier versions of the IntelliCage system were tested using various mouse models. This could not be done by systematic studies, but the Lipp/Wolfer laboratory had access to a variety of mouse models that were sent for testing or were leftovers from other studies. From these mice, samples could be used for proof-of-principle studies showing the potential results with graphs to be presented at conferences and meetings. However, some of these earlier studies provided interesting insights as shown in Figure 7.

As one of the advantages of the IntelliCage was the opportunity of testing non-domesticated rodents (since handling during behavioral assessment is not required), two systems were shipped to Russia for studying wild mice from the local populations around the field station and were employed successfully in comparing bank voles (*Clethrionomys glareolus*) against wood mice (*Apodemus sylvaticus*), resulting in a first peer-reviewed IntelliCage paper (Galsworthy et al., 2005). To this end, the IntelliCage had to be placed in a rather primitive and largely uncontrollable environment, namely a log cabin serving as animal house for field studies. Because behavioral test systems are usually run under visual and acoustic isolation in special boxes, there were some concerns whether the observed species differences might not simply reflect uncontrollable events such visitors and outdoor noises. To check this objection, one of the students there, the late Nada Ben Abdallah, had obtained a batch of irradiated Russian mice for a pilot study checking different radiation intensities and their effects on spontaneous activity over a short period. The IntelliCages were placed in the same environment (Figure 7A). The data showed systematic differences that remained without scientific value as it was impossible to verify posthumously the details of the treatments. Yet they showed again that the IntelliCages were able to recognize systematic group effects in partially noisy and uncontrollable environments. Of note, however, is that IntelliCages were used later to reveal irradiation-induced behavioral changes (Barlind et al., 2010; Karlsson et al., 2011; Huo et al., 2012; Roughton et al., 2012; Ben Abdallah et al., 2013; Kalm et al., 2013, 2016; Osman et al., 2014; Kato et al., 2018; Sato et al., 2018).

IntelliCages proved their sensitivity in detecting subtle behavioral changes in DBA/2 mice whose grand-grand-fathers had received postnatal thyroxine injection, supposed to trigger transgenerational changes in brain and behavior (Vyssotski et al., 2000; Vyssotski, 2011). Because these mice had undergone different behavioral standard tests before and could not be used for further studies, they were placed in summer 2003 for a curiosity-check in IntelliCages placed on a table in a histology lab for 20 days. The simple task only required the mice to consume water in a specific corner, by punishing with air-puffs visits to other places (a task which is normally learned quickly by mice). However, these mice showed a persistent error rate that was also audible because of regular hissing of the air-blowers. The error rates even rose after 10 days, and were, for this period, significantly higher in the offspring of the ancestors treated with thyroxine (Figure 7B, see also Lipp, 2005). Because the summer 2003 was exceptionally hot and the laboratories were not climatized, we suspect that the mice sought some cooling and that the air-puffs could have become rewarding, which would explain the persistent error rates. However, at present the cause of the behavioral group difference detected by the IntelliCages remains obscure. IntelliCages used later also discovered epigenetic or paternally transmitted behavioral changes (Gapp et al., 2014; Ajonijebu et al., 2018), proving the sensitivity of the system.

During a collaborative project, the Lipp/Wolfer laboratory tested the effects of lacking CREB (cAMP responsive element binding protein) on mouse behavior (Balschun et al., 2003) and received from the same laboratory that generated the mutants a set of older mice that were carrying a double mutation (CREB/CREM) for preliminary testing. Likewise, some mice and their controls with a CreLox-deletion of the mineralocorticoid receptor (MCR) were also available from a collaborating laboratory (Berger et al., 2006), and the Lipp/Wolfer laboratory had some mice with hippocampal lesions and their controls from its own studies (Voikar et al., 2010). Because of different treatment history and age of the mice, they were tested only for adaptation behavior over 4 weeks. The common feature characterizing the mice with various malfunctions of the brain was clearly a high degree of corner preference (Figure 7C), while the control mice included in four cages showed practically equal results despite of their different backgrounds. Repetitive visits of the same corner were later found in more detailed analysis of mice with hippocampal lesions (Voikar et al., 2018) and appear to be a simple yet reliable sign of substantial cerebral malfunction in rodents. Normal mice show a preference for one or two corners, and patrol the others occasionally, so that abnormally high corner preference during the adaptation period can easily be detected on screen (Figure 5B).

### 2.5 Influential studies promoting the use of IntelliCages

Here we present and discuss some papers that were important for the acceptance and understanding of the IntelliCage system.

#### 2.5.1 Differential activation of neurons in the mouse amygdala according to motivation and learning task

**Figure 8:**
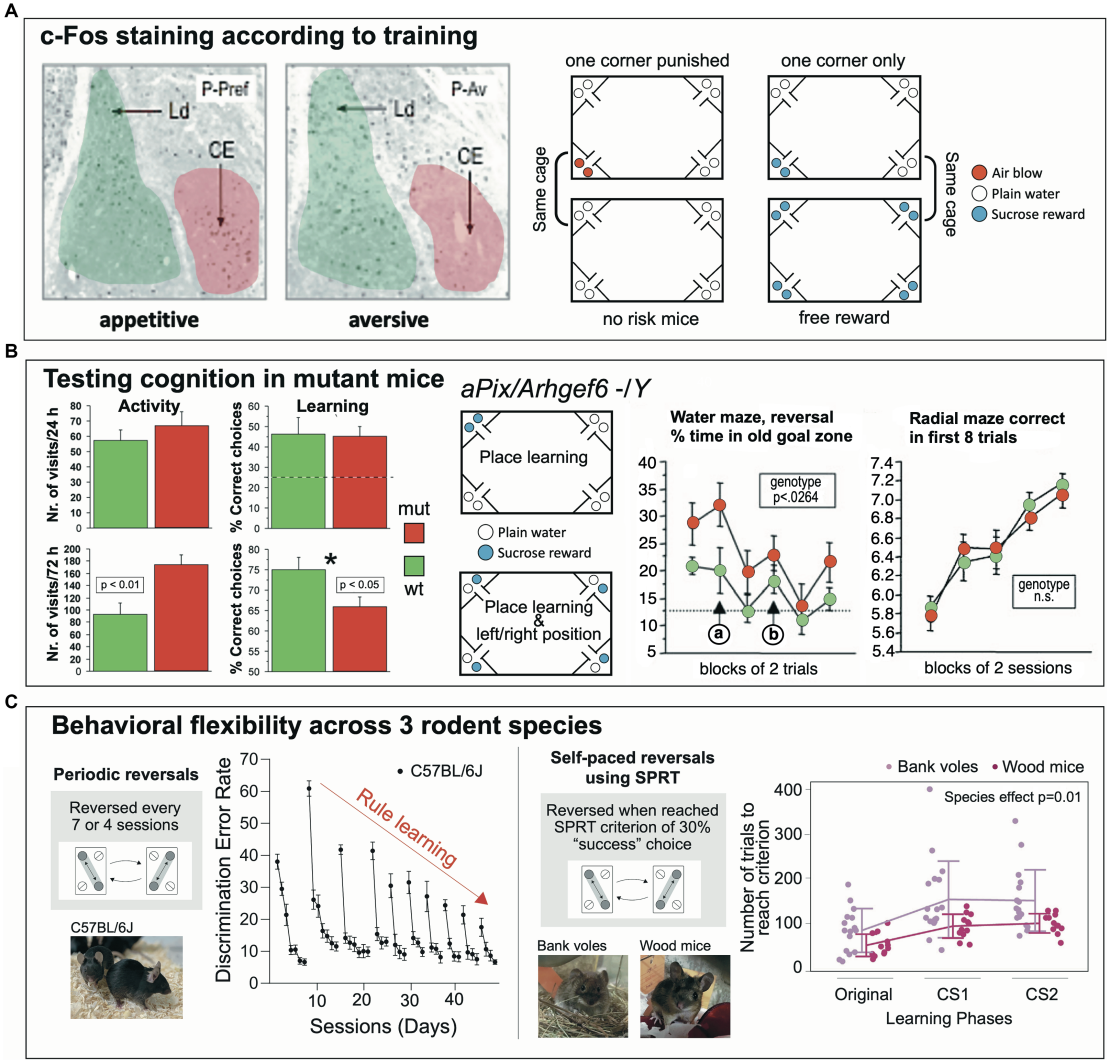
Influential studies giving rise to different directions of IntelliCage use. **(A)** The central amygdala (CE) shows activation of neurons as indicated by c-Fos-Expression when mice in an IntelliCage had to learn to visit a corner to obtain sweet reward. Their companions in the same cage, that had free access to sucrose solution in all corners, did not show activation of the central amygdala, indicating that the c-Fos activation was not due to a gustatory sensation. In a second cage, one group of mice had access to plain water in all corners, while their companions received air-puffs after having visited their preferred corner (as identified during the adaptation period). This study showed that the IntelliCage can provide unique testing procedures for dissecting the involvement of neuronal structures in motivationally different tasks during similar learning requirements tasks. Figure redrawn after Knapska et al. (2006); see also there for methods. CEm, central nucleus (amygdalae) medial part; Ld, lateral nucleus (amygdalae) dorsal part; P-Av, place avoidance task; P-Pref, rewarded place preference learning. **(B)** Mutations of the gene Arhgef6 in humans are known for causing intellectual disability. The corresponding mouse model underwent a series of behavioral tests including IntelliCage tests. In a simple place learning test, mutants were more active but learned the simple task as the wildtypes. Complicating the task by introducing left/right differences in the corners was associated with increased activity of the mutants, associated with higher error rates. Water maze learning showed modest differences, but the radial maze did not. Figure redrawn from Ramakers et al. (2012). **(C)** Two paradigms of behavioral flexibility based on learning a switching routine for obtaining water. The initial task was devised by Endo et al. (2011) and included learning a shuttling routine between diagonally opposite corners. After several sessions (usually days), the position of the active corners are switched and the mice must relearn the new positions, thus providing a measure for *spatial reversal learning*. The error rates after a new reversal are initially high, but gradually decline after every reversal, providing a measure for *rule learning*. As this protocol is time consuming, new versions were developed by one of us (Toshihiro Endo), based on a self-paced reversal (SPRT), usually after a mouse has reached a criterion between 30% correct responses. This less tedious (automated) procedure is particularly suitable for older animals and different wild species hard to test in common behavioral laboratories due to handling difficulties. For example, wood mice (*Apodemus sylvaticus*) learn this procedure easily as compared to bank voles (*Clethrionomys glareolus*). The power of this IntelliCage approach is that higher cognitive abilities of rodents can be assessed subtly and without stress. Graphs were modified after Endo et al. (2011) and Jörimann et al. (2023).

One of the first studies was conducted by Ewelina Knapska at the Nencki Institute of Experimental Biology in Warsaw (Poland) to analyze whether the central amygdala (CEA) in rodents (a connective bottleneck and a chief output structure to subcortical structures) was specifically involved in signaling rewarded learning, against a prevailing concept perceiving the amygdala as processing aversive and fear-related signals (Knapska et al., 2006) (Figure 8A). Learning-dependent activation of neurons in the amygdala nuclei was visualized by the c-Fos technique. The hypothesis predicted that the CEA would be selectively activated during rewarded, yet not during fear-related learning. Handling stress had to be avoided, and the experiment needed to be completed fast. Therefore, the IntelliCage system was chosen. Technically, the approach was demanding because learning-dependent c-Fos activation can only be observed during a short time window of 1–2 h, which required that the mice had to learn rapidly a spatial preference or avoidance task, and that the controls were also consuming water or sucrose solutions without learning. Thus, mice were divided in two groups and assigned to an IntelliCage for preference or for avoidance learning, respectively. During an adaptation period, access to liquids was restricted and only allowed for 3 h, which caused high corner visit activity necessary to establish a rapid place preference learning. During this period, the individual corner preferences of the mice were also established. For the c-Fos test, all bottles in the reward test cage contained a sucrose solution and half of the mice in the IntelliCage could consume sucrose wherever they wanted. However, for the other half, access to sucrose was only permitted in their least preferred corner, which required a rapid place learning against their earlier spatial preference. In the IntelliCage assigned to avoidance learning, half of the mice could consume plain water wherever they wanted, yet the other half received air-puffs when visiting their preferred corner, enforcing avoidance of this location. The results obtained with this very elegant design of balancing motivations and learning requirements showed then that c-Fos activation of neurons in the CEA occurred chiefly after having learned a spatially defined sucrose preference, but not in the mice that consumed reward everywhere. Conversely, avoidance-dependent spatial learning did not entail c-Fos activation, nor was it increased in the controls showing consummatory drinking of plain water only. This study showed that IntelliCages could be used successfully in tackling complex neurobehavioral questions.

#### 2.5.2 Subtle re-arrangements of cues in the IntelliCage reveal impairments in mice generated as model for intellectual disability

Mutations of the gene *Arhgef6* in humans are known for causing X-linked intellectual disability (Figure 8B). The constitutive knockout mouse model of this syndrome underwent a series of behavioral tests including IntelliCage tests (Ramakers et al., 2012). Water maze learning did show modest differences, but not the radial maze. In a place learning test in the IntelliCage, mutants were more active, but learned the simple task as rapidly as the wildtypes. However, the task was then complicated inasmuch the mice not only had to learn the position of a rewarded corner, but also whether the left or the right bottle in a corner was providing water. This subtle change in task complexity was also associated with increased locomotor activity of the knockout mice, implying poor adaptation to a situational change. IntelliCages have also been employed in other mouse models of intellectual disability or autism (Viosca et al., 2009; Puścian et al., 2014; Fischer et al., 2017; Mitjans et al., 2017; Jensen et al., 2019; Syding et al., 2022).

#### 2.5.3 Assessing short-term flexibility and rule learning in 3 rodent species

Behavioral flexibility denotes the ability of animals and humans to adapt their ongoing behavior when facing environmental changes (Figure 8C). It does not only include a cognitive component but also various parallel adaptations of motor and motivational systems, which ultimately result in a decision whether an ongoing motor activity is maintained or changed (Lipp and Wolfer, 2022). Because of such multi-level processing, it is unsurprising that impairment of many brain systems leads to gross or subtle impairment of behavioral flexibility, which is not easily analyzed. Especially, water maze data offer only limited statistical clues for interpretation (Lipp and Wolfer, 1998; Wolfer et al., 2004). On the other hand, the IntelliCage system provides opportunities for analyzing even subtle changes in behavioral flexibility. The initial task was devised by Endo et al. (2011, 2012) and included learning a shuttling routine between diagonally opposite corners. After several sessions (usually days), the positions of the active corners are switched, and the mice must relearn the new positions. This procedure provides two measures. After a new reversal, the error rates are high but decline rapidly, showing the ability of the mouse to adapt its behavior within a limited time, a classic *reversal task*. The second measure is the comparison of initial error rates after the reversal that gradually decline after every reversal, thus providing a rare measure for *rule learning*. As the original protocol is time-consuming, new versions of the test are based on a self-paced reversal, usually after a mouse has reached a criterion of at least 30% correct responses. This less tedious (automated) procedure allows for testing of older animals and different species hard to study in common behavioral laboratories. Wood mice learn this procedure rather easily as compared to bank voles, and this behavioral difference is associated with the size of their medial habenular nuclei (Jörimann et al., 2023). The power of this IntelliCage approach is that higher cognitive abilities of rodents can be assessed subtly and without stress. Since observed behavioral flexibility in patrolling probably depends on many brain systems, the IntelliCage allows for additional tests not depending on locomotion, for example by assessing the degree of impulsivity by a reaction time task in which animals must withhold a response for some time. Such a procedure identified higher impulsivity (or less patience) in the bank voles. Of note is that a similar coherence between behavioral flexibility measures and the impulsivity test (the reaction time task) was observed when analyzing the medial habenular system in mHB:DTA transgenic mice (Kobayashi et al., 2013).

#### 2.5.4 Induction of social stress and its assessment

By its design, the IntelliCage system aims at minimizing stress of mice and appears to be less useful for studies involving stress. Nonetheless, there have been several studies specifically focusing on stress (Branchi et al., 2010, 2013a,b; Kulesskaya et al., 2014; Bergamini et al., 2016; Milior et al., 2016; Mohammadi et al., 2017; Akbergenov et al., 2018; Serchov et al., 2020; Picard et al., 2021; Poggini et al., 2021; Li et al., 2023b; Nagaeva et al., 2023).

Most of them used IntelliCage as a tool for efficiently estimating sweet preference against plain water to obtain a measure of anhedonia following various exposures to external stress, while others used a variety of protocols documenting impairment of various forms of learning thought to be affected by stress. Among these, the study of Gapp et al. (2014) is of particular interest, as the stressing treatments were applied to the fathers, while an (unexpected) behavioral improvement was found in the offspring. Others used the IntelliCage itself to deliver subtle forms of social stress. For example, Branchi et al. (2010, 2013a,b) produced social stress in male mice by daily mixing the populations of two IntelliCages and could show that communal nesting in childhood mitigated the reduction in sucrose preference as observed in stressed yet normally raised mice. Likewise, mixing two strains of inbred mice (female C57BL/6 and DBA/2) increased, somewhat surprisingly, stress markers and anhedonia as measured by saccharine preference in C57BL/6 (Kulesskaya et al., 2014). One may note, however, that many strain differences in learning paradigms persisted even under induced social unrest, most likely because the prolonged observation times in the IntelliCage cancel short-term effects of social interactions.

### 2.6 Acceptance by the field and coverage of topics

**Figure 9:**
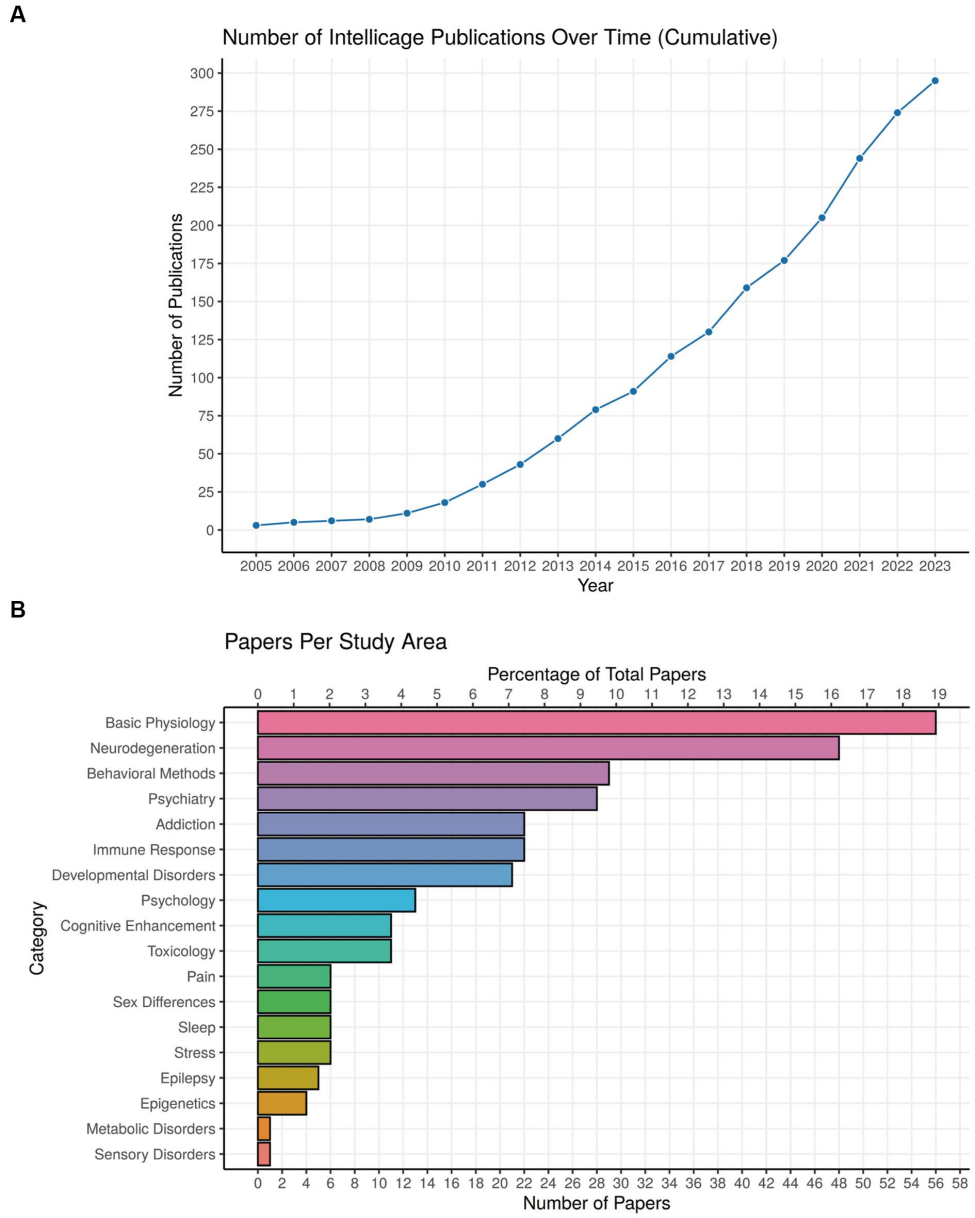
Use of IntelliCage systems in behavioral research. **(A)** Cumulative plot of papers dealing practically with or describing IntelliCages since 2005. The year 2023 includes publications at 15 October 2023, including some reviews and discussion papers. Searching criteria in Google Scholar (screening the entire paper) were: presence of the keyword “IntelliCage” together with (i) Primary journal articles that use “IntelliCage” as part of the methodology, (ii) Review papers/textbook chapters only if they focus on rodent behavior, (iii) Preprints (bioRxiv), (iv) Articles in languages other than English. Anything else is not included, for example conference abstracts, theses, articles that only mention “IntelliCage” without actual use or specific focus on it, etc. A complete list of papers, ordered alphabetically or chronologically, can be found in Supplementary References. **(B)** Proportions of IntelliCage papers classified according to scientific fields. For a description of the main disease classifications, see Table 1.

Some 20 years after its first presentation, the IntelliCage system has now been accepted widely, as the term is used even without reference to the trademark name (Plum et al., 2023). Likewise, its ability to produce equal experimental outcomes in different locations has been repeatedly verified (Lipp et al., 2005; Krackow et al., 2010; Codita et al., 2012; Kobayashi et al., 2013). After a period with low publication volume, the number of papers having used IntelliCage technology rose to 295 on October 15 2023 and is likely to reach 300 soon (Figure 9A). A focus of development has been the Nencki Institute of Experimental Biology in Warsaw, having summarized the work with IntelliCages up to 2018 (Kiryk et al., 2020). We have updated the main table of their paper focusing on behavioral protocols in Supplementary Table S1, and provide here primarily clinical classifications (Table 1; Figure 9B). A complete list of IntelliCage papers is provided in Supplementary References (for details see legend of Figure 9A).

**Table 1:** Publications between 2005 and October 2023 grouped according to main clinical fields.

Addiction (non-alcohol)	Skupio et al., 2017; Ajonijebu et al., 2018; Iman et al., 2021a,b
Aging	Mechan et al., 2009; Berry et al., 2012; Albuquerque et al., 2013; Too et al., 2016a; Fischer et al., 2020; Oizumi et al., 2020; Barranco et al., 2022; Li et al., 2023b
Alcohol abuse disorder	Radwanska and Kaczmarek, 2012; Parkitna et al., 2013; Smutek et al., 2014; Holgate et al., 2017; Mijakowska et al., 2017; Stefaniuk et al., 2017; Beroun et al., 2018; Koskela et al., 2018, 2021a,b; Iman et al., 2021b; Stefaniuk et al., 2021; Hühne et al., 2022; Pagano et al., 2022; Caly et al., 2023; Frycz et al., 2023; Nalberczak-Skóra et al., 2023; Stefaniuk et al., 2023
Anxiety	Safi et al., 2006; Jensen et al., 2016; Sano et al., 2016; Fischer et al., 2017; Raab et al., 2018; Sasaki et al., 2023
Brain lesional or ischemic damage	Voikar et al., 2010; Vannoni et al., 2014; Voikar et al., 2018; Dzirkale et al., 2023
Chemical exposure	Onishchenko et al., 2007; Zhu et al., 2010; Endo et al., 2012; Ogi et al., 2013, 2015; Aung et al., 2016; Sano et al., 2016; Xia et al., 2016; Yang et al., 2017; Kimura et al., 2020; Wen et al., 2021; Sasaki et al., 2023; Zhu et al., 2023
Developmental disorders	Ramakers et al., 2012; Puścian et al., 2014; Fischer et al., 2017; Mitjans et al., 2017; Fröhlich et al., 2019; Jensen et al., 2019; Garrett et al., 2020; Horigane et al., 2020; Morello et al., 2020; Balan et al., 2021; Meng et al., 2021; Puścian and Knapska, 2022; Puścian et al., 2022; Syding et al., 2022; Viosca et al., 2009; Xiao et al., 2022
Immune system	Too et al., 2014a,b,c, 2016a,b; Cathomas et al., 2015a; Pan et al., 2019; Arinrad et al., 2021; Markova and Knyazheva, 2021; Wilke et al., 2021; Markova et al., 2022; Plum et al., 2023
Irradiation	Jaholkowski et al., 2009; Barlind et al., 2010; Huo et al., 2012; Roughton et al., 2012; Ben Abdallah et al., 2013; Kalm et al., 2013; Osman et al., 2014; Kalm et al., 2016
Mood disorders	Branchi et al., 2010, 2013a,b; Cathomas et al., 2015a,b; Alboni et al., 2016; Bergamini et al., 2016; Jastrzębska et al., 2016; Milior et al., 2016; Mohammadi et al., 2017; Kato et al., 2018; Marwari and Dawe, 2018; Poggini et al., 2019; Serchov et al., 2020; Serykh et al., 2020; Markova and Knyazheva, 2021; Poggini et al., 2021; Sun et al., 2021; Volkmann et al., 2021; Markova et al., 2022; Yamamoto et al., 2023
Neuro-degeneration	Kiryk et al., 2008; Rudenko et al., 2009; Codita et al., 2010; Kiryk et al., 2011; Oakeshott et al., 2011; Sekiguchi et al., 2011; Weyer et al., 2011; Oakeshott et al., 2012; Balci et al., 2013; Gumucio et al., 2013; Oakeshott et al., 2012; Ryan et al., 2013; Menalled et al., 2014; Stribl et al., 2014; Urbach et al., 2014; Alexandrov et al., 2015; Benraiss et al., 2016; Koss et al., 2016; Masuda et al., 2016; Simmons et al., 2016; Masuda et al., 2018; Rudenko et al., 2019; Mehr et al., 2020; Nieraad et al., 2020; Cisbani et al., 2021; Mifflin et al., 2021; Tikhonova et al., 2021; Winslow et al., 2021; Yesiltepe et al., 2022
Schizophrenia	Peltola et al., 2015; Nakamura et al., 2021; Mätlik et al., 2022; Stephan et al., 2022
Seizures and epilepsy	Orock et al., 2018; Liu et al., 2020; Wu et al., 2023
Traumatic brain injury	Muthuraju et al., 2012, 2013; Vogel et al., 2020; Lopez-Caperuchipi et al., 2021; Simmons et al., 2021a,b; Hahnefeld et al., 2022

Most studies using IntelliCages refer to basic physiology and neurodegeneration, chiefly by comparing specific genetically modified mouse models. A more diverse cluster of studies is the development of behavioral methods in the IntelliCage, often employed in translational psychiatry. The predominant topic in addiction studies is, unsurprisingly, alcohol abuse, because cage-mates can be exposed to different concentrations while monitoring the behavioral consequences directly. Mouse models with immune defects and developmental disorders have also been productively used, while other topics are less represented. The IntelliCage is also mentioned increasingly in patent applications (not listed here), indicating its usefulness as an unbiased behavioral system providing data from a standardized set-up everywhere in the world. Interestingly, true high-throughput phenotyping studies were infrequent, conducted chiefly by the industry (Oakeshott et al., 2012; Balci et al., 2013; Alexandrov et al., 2015), but laboratories performing longtime follow-up studies profited from the reduced iterative workload in phenotyping (e.g., Codita et al., 2010; Radwanska and Kaczmarek, 2012; Plank et al., 2016; Masuda et al., 2018; Iman et al., 2021b). Taken together, the diversity of scientific fields which have profited from the use of IntelliCage underscores the versatility of the system. We present a selection of papers according to clinical criteria in Table 1 and draw attention to useful reviews and discussions of the system using other criteria (Kiryk et al., 2020; Iman et al., 2021a; Varholick et al., 2021).

We are unaware of substantial criticism of the IntelliCage system, as home-cage-based testing systems are mostly well perceived by behavioral science and the public. However, a noteworthy perspective article by Crabbe and Morris (2004) about conflicting concepts underlying high-throughput testing questioned the need for automation and speed in animal testing, calling instead for heuristic reflections before action, according to a “festina lente” principle (acting too fast retards progress).

Given the high number of divergent IntelliCage papers and topics, we will refrain from discussing them further and we will focus on selected studies showing interesting directions. Readers interested in how the IntelliCage compares to the increasing number of home-cage-based testing systems can find tabulated comparisons (Kiryk et al., 2020; Mingrone et al., 2020; Voikar and Gaburro, 2020; Iman et al., 2021a; Coulibaly, 2022; Kahnau et al., 2023b).

### 2.7 Research trends in past and future

#### 2.7.1 Analyzing spontaneous activity – a simple but effective tool

Spontaneous locomotor activity in the home cage is a sensitive tool for assessing various pathologies. For example, simple movement sensors over the cages of single-housed mice permitted to distinguish and monitor the impact of various prion strains on spontaneous locomotor behavior after inoculation in mouse brains (Dell'Omo et al., 2002). Given that IntelliCages always require an adaptation period before conducting any study, Vannoni et al. (2014) compared 1,552 mice from 32 mouse models for their spontaneous behavior during a one-week adaptation period. The only variables assessed were visits, nose-pokes and licks. The data were then analyzed by factor analysis, that identified 11 factors accounting for 83% of the variance, which could be grouped into four clusters. One accounted for corner and side preferences (27%), a second for parameters describing activity during corner visits such as nose-pokes and visit duration (21%), a third one for drinking activities such as lick number and frequency (20%) and a fourth one related to ultradian activity variations. Because the study included large samples of inbred strains, it could prove high stability of strain comparisons over time, while the inclusion of many hippocampally lesioned mice showed that these mice could be easily recognized by the IntelliCage and that their abnormal behavioral profiles often coincided with mutations suspected to carry hippocampal deficits. These results might appear boring to specialists in mouse phenotyping but would justify a close look at the first week in any IntelliCage study using an adaptation period. Some mouse models with obvious hypo- or hyperactivity may account in part for these results, but recent video-computer analysis of mouse spontaneous behavior in a simple circular arena for a short timespan has recognized subtle non-reinforced fluctuations in activity that hint at a periodic self-regulation by striatal and dopaminergic systems (Markowitz et al., 2018, 2023). It would seem possible that the IntelliCage system is picking up such small motor idiosyncrasies of the mice simply by sampling their activity for a long time. Obviously, such data sets would profit from re-analysis by artificial intelligence.

#### 2.7.2 Embedding IntelliCages in phenotyping batteries: IntelliCage versus water maze

Increasingly more laboratories are now integrating IntelliCages in their standard test batteries, offering themselves or to collaborators refined behavioral analysis of mouse models. However, it took time to convince the field that the IntelliCage system was able to produce data that were fitting the results from other classic tests. Given the omnipresence of the standard Morris water maze test (MWM) in most phenotyping laboratories, reports having tested mice in parallel in both IntelliCages and other apparatus often included the water maze, which, being capable of detecting spatial impairments, is often taken as a proxy for hippocampal malfunction. In analogy, it was (and is) frequently assumed that deficiencies in spatial learning within the IntelliCage would represent an animal-friendly alternative to the rather stressful water maze procedure, which requires forced swimming. Therefore, we present a short overview of 25 identified studies having reported similar or dissimilar treatment effects in water maze and IntelliCage and we try to define the common denominator in both tasks.

Only five out of 25 studies reported discordant results. Male mice but not females exposed prenatally to methylmercury showed several behavioral deficits in the IntelliCage, yet not in the water maze (Onishchenko et al., 2007). Notably, the adult male cohorts were formed at the age of 4 weeks (see also male–female differences below). Viosca et al. (2009) investigated the behavior of a mouse model of the human Costello syndrome and found moderate impairment in the MWM. However, the IntelliCage was apparently only used to document the apparent hypo-locomotion of the mutant mice. Voikar et al. (2018) reported that LRRTM1-deficient mice (lacking a gene for a specific type of neural cell adhesion molecule) showed several behavioral peculiarities including an aversion to enter narrow tubes. This was associated with normal MWM learning but retarded acquisition of IntelliCage tests, most likely reflecting some form of claustrophobia. Koss et al. (2016) studied mutant Tau knock-in mice for progressive changes in cognitive development. They showed no differences in the MWM (except for swim speed in older mice) but reduced behavioral flexibility in the IntelliCage as indicated by impaired rewarded place reversal learning. Wilke et al. (2021) observed behavioral differences in mouse models of encephalitis aggravated by injection of diphtheria-toxin ablating pyramidal neurons (DTA). Mice after DTA induction showed hyperactivity and deficits in the water maze but, surprisingly, no significant treatment effects in the IntelliCage using various tasks.

Six studies were done in the context of simple screening for potential cognitive problems in mouse models without making specific functional predictions and showed no or rather subtle differences in the two behavioral paradigms (Kulesskaya et al., 2014; Netrakanti et al., 2015; Peltola et al., 2015; Roccaro-Waldmeyer et al., 2018; Festa et al., 2019; Arinrad et al., 2023). Lack of treatment effects then either reflect insensitivity of both MWM and IntelliCage in revealing deficits, or true absence of effects. In two cases, however, parallel testing was based on clear hypotheses. For example, Jaholkowski et al. (2009) tested cognitive versus sensory deficits in CyclinD2 mutant mice lacking adult neurogenesis and found no impairment in the MWM and IntelliCage, yet deficits in olfactory tasks. Likewise, d’Isa et al. (2011) clarified a long-standing controversy about the role of the RasGRF1 protein in different knockout models, showing no spatial memory differences between mutants and wildtypes in both the water maze and IntelliCage protocols based on corner avoidance, while clear differences between mutants and wildtypes in contextual fear conditioning pointed at different roles of RasGRF1 in specific memory tasks.

A predicted similar loss of function in both assays, mostly in combination with a variety of other behavioral tests, was reported in five studies. Kiryk et al. (2011) analyzed the behavior of transgenic mice with a mutation of the human amyloid precursor protein (APP.V717I) at different ages. A deficit in spatial learning in both tasks was observed in all three age groups. However, the APP mice learned much better when co-housed with the wild-type littermates than when housed only with other APP mutants, suggesting a form of social learning that appeared to be modulated by different circadian activity of the transgenics. Lan et al. (2011) compared mice having undergone postnatal hypoxia and found deficits in punished reversal learning of males in the IntelliCage while the parallel deficits in the MWM approached significance only. A comprehensive behavioral phenotyping of the Ts65Dn mouse model of Down syndrome showed deficits in both tasks (Faizi et al., 2011). In the IntelliCage, these researchers used rewarded and punished learning for 4 days, removed the animals for 72 h and checked, as probe trial, corner preference and avoidance, the latter showing deficits in the mutant mice. Ryan et al. (2013) studied PLP1 triple knock-in Alzheimer mice at various age stages and reported deficits in both paradigms but noted that the IntelliCage was more sensitive in revealing impairments. Synaptic electrophysiology and hippocampus dependent behavior in mice lacking the cAMP-guanine nucleotide exchange factor II (cAMP-GEFII) were studied by Lee et al. (2015) who found impairment in long-term depression in hippocampal slices and moderate deficits in reversal learning paradigms in the MWM and IntelliCage.

Four papers in rats showed parallel loss of function. A study analyzing rats lacking the G protein-coupled estrogen receptor (GPER) reported that both female and male rats were slow to learn the MWM and showed modest impairment in place and reversal learning in the IntelliCage (Zheng et al., 2020). Cao et al. (2021) tested rats kept isolated after weaning in the MWM, IntelliCage and an own type of video-controlled dry maze, and claimed equal impairment, however without providing in-depth analysis. Li et al. (2023a) investigated the effect of juvenile isolation stress in 6 weeks old male and female rats, using ill-defined MWM tests and more extensive IntelliCage procedures. The authors claim deficits in the water maze and impairments in IntelliCage which include a reduced number of visits and nose-pokes in a punished left–right discrimination task and in the reversal test. A further paper analyzed postoperative cognitive dysfunction after splenectomy in aged rats, finding that operated animals were handicapped in both tasks after operation and that the pre-operatively administered drug Maresin appeared to mitigate such impairments (Li et al., 2023b).

Three studies reported parallel gain of function in both the MWM and IntelliCage. Konopka et al. (2010) induced a gene deletion (Dicer1) in the forebrain of adult mice that impaired, for a defined period, the transcription of non-coding messenger RNAs thought to be important for modification or stability of synapses. Somewhat surprisingly, the treated mice showed superior MWM learning including probe trial scores, and in the IntelliCage better sucrose-rewarded place learning. Schroeder et al. (2021) fed aged mice with the nutritional additive spermidine and found subtly improved spatial learning in the MWM and a trend also in the IntelliCage. Interestingly, the spermidine-fed mice were also better in a serial reaction time task permitting nose-pokes only during a visually signaled time window. Barth et al. (2023) tested mice deficient for the growth factor-like protein 7 (EGFL7), showing upregulated adult hippocampal neurogenesis. Both tests, learning and probe trial in the water maze and learning/reversal learning of corner preferences or avoidance in the IntelliCage, were slightly improved in the knockout mice.

What conclusions can be drawn from these studies? Clearly, gain of function in both tests is the most compelling argument that a common cerebral factor or process is underlying parallel behavioral changes in the MWM and IntelliCage. Probably this brain process relates to behavioral flexibility and not to a special form of memory. In the MWM mice must suppress inappropriate search strategies even when the position of the hidden platform is known (Lipp and Wolfer, 1998). Probe trials do demonstrate that mice have developed a spatial memory, but the usual scores only show how insistently they search over the old platform position (Wolfer et al., 1998), while impaired spatial reversal learning in the water maze is the most distinct behavioral sign after chronic hippocampal lesions (Lipp and Wolfer, 2022). Likewise, in the IntelliCage, learning the spatial position of the corners is rather fast, both by reward or by punishment, and treatment-dependent effects become visible mostly after positional changes, that is spatial reversal learning. Thus, in both tasks mice must adapt their movements to changing situations and the tests are excellent detectors for a variety of changes in brain structures of which the hippocampus is only one of many. Whether the type of spatial memory in the two tasks is equivalent is unknown. In the IntelliCage, its presence can be tested by removing and re-introducing the mice after some time. However, care must be taken to distinguish between punishing a visit from punishing a visit with nose-poke, as the former includes spatial memory and the latter combines memory for place with a special movement in that place (Voikar et al., 2010). Finally, from a practical point one should note that the motivational levels in the water maze are usually constant, while the IntelliCage permits to increase motivation for rewarded place learning by sweetening water or strengthening air-puffs. On the other hand, locomotor hyperactivity induced by treatments can confound IntelliCage testing but is less important in the water maze. To our knowledge, we are unaware of a study that, after having assessed specifically individual mouse behavior in the MWM and IntelliCage, analyzed intercorrelations between the two tests. In most of the cited studies, the two apparatus are part of a test battery, which is likely to complicate statistical analysis.

#### 2.7.3 Increasingly sophisticated protocols

The laboratory of Hannelore Ehrenreich in Göttingen (Germany) focused on developing sophisticated IntelliCage protocols to identify higher-order cognitive functions in mice (Mitjans et al., 2017; Pan et al., 2019; Arinrad et al., 2021), following several years of validation and protocol evaluation (Dere et al., 2018). Besides the usual assessment of various forms of patrolling, they developed a so-called “mental-time-travel protocol” (MTT). After adaptation to nose-poking for water, access time was limited to 2 h, and each mouse had to face one corner delivering air-puffs whose position changed in a predictable sequence over 4 days in a training cycle. The pattern was then repeated for a second round of 4 days and the preference to each corner on each day of the second round was used to assess MTT abilities. Each corner per day was considered either currently (=0 days after punishment), recently (=1 day after punishment), intermediately (=2 days after punishment) or longer ago punished (=3 days after punishment), and the data obtained were used to calculate a curve (percent corner preferences versus days after punishment) whose steepness (expressed as trendline) reflects the quality of the MTT thought to represent memory for traveled time and place.

#### 2.7.4 Immunology and gut-brain axis

Immunology and brain-gut interactions are a topic rapidly gaining relevance. Mice can be immunized by injection of ovalbumin (egg white) and will subsequently avoid drinking sweetened water if this contains ovalbumin (Cara et al., 1994). This simple paradigm was implemented in IntelliCages to study the role of mast cells in developing antigen-avoidance behavior (Plum et al., 2023). Plum and colleagues immunized, by intraperitoneal injection of ovalbumin, wildtype and knockout mice lacking mast cells, and placed them in IntelliCages to test them together. In each corner, one bottle contained a mix of ovalbumin and sucrose, the other plain water, but left/right positions were counterbalanced. Over 12 days, non-immunized mice from either control group developed a strong preference for the bottles containing sucrose and ovalbumin, while immunized mice with intact mast cells began to avoid the sweetened antigen-containing bottles increasingly. However, the KO-mice without mast cells maintained the sucrose preference, providing compelling evidence that mast cells were part of a signaling pathway for immunoglobulin E (IgE)-mediated allergies, transmitting antigen signals from the gut to the brain – amazing results and a top paper obtained with the help of a simple IntelliCage test.

#### 2.7.5 Testing mice with human genes

Ongoing studies by IntelliCage users are usually not communicated, but we anticipate some interesting results from Svante Pääbo’s laboratory in Okinawa (Japan), where transgenic mice carrying gene variants that are specific to modern humans and to Neanderthals are tested for their potential to change behavior. The outcome will be certainly of interest to a wide audience.

### 2.8 Inherent limitations and problems of the IntelliCage

Before discussing upcoming developments of the conventional mouse IntelliCage, some of its inherent limitations and problems should be addressed. Minor technical problems are dealt with in the Supplementary Figure S2 (Dos and don’ts in the IntelliCage).

#### 2.8.1 Testing females, males or both?

In most IntelliCage papers, only female mice were tested. One reason is that cages housing males over prolonged periods become smelly rather quickly. However, the main concern is intermale aggression and fights possibly interfering with behavioral testing. A systematic review by Varholick et al. (2021) analyzing traditional behavioral phenotyping and intermale aggression found little evidence for differences between dominant and subordinate male mice, which would justify the testing of group-housed males in IntelliCages. Assessing aggressive yet non-violent behavior between males (usually black C57BL/6 mice) in an IntelliCage would require expensive constant video-monitoring of individually recognized mice, as attempts by simple surveillance cameras did not identify aggressors (Mifflin et al., 2021). A possibly simpler solution might be to determine sleeping places and latrine areas (Makowska et al., 2019) by means of RFID tracking, as early studies in multi-cage systems have shown that subordinate and scarred male mice were forced to sleep in latrine areas (Ely et al., 1972), while recording movements in individually ventilated cages (IVC) cages housing 4–5 mice showed that male mice usually avoid the latrine area (Ulfhake et al., 2022). For more information about tracking, see the Section 2.10.2 “Tracking of mice in the IntelliCage” below.

Because IntelliCage studies testing both female and male mice using similar protocols are infrequent and not easily found by literature searches, they shall be discussed briefly. Clearly, they do not provide a coherent picture. There is a single study without genetic and treatment differences, showing that male mice develop a stronger preference for alcohol (Smutek et al., 2014). A developmental toxicity study assessing the neurotoxicity of the neonicotinoid acetamiprid did not reveal treatment effects on behavioral flexibility in the IntelliCage for both sexes (Sano et al., 2016), while exposing mice to early bisphenol A led to opposite treatment effects in corner visit patterns for adult males and females (Ogi et al., 2013). Saccharine preference of control mice in the IntelliCage was equal in both sexes (Morello et al., 2020).

More pronounced learning deficits in female mice as compared to males was observed in aged (16 months) CH3 mice that were exposed prenatally to arsenic (Aung et al., 2016), and irradiation of young mice caused a greater impairment of initial place learning in adult females than in males, in agreement with clinical observations (Roughton et al., 2012). In mice with a mutation of the AMBRA gene (thought to be linked to female autism), mutant females, but not males, lost preferences for sex pheromones as evidenced by connecting IntelliCages to boxes containing different scents (Mitjans et al., 2017).

**Figure 10:**
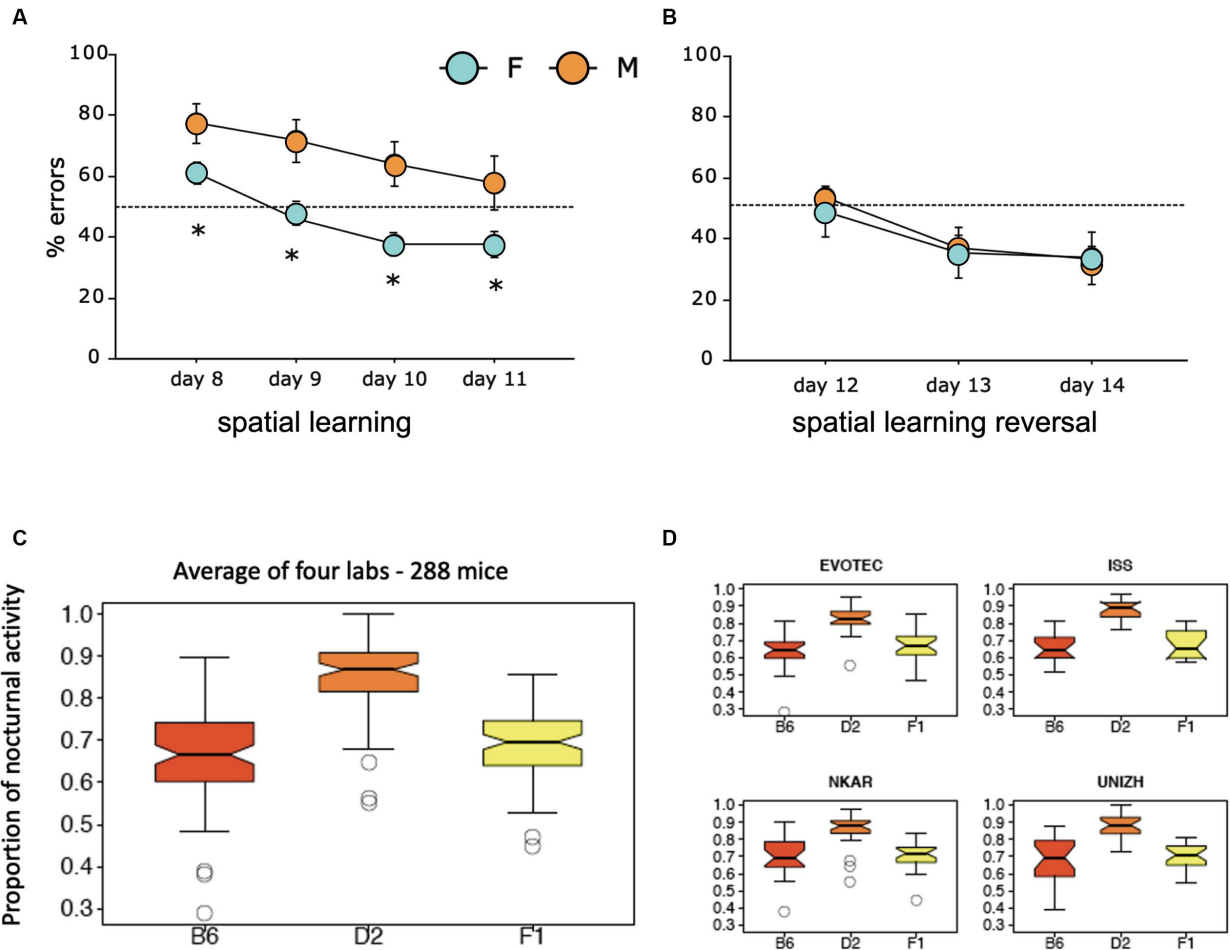
Male–female differences and inter-laboratory comparisons. **(A)** Significantly less errors in spatial learning of aged females (*n* = 15) as compared to males (*n* = 14), *p < 0.05, means and S.E.M. After 7 days of habituation to the IntelliCage environment with free access to all corners for drinking, water delivery occurred for 4 days only in the corner opposite to the one preferred during the adaptation period. **(B)** Reversal phase: during days 12–14, all mice underwent a 3-day spatial learning reversal where the only corner available for drinking was the preferred one during the adaptation phase (Berry et al., 2012). **(C)** Grand average of 288 mice of different strains (C57BL/6, DBA/2, F1 B6xD2) as observed for nocturnal activity in four different laboratories across Europe, indicating a highly significant strain effect (Krackow et al., 2010). **(D)** Laboratory-specific results show clear differences among the single strains at Karolinska Institutet (NKAR) in Stockholm, Evotec in Hamburg, Istituto Superiore di Sanità (ISS) in Rome; and University of Zürich (UNIZH), but the laboratory-specific comparisons revealed in all cases comparable strain differences. For details of the statistical design, see Krackow et al. (2010).

On the other hand, comparative IntelliCage testing often found increased resiliency in females toward treatment effects or mutations. Onishchenko et al. (2007) found behavioral differences after developmental exposure to methylmercury only in males and not in females. Mice having undergone sub-lethal hypoxia after birth showed, at the age of 6 weeks, moderately impaired spatial reversal learning in males but not in females, while both sexes showed persistent incorrect nose-poking in corners delivering air-puffs, yet less pronouncedly in females (Lan et al., 2011). Berry et al. (2012) investigated aged (21–24 months) male and female P66^Shc−/−^ mice, known for longevity, in the IntelliCage. They found higher initial exploration in mutant mice, yet more pronounced in females, while later testing for spatial learning revealed no genotype effects but better acquisition by the females (Figure 10A), surprisingly no more differences during reversal learning (Figure 10B). In an early life stress model, adult male mice showed treatment effects by being more subordinate than females in a water access competition test (Benner et al., 2014). Finally, Mifflin et al. (2021) compared 12-month-old male and female APP/PS1 and non-transgenic mice, and found that females performed better in a variety of IntelliCage tasks except for impulsivity tests. Taken together, there is no doubt that IntelliCages studies can recognize sex differences in a variety of tasks.

The studies above provide some hints that the basic motor activity of male mice is a main factor generating sex differences, but to better understand these differences, we must await for the results of an ongoing study comparing systematically male and female C57BL/6 mice in the IntelliCage. A crucial factor to stabilize social interactions in the IntelliCage is the procedure preparing both male and female mice for testing. The probably most important factor is the time span allowed for social adaptation within an IntelliCage-sized cage before recording and testing. We provide a detailed description in the Supplementary Figure S2 “Dos and don’ts in the IntelliCage.”

#### 2.8.2 Comparability between laboratories

Specifically designed studies have shown similar experimental outcomes in different laboratories, as stated before. However, it should not be inferred that the absolute values of activities in the IntelliCage are equal, but rather that the relative differences between treated mice or strains were similar in different places. This is exemplified in a large study (Krackow et al., 2010) in which laboratories in Stockholm, Hamburg, Zürich and Rome tested synchronously a total of 288 mice of the strains C57BL/6, DBA/2, and their F1 hybrids, raised and shipped by the same supplier. The statistical split-plot design assessed a variety of measures for differences between laboratories, strains and lab-by-strain interactions (see also in Krackow et al., 2010). For example, the grand average of the nocturnal activity scores of these mice for spontaneous nocturnal corner visits showed DBA/2 mice being clearly much more active than both other strains (Figure 10C), while absolute activity scores of strains could vary depending on place, but the strain order was the same (Figure 10D). Some years later, a transatlantic multi-lab study expecting to find equal motor activities of C57BL/6 mouse groups kept under stringent isolation in IVC conditions reported the same result: mice in three different labs showed significantly different behavioral activity level despite all efforts to environmental standardization and shielding (Pernold et al., 2019).

We consider each group of mice within an IntelliCage to establish an idiosyncratic social setting which ought to be taken into account in comparison of overt behaviors between treatments or genotypes, even in the case of inbred mice that can show minor yet stable differences in neuroanatomy and behavior. For this reason, when possible, housing of mice in IntelliCage should feature mixed groups (experimental and control mice being housed together in the same IntelliCage). However, conditioning tasks are carried out individually by each mouse within the corners, quite independently of social status and interactions. Hence, we consider conditioning success amenable for taking individual mice as independent data points in analyses. If similar statistically significant differences between treatment groups can be observed in two or more IntelliCages at the same location, we expect them to be robust even in case of possible environmental differences between cages. Should there be unusual differences between single cages (e.g., Kiryk et al., 2011), digital graphical replay of critical experiments may identify specific environmental conditions or activity patterns within cages (see also the legend of Figure 5).

### 2.9 Combining IntelliCages with add-ons

**Figure 11:**
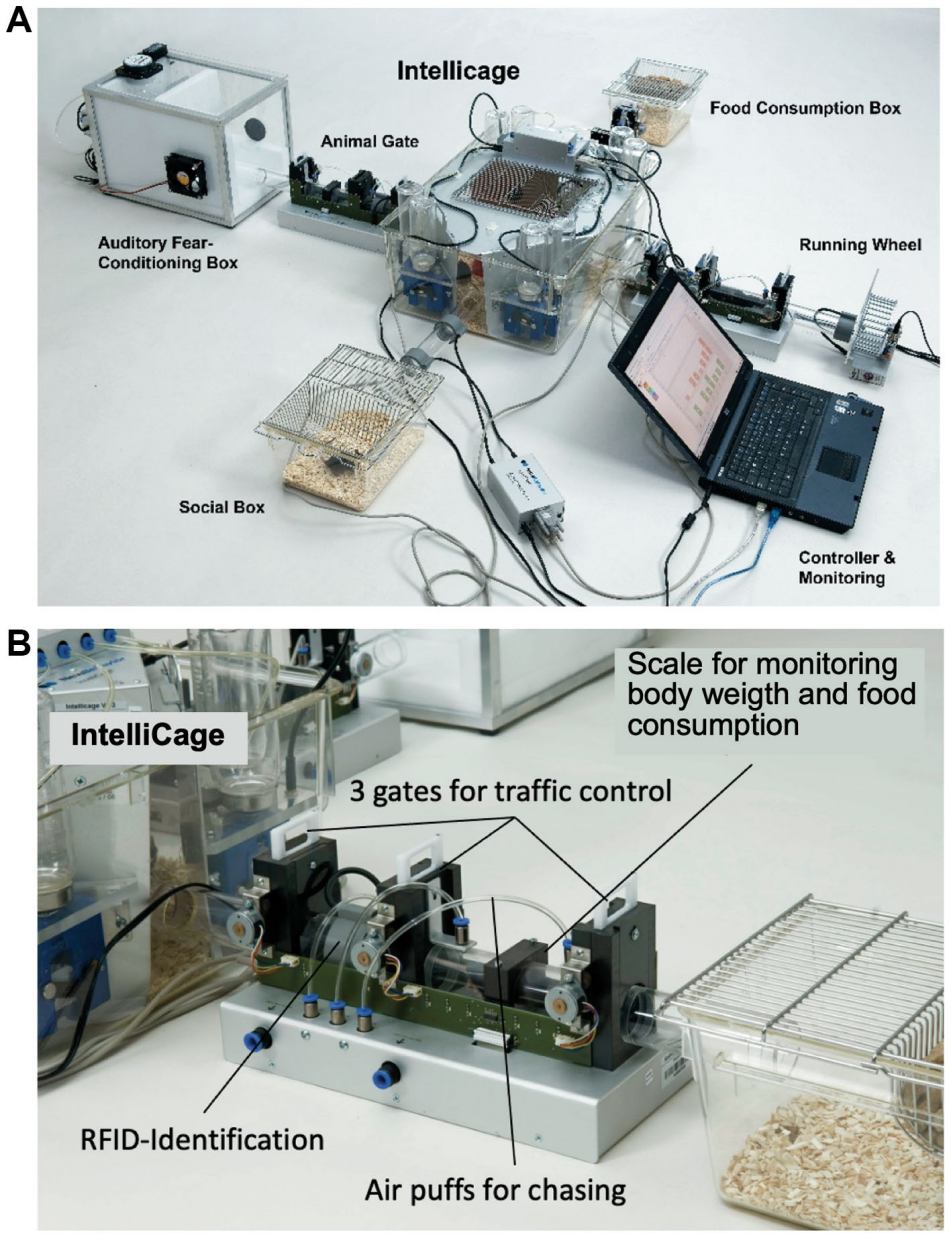
IntelliCage add-ons that can be controlled by the IntelliCage system. **(A)** Overview of all add-ons constructed under the FP6 programs “Intellimaze” and “Noveltune.” Picture provided by the University of Zürich. **(B)** Animal gate permitting or denying access to different test systems. The gate contains three doors regulating to-and-from traffic to external devices, supported by air-puffs driving away dawdling mice. The first compartment contains the RFID reader. For a figure showing an outside home cage connected to the IntelliCage for auditory testing, see Kahnau et al. (2023a,b). The add-on most successfully used up-to-now is the audiobox.

Supported by FP6 grants of the European Union, the projects Intellimaze and Noveltune were launched for expanding the home-cage concept by developing add-ons that can be connected to IntelliCages. The primary goal was to implement behavioral tests that were difficult to conduct in standard IntelliCages. Figure 11A shows a layout of the realized add-ons. The center piece is an IntelliCage whose software (designer, controller and analyzer) can also control communication with add-ons (Figure 11A). Mice can reach a so-called *social box* through tubes permitting RFID identification of mice passing inside, as well as their direction, and in this *social box* mice can find other mice or deposited scents or pheromones (Dere et al., 2018; Pan et al., 2019). Two social boxes permit to establish preference for socially relevant smells, analogously to the more complex RFID-based ECO-HAB system, which permits circulation of mice without a central home-cage (Puścian et al., 2016). If performance in a certain device should not be disturbed by partners, an *animal gate* can regulate access by blocking and opening the passage (Figure 11B). The animal gate also contains an inbuilt scale for monitoring the weight of mice, an opportunity useful to observe the consumption of different diets in attached cages, or to assess the impact of experimental manipulations or social stress. Because IntelliCage was not suited for presenting auditory cues, the consortium developed an *audiobox* which uses an IntelliCage corner placed at some distance in a sound-attenuated box and permits the use of IntelliCage software for self-paced auditory conditioning, resulting in publications that appeared even in particularly high-ranking journals (de Hoz and Nelken, 2014; Atlan et al., 2018; de Hoz et al., 2018; Chen et al., 2019; Chen and de Hoz, 2023). One may note that Kahnau et al. (2023a,b) used a reverse approach by keeping the mice in a home-cage connected to an empty IntelliCage from where the mice had temporary access to solitary auditory conditioning as controlled by an animal gate. Most of the add-ons are available from TSE-Systems, while another add-on is a floor plate made of RFID antennae described later under “Tracking of mice on the floor of IntelliCage” (Section 2.10.2).

### 2.10 Current and future modifications of IntelliCage

#### 2.10.1 Minor modifications

There have been several smaller or larger modifications that can be technically integrated in the IntelliCage system. Their usefulness depends on whether the intended use of an IntelliCage is testing mouse models under identical conditions, in which case modifications may jeopardize long-term comparability and reproducibility, or even patent applications. But when the goal is to tackle specific behavioral problems in an efficient and animal-friendly manner, modifications can be useful.

For experimenters wanting to avoid cage patrolling and social interaction between animals, it is possible to use a transparent *cage divider* that leaves 4 compartments with one corner, which is basically a two-faced operant conditioning box. It is slightly less animal-friendly because mice lack full contact with conspecifics, but mice are nonetheless not totally isolated as in individual home-cage testing systems, as they are able to see the other mice beyond the transparent dividers. Such dividers can also be useful when testing incompatible males.

The standard tubular RFID antenna poses a problem when studying *obese mice*. As observed in a variety of mouse models (Lutz and Woods, 2012), many obese mice will be unable to squeeze through the standard tube. Occasionally, small ramps may help to facilitate corner entry, but increasing obesity needs RFID readers with enlarged diameter as offered by TSE-Systems. Nevertheless, these larger RFID readers present some limitations when housing control and obese mice together, because two control mice may enter the compartment. This may lead to undesirable interactions during conditioning. A workaround solution is to mount two large RFID readers and keep two normal ones. This entails some limitations in the precise assessment of cage patrolling, but many intra-corner protocols will work.

Another modification under development is the modification of the signaling LED by a *touchscreen panel*. Presently, the LEDs include three programmable light sources, one on each side of the corner. Integrating a small touchscreen would permit to emulate the old LED arrangement yet could also present more complex visual schemes for discrimination learning. LCD (or LED) screens allow the presentation of simple as well as complex visual stimuli and have been extensively used in touchscreen and virtual reality experiments with rodents (Lopatina et al., 2020; Palmer et al., 2021). Reward consumption or refusal would still be possible as before by nose-poking because most of the touchscreen tasks developed benefit much more from the flexibility of the stimulus presentation than touching the correct position on a screen as a response element (Sullivan et al., 2021).

#### 2.10.2 Tracking of mice across the floor of IntelliCage

An often-requested add-on is the ability to follow the trajectories of the mice across the cage floor, although it has been shown that the locomotor activity of mice can be measured indirectly using the number of visits and by calculating which mouse follows corner visits of a cagemate. Typical findings such as circadian activity, habituation or age-dependent decline can be observed using this indirect measurement of locomotor activity (Codita et al., 2010; Krackow et al., 2010). Data generated with the PhenoCube, a modulated IntelliCage combined with a video tracking system, indicated a good correlation between general activity and corner visits in two Huntington’s disease models (Oakeshott et al., 2012; Balci et al., 2013).

**Figure 12:**
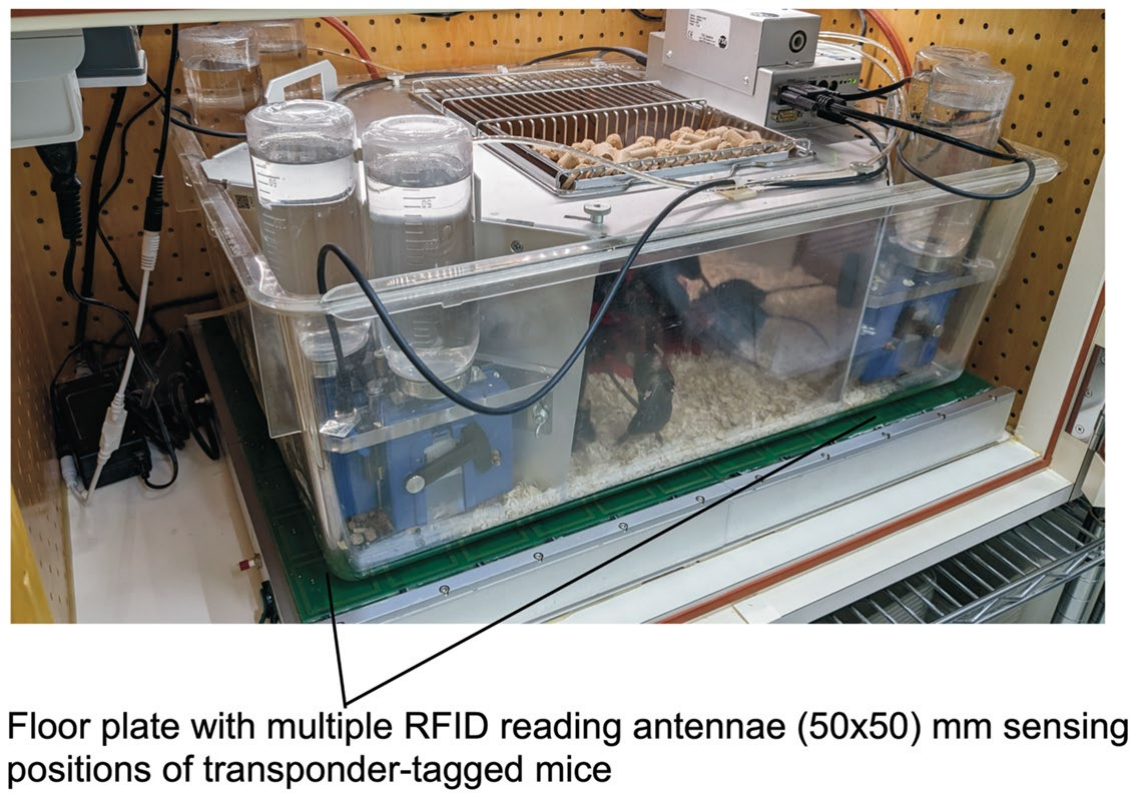
RFID floor plate (Phenovance) fitting exactly under the IntelliCage, comprising 50 × 50 mm antennas capable of recording proximity and trajectories of moving mice even when multiple mice are on the same antenna. Although the RFID floor plate requires a different type of transponder from that used in IntelliCage, these two types of transponders do not interfere with each other. Picture courtesy by Phenovance.

On the other hand, there is a broad interest to monitor and understand the social interactions, hierarchy, and general home-cage activity in the center part of the IntelliCage. A relatively simple solution is to place RFID-antennae under the IntelliCage so that the positions of various mice can be recorded (for figures see Klein et al., 2022). Such systems are available commercially, e.g., from TSE-Systems as Trafficage, which has an intentionally low spatial resolution^3^, or from Phenovance, which offers a higher resolution by using 5 × 5 cm RFID antenna tiles fitting under an IntelliCage (Figure 12). Transponders will be available that record both the floor activity and the corner visits of mice in an IntelliCage. This will permit to evaluate the individual distances between mice in the IntelliCage, as well as the speed and the trajectory of movements, yet it will miss fine interactions between animals. Nevertheless, the amount of data being analyzed can still be managed by ordinary laptop computers, making such extensions affordable. In our view, this new system could be most useful to estimate the amount of activity in an IntelliCage generated by social interactions.

However, to assess a detailed interaction between two or more mice, the only solution are video systems tracking head, tail and body direction of one or several mice. Earlier attempts to check the animals by video were limited to mounting small cameras observing the interior of the cage but being unsuitable for quantitative tracking (Mifflin et al., 2021; Winslow et al., 2021). The analysis of automated movement tracking in mice has made much progress in the last years (Peleh et al., 2019) and video tracking of a single mouse can resolve extremely fine movement variants of spontaneous behavior (Markowitz et al., 2018, 2023). However, the challenges of video tracking multiple mice without visible tags both in dark and under light in a cage where mice have places in which they can hide is still enormous. Typically, a combination of RFID antenna tiles and multiple cameras is necessary to recalibrate individual tracking (de Chaumont et al., 2019). Nonetheless, once achieved an effective motion tracking system, there will be newly developed software packages dealing with the immense data sets recorded, chiefly based on artificial intelligence (AI) including machine learning, such as DeepLabCut which enables body point estimation and tracking of individual animals housed singly or in a group (Mathis et al., 2018; Nath et al., 2019). There is an increasing number of AI-based technologies available to track and further analyse fine body movements according to trained classifiers for mouse behavior which are objective and independent of human definitions (Nilsson et al., 2020; Dunn et al., 2021; Fong et al., 2023; Sakamoto et al., 2023). Thus, one may expect further support by video techniques for the fine-grained analysis of mouse behavior in an IntelliCage, but the amount of data analysis associated with it (the so-called data footprint) will require careful consideration of specific experimental questions to be asked.

#### 2.10.3 Olfactory testing?

An interesting extension would be the possibility to train mice for olfactory discrimination in the IntelliCage. Volatile chemicals characteristic for the smell of food, of a predator or of a receptive female in the environment provide ethologically important information to many animals and are particularly important for mice and other rodents. The recent coronavirus (COVID-19) pandemic with the loss of smell as a typical symptom, and olfactory deficiencies in early stages of Alzheimer’s disease raised awareness of this sensory modality in humans. Olfactory assessment might be of special interest to many groups phenotyping Alzheimer mouse (AD) models, as olfactory dysfunction is considered as a pre-cognitive biomarker of AD. Furthermore, olfactory dysfunction is associated with several animal models of neurodevelopmental disorders, such as autism spectrum disorders, which have been intensively characterized in the IntelliCage (Puścian et al., 2014) and other devices (Lyons-Warren et al., 2021).

Currently, our understanding of how animals learn to use this information in the home-cage is limited, mainly due to the relatively challenging experimental settings required (Reinert et al., 2019; Reinert and Fukunaga, 2022). Presently, olfactory stimuli can be placed on top of the corner compartments of an IntelliCage to check olfactory novelty, but finer assessment is not possible. A solution without much modification would be to use animal gates to connect the IntelliCage to testing arenas analysing olfactory signals by ethological approaches such as the ECO-HAB system (Puścian et al., 2016), or to more complex test batteries as described by Zhang et al. (2022). Caglayan et al. (2021) used an RFID based animal sorter to link a home-cage with an external olfactory operant conditioning tool. Similarly, the AutonoMouse system (Erskine et al., 2019; Reinert et al., 2019; Ackels et al., 2021) allows RFID-based olfactory phenotyping of group-housed mice within the same apparatus.

As the IntelliCage is equipped with air-blowers, the available tubing and valve system might be modified for determining olfactory acuity just in front of the nose-poke holes, and by adding a ventilating system preventing spread of the olfactory traces in the IntelliCage. The concept is not new and has been presented before in a self-constructed arrangement (Kudryavitskaya et al., 2020). Thus, providing a defined air source in the IntelliCage corner combined with an olfactometer might allow to use the IntelliCage for olfactory phenotyping. Several behavioral tests in the categories of odor recognition, odor discrimination, and even episodic and emotional memory have been described in mice (Zhang et al., 2022) and could be transferred to the IntelliCage.

### 2.11 Adapting the IntelliCage to larger species

#### 2.11.1 Rat IntelliCage

**Figure 13:**
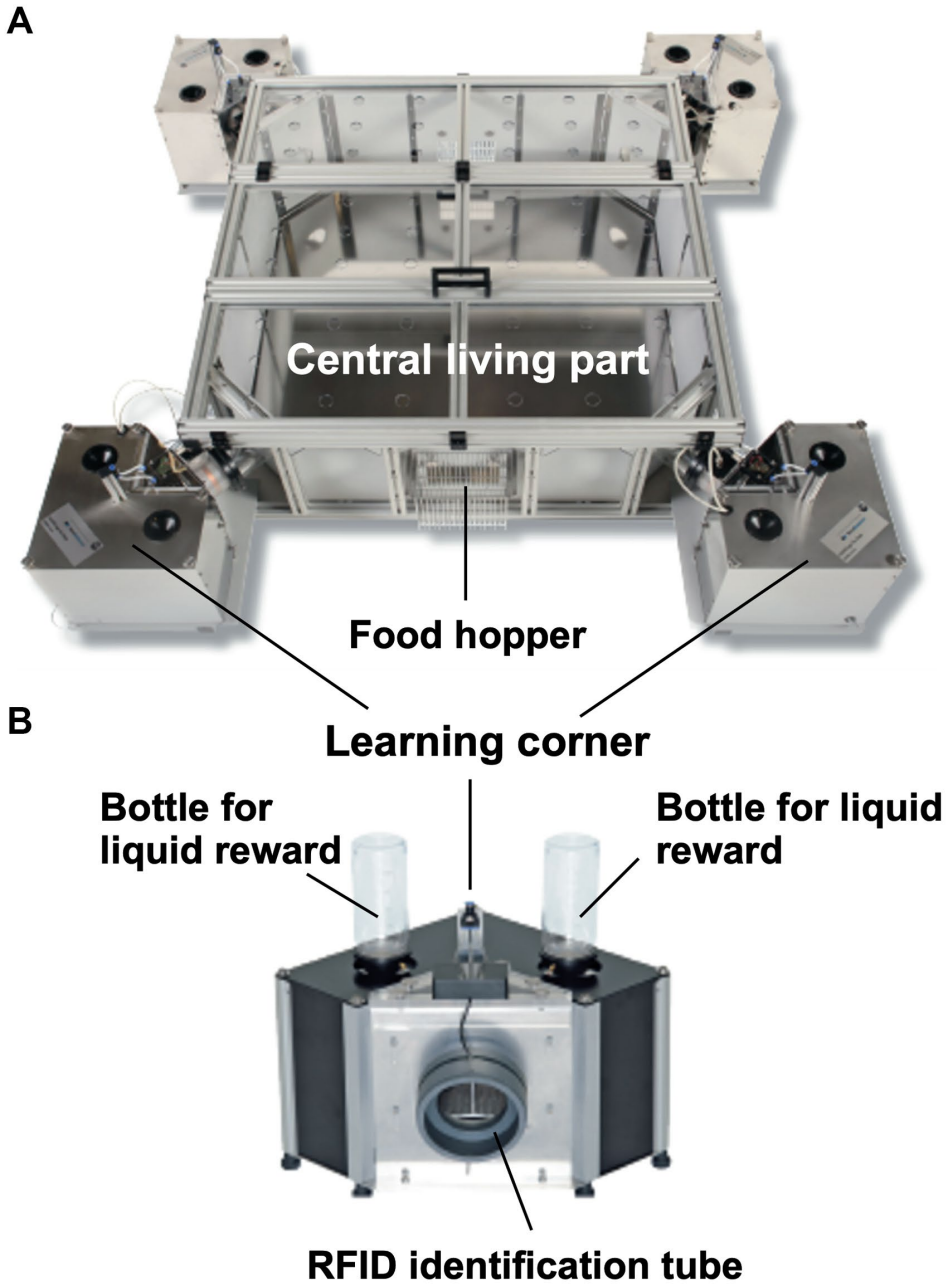
IntelliCage for rats. **(A)** Larger housing space required adaption of the IntelliCage system, and **(B)** adaptation of the corner system. The software of the mouse IntelliCage runs also with the rat system. Picture provided by TSE-Systems International.

The first prototypes of rat IntelliCages were produced in 2006 but needed some adaptation to rat behavior to make the systems run satisfactorily. The first adaptation was size, because rats need more space than offered by commercial rat cages (Figure 13A). The second adaptation included larger corners and larger tubular RFID-antennas (Figure 13B). However, the IntelliCage software could be used to run this system without modifications, because the inputs (presence, nose-poke, licks) as well as the outputs (opening/moving doors, activating LED and delivering air-puffs) were the same, and so the adaptation work was chiefly mechanical and permitted rapid use of the rat systems. Nonetheless, it took some years until the first rat paper appeared (Urbach et al., 2014), but then others followed, 13 over the past 5 years, proving that the mouse IntelliCage system could be adapted successfully to larger rodent species (Yang et al., 2017; Oliveros et al., 2018; Pelsőczi et al., 2020; Zheng et al., 2020; Cao et al., 2021; Esmaeili et al., 2022; Pham et al., 2022; Shishelova et al., 2022; Xiao et al., 2022; Li et al., 2023a,b; Pupikina and Sitnikova, 2023; Wu et al., 2023).

#### 2.11.2 Marmoset IntelliCage

**Figure 14:**
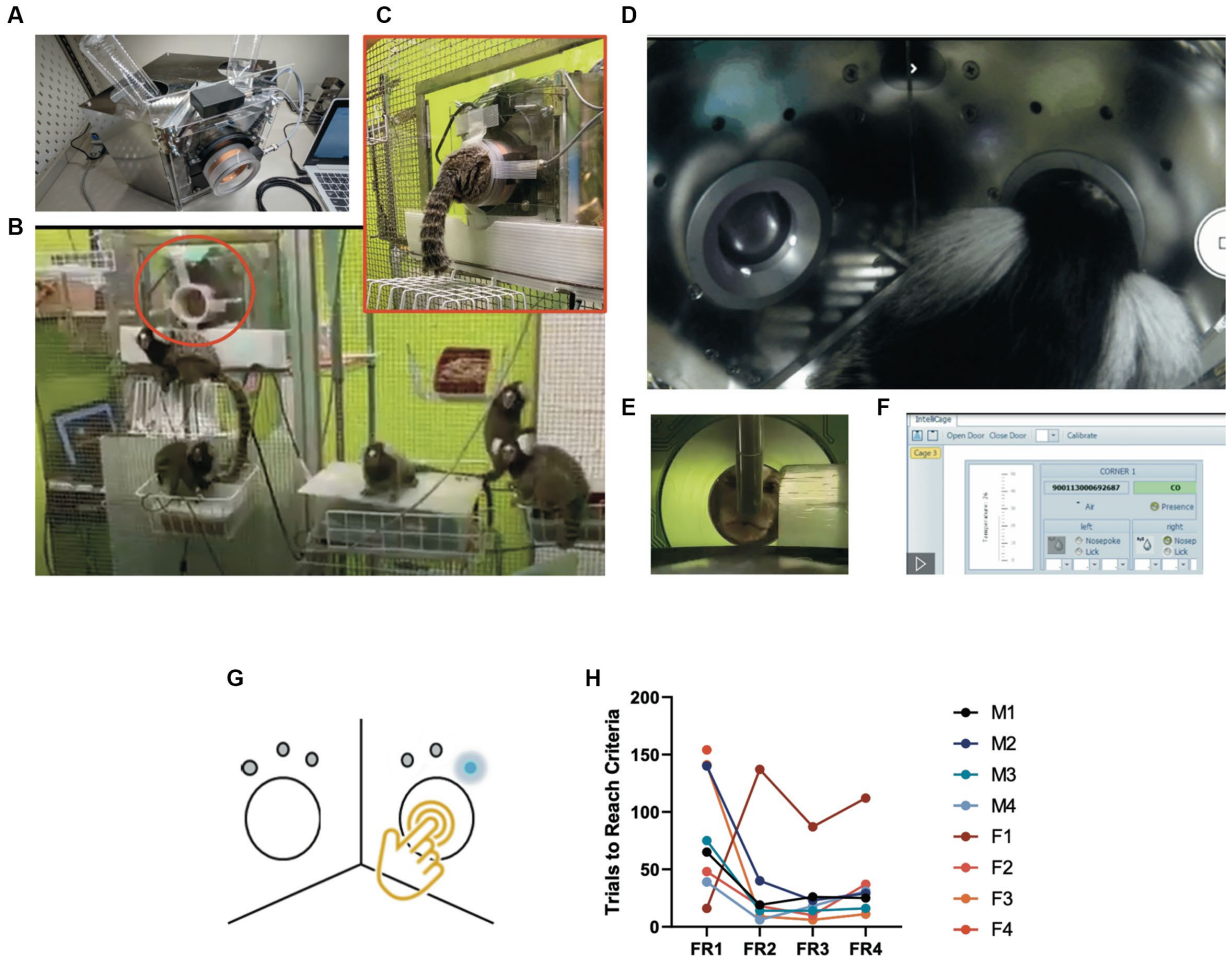
IntelliCage system for marmosets (*Callithrix jacchus*) as constructed by Seico Benner and Toshihiro Endo. **(A)** Modified rat corner. **(B)** Animal-friendly marmoset housing at the Yamasue Laboratory, Department of Psychiatry, Hamamatsu University School of Medicine (Hamamatsu, Japan). The position of a rat corner is marked by a red circle. Other corners can be placed everywhere, provided cabling and backside of the corner are protected. Mounting at the wall is the easiest solution. **(C)** Marmoset entering the tubular RFID identifier, which blocks access to others while one subject is working inside. **(D)** Inside view of the conditioning corner corresponding to the arrangement in Figure 4G. **(E)** Outside view of a corner, with the operating gate blocking access to the nipples. **(F)** Screenshot of the standard IntelliCage controller during training of monkeys showing activated signals for presence and nose-poke. **(G)** Set-up of a simple fixed-ratio (FR) discrimination learning task during which the monkey has to nose-poke or to touch the light barrier in front of the closed barrier several times to open the barrier. The position of the rewarded site is signaled by LEDs. The programming was done with the designer program. **(H)** Proof-of-principle pilot study demonstrating differential learning by 8 marmosets under fixed ratios (FR1–FR4, the latter indicating that 4 pokes/touches are required for gate opening). Data published by Benner (2022). Videos showing the various actions are provided in Supplementary Videos S1, S2.

Marmosets (*Callithrix jacchus*) have become increasingly popular in behavioral neuroscience, partly because they have an easily recognizable behavioral repertoire (Lipp and Hunsperger, 1978), partly because the Japanese government has launched a massive initiative with the goal of implementing marmosets as a primate alternative to rodents, including transgenic models (Okano et al., 2016). This has resulted in many institutions keeping marmosets in Japan, but the actual legal standards in animal welfare would preclude publications of their data in most Western journals. Thus, it appeared attractive to implement a large and animal-friendly home-cage system for these small primates. Because marmosets have a long tongue, they can easily reach the nipple of drinking bottles through openings in a rat IntelliCage corner (Figure 14A). At the Yamasue Laboratory of Hamamatsu University (Japan), to emulate an IntelliCage-like environment, corners were placed in a room housing several marmosets (Figures 14B,C).

The marmosets adapted quickly to the test situation (Figures 14D,E). Experimental protocols were assembled by the normal designer application and the controller used the same interface and analytics online (Figure 14F and Supplementary Videos S1, S2). Simple fixed ratio protocols for nose-poking were easily learned by most monkeys, even though marmoset groups often contain strong-willed individuals that do not like to be conditioned. However, these individual personality differences could make them even better models for translational research in neuroscience and neuropsychiatry (Figures 14G,H). The only restriction for set-ups of IntelliCage-like systems in large rooms is that a computer running the normal IntelliCage controller program can only handle 4 corners per location. Thus, for placing more than 4 test corners in a room, the IntelliCage system needs a program variant, IntelliCage StaR (available from Neurospex GmbH and XBehavior GmbH, Bänk, Switzerland) that avoids this restriction.

### 2.12 Classifying home-cage behavioral phenotypes by machine learning algorithms

Behavioral phenotypes are usually quantified by an arbitrary array of activity measures per subject during voluntary behavioral expression, e.g., Voikar et al. (2018), as well as by focusing on endpoints defined by the experimenter as responses to a sequence of conditioning tasks, e.g., Fischer et al. (2017), Voikar et al. (2018), and Volkmann et al. (2021). Many outcomes of conventional behavioral studies have proven to be highly inconsistent between experiments, which has often been attributed to strain, animal keeping, handling, lab maintenance, staff attitude, or environmental differences (Crabbe and Wahlsten, 2003; Wahlsten et al., 2003; Codita et al., 2012). Experimental reproducibility also suffers from the absence of an agreed-upon robust method to categorize and summarize behavioral phenotypes. Approaches in IntelliCage range from attempting to extract the most concise number of measures in order to reflect individual spatiotemporal activity patterns (Krackow et al., 2010) to calculating arbitrarily many (linear) combinations of measured variables allowing the algorithm to pick up any classifying patterns that might be hidden in the data (van Dijk et al., 2016, 2019).

In many areas of behavioral phenotyping research, the ultimate goal is to translate some intervention effect into changes of the subjects’ condition, e.g., assess whether a drug applied to knockout mice showing a “depression-like” phenotype restores “normal” behavior. To this end, the actual nature of the underlying behavioral variables (and their interactions) is not of relevance, but rather their operational value, i.e., whether a subject changes its phenotype in an expected or intended direction. Traditionally, some kind of “biomarker” serves as a proxy to indicate intervention effectiveness in translational research, e.g., Branchi et al. (2010). Hence, trained classifiers that could predict a subject’s phenotype might significantly short-cut such translational research on effectiveness of interventions: instead of first searching for a reliable “biomarker,” one might only have to check if the behavioral phenotype returns to the “normal” class after intervention. This is in fact the rationale of the approach for drug testing as used by PsychoGenics Inc. (Paramus, New Jersey) in their phenotyping tools SmartCube, NeuroCube and PhenoCube, the latter being a modified IntelliCage (Alexandrov et al., 2015).

To explore the potential of learning algorithms like convolutional neural networks (CNN) for extracting patterns that could be used to predict classes and, hence, in the future, to evaluate interference effects, a random forest model was trained to separate behavioral phenotypes of control and two lesioned groups of C57BL/6 J female mice using data from Voikar et al. (2018). Trained classifiers would have the advantage. Over classical models like canonical discriminant analysis. of not ultimately assume linearity (and monotonicity), yet also allowing for predictive classification of unknown samples.

**Figure 15:**
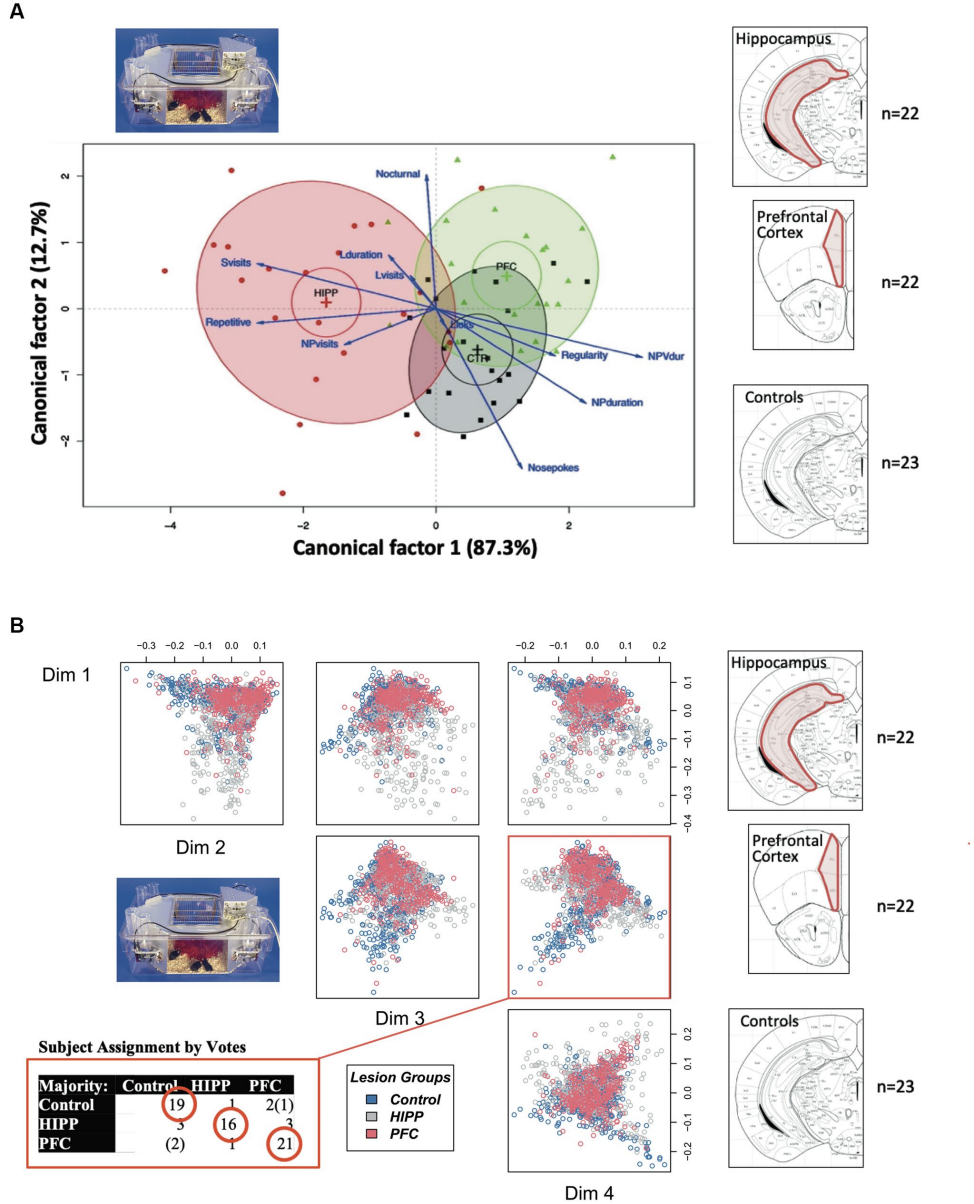
Comparison of conventional (linear) multivariate statistics and machine learning. **(A)** Standard canonical discrimination analysis of mouse learning in the IntelliCage showing decent separation of hippocampally lesioned mice (HIPP) from controls in the same cage, but considerable overlap between controls and prefrontal lesions (PFC). Data from Voikar et al. (2018). **(B)** Random forest analysis: same data analyzed by the random forest algorithm, which eventually could separate the three groups much better. Data presented by Krackow and Lipp (2023).

Figure 15 compares the outcome of a canonical discriminant analysis (which ultimately represents a linear multivariate variance model) with a typical classification procedure used in machine learning (random forest algorithm). Both methods classify subjects, according to their behavioral responses during non-conditioning periods within the long-term experimental sequence, into type-of-lesion groups (hippocampal, prefrontal and controls). Canonical discriminant analysis could not clearly differentiate between control and prefrontally lesioned mice, despite of the expected impairment in the latter (Figure 15A). The preliminary random forest evaluation given in Figure 15B appeared to separate the three groups of mice in the test sample quite well, possibly due to the basically non-linear approach. This is a most promising finding for the feasibility and profitability of AI and machine learning in behavioral phenotyping approaches in the translational realm. Clearly, the standardized way of stimulus presentation and response assessment in IntelliCage during the last 20 years system offers a unique opportunity for comparative retrospective analyses of data sets by means of AI.

## 3 Conclusion

- Among the many methods for behavioral testing of mice, the IntelliCage is certainly one of the most animal-friendly systems, as (1) it reduces interference with human handling, (2) it avoids testing in possibly anxiogenic environments external to the home-cage, (3) it provides social housing enriched by cognitive testing, and (4) it permits a wide range of patrolling-based and classical operant conditioning protocols (including gustatory and visual signal discrimination) without the employment of painful motivators such as electrical shocks. Even recording simple spontaneous activity is of high heuristic value.
- IntelliCage is also user-friendly, because it reduces the workload of experimenters associated with standard testing of mice, facilitates data analysis, and allows more time for reflection and planning, while caretakers profit from easy handling and cleaning.
- The mouse IntelliCage has a proven record for long-term sustainable standardization and reproducibility for behavioral testing, drug discovery, translational research, toxicology, and neuroscience, as reflected in its increasing use for patent application. Because of its simple design, the same hardware, software, and even similar protocols can be used for comparative testing of rodents and small primates. Thus, it has set and can set comparative standards for the future.
- IntelliCage systems can be expanded and modified for extracting more behavioral information if required, but at the cost of losing standardization and reproducibility.
- Within the field of behavioral testing, the IntelliCage is just one, yet efficient, tool and its output data may require to be checked by manual experiments.
- Despite IntelliCage’s friendliness for users, these should carefully consider the possibilities, but also the limitations, in using this tool. High throughput behavioral phenotyping without clear concepts may entail misleading results – the *festina lente* concept should prevail whenever applicable.
- IntelliCage output can be classified according to different views from ethology, experimental animal psychology and even artificial intelligence. Biased by ethological thinking, we believe that the IntelliCage is emulating a small world in which little cohorts of mice can move, explore, and solve problems by adjusting their ongoing activity to tasks presented by a computer, thus demonstrating flexibility at various levels of their brains, that were adapted evolutionarily to small worlds as well. Therefore, we expect or hope that the IntelliCage can help deciphering the enigma of behavioral flexibility and adaptability that mice share with humans.

## Author contributions

H-PL: Conceptualization, Data curation, Project administration, Visualization, Writing – original draft, Resources, Writing – review & editing. SK: Software, Visualization, Writing – review & editing, Writing – original draft. ET: Data curation, Visualization, Writing – review & editing. SB: Conceptualization, Methodology, Visualization, Writing – review & editing. TE: Conceptualization, Methodology, Visualization, Writing – review & editing. HR: Visualization, Writing – original draft, Writing – review & editing.

## Footnotes

^1^http://www.xbehavior.com/packages/intellicage

^2^See http://www.xbehavior.com/packages/intelliCage/.

^3^http://www.tse-systems.com/service/trafficage
