# Supplementary material for: IntelliCage: the development and perspectives of a mouse- and user-friendly automated behavioral test system
Source: Front Behav Neurosci. 2024 Jan 3;17:1270538. doi: 10.3389/fnbeh.2023.1270538 (PMC10793385; doi:10.3389/fnbeh.2023.1270538)
Supplement: Supplementary file 1 [file Table_1.PDF]

Behavioral parameter	Procedure	Reference (first author only)
ACTIVITY	**Free exploration**	Berry 2012; Branchi 2013; Cathomas 2015a 2015b; Codita 2012 2010; Endo 2012; Ermakova 2011; Fischer 2017; Gapp 2014; Gumucio 2013; Heidari 2016; Heinla 2018; Ishii 2015; Ismail 2016; Jedynak 2012; Jensen 2015; Kalm 2013; Kiryk 2008; Kobayashi 2013; Konopka 2010; Krackow 2010; Kulesskaya 2014 2013; Lan 2011; Lee 2015; Macpherson 2016; Masuda 2016; Mechan 2009; Mijakowska 2017 2015; Milior 2015; Muthuraju 2012 2013; Nowak 2013; Ogi 2015 2013; Onishchenko 2007; Osman 2014; Patrikainen 2014; Pelsoczi and Levay 2017; Raab 2017; Radwanska and Kaczmarek 2012; Ramakers 2012; Robinson and Riedel 2014; Roughton 2012; Rudenko 2009; Ryan 2013; Schuler 2012; Simmons 2016; Too 2016a 2016b 2016c 2016d; Ujita 2017; vanDijk 2015; Vannoni 2014; Viosca 2009; Voikar 2017 2010; Xia 2015; Yang 2016; Zheng 2018; Zhu 2010; Liu 2020; Liu 2020; Kimura 2020; Zheng 2020; Vogel 2020; Cisbani 2021; Cao 2021; Mifflin 2021; Yang 2022; Kandadai 2021; Winslow 2021; Volkmann 2021; Hahnefeld 2022; Wu 2023; Yesiltepe 2022; Gundersen 2023; Li 2023; Li,X 2023
	**Habituation**	Benner 2014; Berry 2012; Codita 2010; Gumucio 2013; Harda 2018; Heidari 2016; Holter 2015; Kobayashi 2013; Krackow 2010; Maroteaux 2017; Mechan 2009; Onishchenko 2007; Perez-Alcazar 2014; Ramakers 2012; Rudenko 2009; Ryan 2013; Too 2016d 2014a 2014c 2014d; vanDijk 2015; Voikar 2013; Weyer 2011; Konarzewski 2020; Kimura 2020; Zhang 2020; Nieraad 2020; Puścian 2020; Pagano 2022; Stephan 2022; Simon 2022; Schacke 2022; Winiarski 2022
	**Nosepoke adaptation**	Cathomas 2015a 2015b; Codita 2012; Ermakova 2011; Heidari 2016; Holter 2015; Ishii 2015; Ismail 2016; Kalm 2013; Krackow 2010; Kulesskaya 2014 2013; Lee 2015; Maroteaux 2017; Marwari and Dave 2018; Mechan 2009; Mijakowska 2015; Ogi 2015 2013; Raab 2017; Sano 2016; Simmons 2016; Too 2016a 2016b 2016c 2014a 2014b 2014c 2014d; Weyer 2011; Xia 2015; Yang 2016; Zhu 2010; Liu 2020; Liu 2020; Hühne 2020; Konarzewski 2020; Fischer 2020; Zhang 2020; Zheng 2020; Vogel 2020; Puścian 2020; Cisbani 2021; Yang 2022; Tran 2021; Kandadai 2021; Volkmann 2021; Hahnefeld 2022; Wu 2023; Pagano 2022; Yesiltepe 2022; Ojanen 2023; Gundersen 2023; Niiranen 2023; Vogel 2023; Wu 2023
	**Long-term home cage activity**	Rudenko 2009; Yamamoto 2023
	**Circadian activity**	Cathomas 2015 2017; Hardt 2019; Heinla 2018; Kulesskaya 2014; Marwari and Dave 2018; Mohammadi 2017; Piechota 2012; Too 2016c, 2016d; Ujita 2017; Fischer 2020; Hühne-Landgraf 2023; Yamamoto 2023
EMOTIONALITY	**Reaction to new environment**	Ben Abdallah 2013; Galsworthy 2005; Krackow 2010; Kulesskaya 2013, 2014; Onishchenko 2007; Peltola 2016; Rudenko 2009; Simmons 2016; Ujita 2017; Voikar 2013; Rivero 2021
	**Novel object preference**	Codita 2010; Faizi 2011; Mechan 2009; Zhang 2020
	**Novel smell (neophobia)**	Codita 2010
	**Light response test**	Too 2014a,b,c,d; 2016a,b,c
	**Drinking session adaptation**	Codita 2010; Ishii 2015; Kobayashi 2013; Kulesskaya 2014; Lee 2015; Maroteaux 2017; Safi 2006; Too 2016a 2016b 2016c 2016d; Voikar 2017; Kandadai 2021; Schacke 2022: Ojanen 2023; Gundersen 2023
	**Place learning**	Albuquerque 2013; Barlind 2010; Ben Abdallah 2013; Berry 2012; Codita 2010 2012; Dere 2018; Ermakova 2011; Faizi 2011; Fischer 2017; Fuchs 2017; Galsworthy 2005; Gumucio 2013; Hardt 2019; Heidari 2016; Holter 2015; Huo 2012; Ismail 2016; Itawa 2014; Jaholkowski 2009; Kalm 2013 2016; Karlsson 2011; Kiryk 2008; Knapska 2013 2006; Kobayashi 2013; Konopka 2010; Koss 2016; Krackow 2010; Kulesskaya 2014 2013; Lan 2011; Lee 2015; Maroteaux 2017; Masuda 2016; Mechan 2009; Netrakanti 2015; Onishchenko 2007; Osman 2014; Pelsoczi and Levay 2017; Peltola 2016; Perez-Alcazar 2014; Puscian 2014; Raab 2017; Robinson 2014; Roughton 2012; Rudenko 2009; Ryan 2013; Sekiguchi 2011; Simmons 2016; Ujita 2018; Vazquez 2015; Voikar 2017; Vyssotski 2010; Weyer 2011; Xia 2015; Yang 2016; Zheng 2018; Zhu 2010; Garrett 2020; Hühne 2020; Konarzewski 2020; Fischer 2020; Oizumi 2020; Zheng 2020; Vogel 2020; Nieraad 2020; Puścian 2020; Caperuchipi 2021; Cały 2021; Rivero 2021; Winslow 2021; Volkmann 2021; Tikhonova 2021; Kraft 2021; Wilke 2021; Arinrad 2021; Goncerzewicz 2022; Stephan 2022; Schacke 2022; Yesiltepe 2022; Barth 2023; Li 2023; Li,X 2023; Gabaeva 2023; Niiranen 2023; Vogel 2023

LEARNING and MEMORY	Reversal place learning	Albuquerque 2013; Ben Abdallah 2013; Berry 2012; Codita 2012 2010; Dere 2018; Fischer 2017; Fuchs 2017; Galsworthy 2005; Gumucio 2013; Hardt 2019; Heidari 2016; Holter 2015; Huo 2012; Ismail 2016; Itawa 2014; Kalm 2013 2016; Karlsson 2011; Kobayashi 2013; Koss 2016; Krackow 2010; Kulesskaya 2014 2013; Lan 2011; Lee 2015; Maroteaux 2017; Masuda 2016; Mechan 2009; Netrakanti 2015; Onishchenko 2007; Osman 2014; Pelsoczi and Levay 2017; Peltola 2016; Perez-Alcazar 2014; Puscian 2014; Raab 2017; Robinson 2014; Roughton 2012; Rudenko 2009; Ryan 2013; Sekiguchi 2011; Simmons 2016; Ujita 2018; Vazquez 2015; Voikar 2017; Weyer 2011; Xia 2015; Yang 2016; Zhu 2010; Garrett 2020; Oizumi 2020; Horigane 2020; Zhang 2020; Vogel 2020; Nieraad 2020; Tran 2021; Winslow 2021; Wilke 2021; Arinrad 2021; Schacke 2022; Jetsonen 2023; Li 2023; Li,X 2023; Niiranen 2023; Radlicka 2023; Vogel 2023
	Serial Reversal place learning	Huo,2012; Kalm 2013; Karlsson 2011; Masuda 2018; Osman 2014; Roughton 2012; Mehr 2020; Horigane 2020; Volkmann 2021; Stephan 2022; Ojanen 2023
	Extinction of place preference	Codita 2010; Gumucio 2013; Vogel 2020; Gabaeva 2023
	Side learning/discrimination	Cathomas 2015a; Codita 2012; Knapska 2013; Marwari and Dave 2018; Osman 2014; Rudenko 2009; Voikar 2017; Vogel 2020
	Reversal Side learning	Codita 2012; Vogel 2020; Barth 2023
	Place avoidance	Albuquerque 2013; Codita 2010; de Hoz 2017; d'Isa 2011; Faizi 2011; Gumucio 2013; Itawa 2014; Jaholkowski 2009; Hardt 2019; Jensen 2015; Karlsson 2011; Kiryk 2008; Knapska 2013 2006; Kobayashi 2013; Marwari and Dave 2018; Masuda 2016; Mechan 2009; Nowak 2013; Rudenko 2009; Voikar 2017 2010; Fischer 2020; Mifflin 2021; Tran 2021; Cały 2021; Hahnefeld 2022; Barth 2023; Li 2023
	Reversal of place avoidance	Mechan 2009; Voikar 2017; Li 2023
	Extinction of place avoidance	d'Isa 2011; Hardt 2019; Itwawa 2014; Masuda 2016; Nowak 2013; Voikar 2010; Serykh 2020; Hahnefeld 2022
	Cued punishment test	Lan 2011; Yang 2022; Goncerzewicz 2022
	Sequencing task	Aung 2016; Benner 2014; Endo 2012 2011; Gapp 2014; Hardt 2019; Macpherson 2016; Marwari and Dave 2018; Sano 2016; Fischer 2020
	Patrolling (e.g.clockwise)	Albuquerque 2013; Fischer 2017; Holter 2015; Kobayashi 2013; Kulesskaya 2014; Onishchenko 2007; Peltola 2016; Rudenko 2009; Too 2016a 2016b 2016c 2016d; Vazquez 2015; Voikar 2017; Weyer 2011; Zheng 2018; Garrett 2020; Jetsonen 2023
	Patrolling reversal	Albuquerque 2013; Kobayashi 2013; Too 2016a 2016b 2016c 2016d; Voikar 2017
	Chaining	Fischer 2017; Kobayashi 2013
	Reaction time task / motor impulsivity	Fischer 2017; Kobayashi 2013; Masuda 2016 2018; vanDijk 2015; Serykh 2020; Morello 2020; Cisbani 2021; Esmaeili 2022; Jörimann 2023
	Delay discounting task	Gapp 2014; Hardt 2017; Masuda 2016, 2018; Harda 2020; Morello 2020; Meng 2021; Nakamura 2021; Rivero 2021; Gabaeva 2023
	DRL (differential reinforcement of lower rates) paradigm	Gapp 2014; Hardt 2019; Fischer 2017; Kobayashi 2013; vanDijk 2015
	Conditioned drinking suppression	Voikar 2010
	LED stimulus-dependent alternation	Voikar 2017; Tran 2021
SOCIAL BEHAVIORS	Group place learning	Galsworthy 2005; Harda 2018; Jaholkowski 2009; Kiryk 2011
	Competition task	Benner 2014; Endo 2012; Ishii 2015; Kulesskaya 2013; Ujita 2017 2018; Xing 2021
	Social interactions in alcohol drinking	Smutek 2014; Frycz 2023
	Social modulation of aversive memories	Nowak 2013
	Social stress	Branchi 2010, 2013; Bergamini 2016; Poggini, 2023
	Different tests for alcohol drinking	Holgate 2017; Koskela 2018; Mijakowska 2015, 2017; Parkitna 2013; Radwanska 2012; Smutek 2014; Stefaniuk 2017; Simmons 2021; Pagano 2022; Nalberczak-Skóra 2023; Stefaniuk 2023; Pagano 2023; Perschler, 2023
	Vogel water-lick paradigm adapted to IC	Fischer 2017; Safi 2006; vanDijk 2015

OTHER BEHAVIORS in PHARMACOLOGICAL TREATMENT	**Fixed/Progressive ratio (operant conditioning response)**	Jastrzebska 2016; Skupio 2017; Vazquez 2015; Hühne 2020; Wilke 2021
	**Chronic unpredictable stress/Acute stress**	Alboni 2015; Branchi 2013; Jensen 2015; Milior 2015; Zheng 2020; Picard 2021
	**Anhedonia / taste aversion**	Alboni 2015; Bergamini 2016; Branchi 2010, 2013; Dere 2018; Heinla 2018; Milior 2015; Mohammadi 2017; Ratner 2016; Chotard 2020; Poggini 2021, 2023
	**Sucrose/saccharine preference**	Pupikina 2023, Nagaeva 2023
	**Taste preference (liquid)**	Chotard 2020; Mohammadi 2017, 2023
	**Cocaine/Morphine self-administration**	Ajonijebu 2017; Skupio 2017
SENSORY TESTING (AUDIOBOX)	**Auditory distraction**	Atlan 2018
	**Auditory function**	Bahader 2021; Rankovic 2021
	**Frequency tuning**	deHoz 2014, 2018; Chen 2019
	**Perceptual categorization**	Chen 2023
