# Supplementary material for: IntelliCage: the development and perspectives of a mouse- and user-friendly automated behavioral test system
Source: Front Behav Neurosci. 2024 Jan 3;17:1270538. doi: 10.3389/fnbeh.2023.1270538 (PMC10793385; doi:10.3389/fnbeh.2023.1270538)
Supplement: Supplementary file 2 [file Image_1.PDF]

Suppl. Fig 1 Lipp et al.

Poster presented at FENS 2000 Brighton summarizing main results of feralization studies with L1 transgenic mice

Natural learning in mouse populations: food place reversal in mice overexpressing the cell adhesion molecule L1

A.L.Vyssotski[1,2*], G. Dell'Omo[1,3], D.L. Vyssotski[2], D.P. Wolfer[1], M.Schachner[4], I.I. Poletaeva[5], H.-P. Lipp[1],
[1]Inst. of Anatomy, Univ. of Zurich, CH-8057 Zurich, Switzerland; [2]P.K.Anokhin Institute of Normal Physiology, Russian Academy of Sciences, Moscow, Russia; [3]Laboratory of Veterinary Medicine, Instituto Superiore di Sanita, Rome, Italy; [4]Zentrum fur Moleculare Neurobiologie, Universitat Hamburg, FRG; [5]Dept. of Biology, Moscow State University.

МОСКОВСКИЙ ГОСУДАРСТВЕННЫЙ УНИВЕРСИТЕТ им. М. В. Ломоносова
119899, Москва, В-234, Ленинские горы

? Are the transgenic mice overexpressing the neural cell adhesion molecule L1 more flexible in natural environment?

Transgenic mice overexpressing the neural cell adhesion molecule L1 (under the control of the promoter for glial fibrillary acid protein, GFAP) are known for slightly superior flexibility during water maze learning (Eur. J. Neurosci 10: 708-717, 1998). In particular, they were found to adapt quicker to new situations such as platform reversal. The aim of the present study was to test behavioral flexibility under more naturalistic conditions.

1 How to test mouse learning abilities in natural environment

1. The outdoor pen 20 x 20 m isolating experimental population from wild neighbors with two shelters 2 x 2 m protecting mice against bad weather and predators was constracted in advance.

2. 8 squared antennas 35 x 35 cm for Trovan ID-100 microchip transponders reading were placed inside outdoor pen as it is shown and were connected to the computer for data downloading.

3. 78 mice (hetero- or homozygous transgenics: 27 males, 28 females; wildtypes 10 males, 13 females) were targeted with Trovan ID-100 transponders by subcutaneous injection under light anastesia and were released for 68 days into the pen.

4. The food position has been moved from antennas to other antennas several times in accordance with schema 4 (always at 18:00). The times of entries for each mouse into each antenna have been detected and recorded.

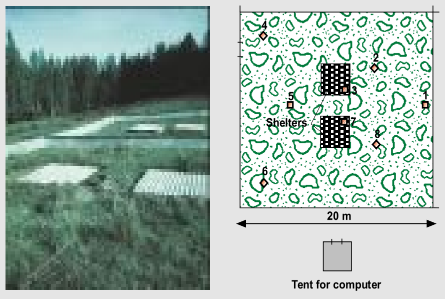

2 No difference in general activity between L1 and WT, circadian rhythmicity in transgenics is also normal

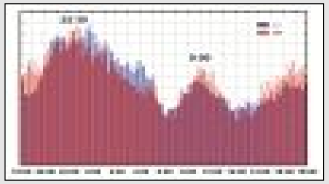

The numbers of total visits of antennas by wild type animals and transgenics during experiment coinside with high accuracy (difference less than 1%). Peaks of daily activity (9:00 and 22:30) also coinside.

3 Survival rates are equal after two months in outdoor pen

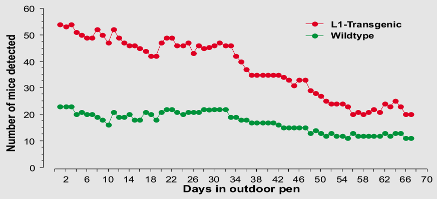

There was no difference in percentage of loss in 67 days of outdoor life between groups. Approximately one half of originally released group was lost in two months.

4 More visits of distant places during adaptation

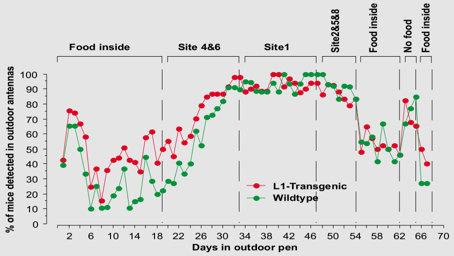

Transgenic aqnimals have demonstrated more visits of distant places during adaptation and did not differ from control animals afterwards.

5 Faster place reversal

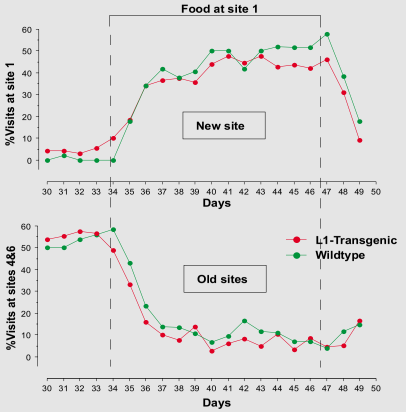

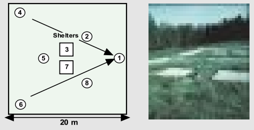

The analysis revealed that after stopping food delivery inside the shelters, the transgenic animals increased their searching activity significantly more than wildtype mice ($p < 0.01$). In addition, transgenic mutants returned to the previous food locations less frequently ($p<0.02$). The differences in percentages of animals visited food sites during 24 h after place reversals are also visible.

! Conclusions

Testing reversal learning under naturalistic conditions confirms the predictions based on water maze learning. On the other hand, analysis of short-term survival during the observation period shows no advantage for the transgenic mice, and they appear to be less able to survive Russian winters. Pending further confirmation of natural selection data, we conclude that the moderately superior flexibility of the L1 transgenics does not translate into obvious advantages during the daily life of the mice.

Further observations and experiments will show whether the behavioral changes themselves are maladaptive in the long run (e.g., increased risk of predation because of facilitated moving to new places) or whether the L1 transgene construct entails some hidden impairments affecting biological fitness.

Swiss National Science Foundation Grants 31-46691.96 and 31-57139.99

BIO4CT980297/BBW98.0125 and the German Scientific Research Foundation
