# Supplementary material for: IntelliCage: the development and perspectives of a mouse- and user-friendly automated behavioral test system
Source: Front Behav Neurosci. 2024 Jan 3;17:1270538. doi: 10.3389/fnbeh.2023.1270538 (PMC10793385; doi:10.3389/fnbeh.2023.1270538)
Supplement: Supplementary file 3 [file Image_2.PDF]

Dos and don’ts in the Intellicage

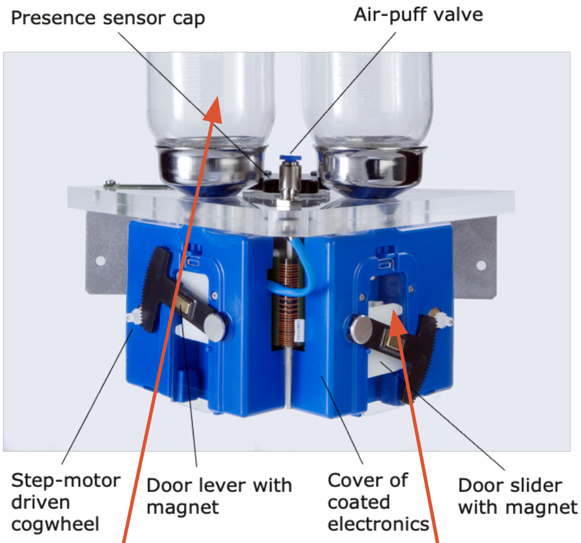

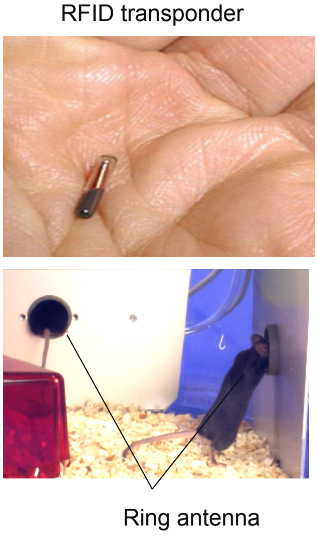

1) Make sure that water bottles stand perfectly upright and calibrate internal pressure

3) Make sure that transponders are aligned to the body axis of the mouse

2) Clean regularly surface containing switching magnets

Occasional problems when using Intellicage

Generally, the cages run well and have been used by many laboratories over long periods, but this requires regular maintenance. The most frequent problems are listed here in descending order:

1) Water bottles and caps are standard products for animal care and are thus not precision-made. Prior to any launch of an experiment, water bottles should be filled and checked for leaking (most frequent problem) or for withholding water when a mouse is licking. A practical solution is to construct a rack holding the bottles prior to an experiment and use only those with a hanging drop of water. Bottles should also stand in perfect vertical position, so caps must fit properly. Recent Intellicage models have a plastic frame holding the bottles in position. Marking the water position on the bottle permits checking whether the bottle withholds water.
2) The plastic gliders permitting or blocking access to the water nozzle contain small magnets in their upper plate that tell the controller that a door is closed. If the gliding channels and the surface are not regularly cleaned, the gliders do sometimes no longer reach their maximal vertical position so that the switches are not activated. Usually, the system tries to lower the gliders and repeating the closing a few times. For safety reasons, an ongoing experiment is then terminated leaving all doors open to permit access to water. The most frequent cause of the problem is lack of regular cleaning because users are shy to disassemble the corners even though it is not difficult.
3) The transponder must be aligned to the body axis of the animal. The magnetic field of the tubular ring-antenna activating a radio response from the chip works only if the coil in the glass transponder has the same orientation. Obliquely implanted transponders are poorly read. Possible precautions are:
   - having an RFID reader in tubular form, as mice can (or should be) transferred in short tubes.
   - always keeping one more mouse in the Intellicage cohort as statistically needed. Thus, animals whose transponders get lost or misplaced under the skin can be removed from a running experiment.
4) The food hopper must fit snugly in the cover. If not, mice can escape and will, if not detected, eventually destroy electronic cabling.

Preparing male or female mice for Intellicage testing according to Benner and Endo (2023)

These steps are not only for experiments with males but also for females.

Step 1: Prior to experiments, mice should be kept in groups of 5-6 mice, ideally littermates. After the microchips are implanted, wait for about one week, and check thereafter whether the microchips have not been lost. Loss of transponders after implantation may be minimized by a drop of tissue glue over the injection site. Correctly placed or obliquely misplaced transponders may be felt by gentle palpation of the back regions. After one day, ensure that the health of the mice has not been affected by the handling and implantation stress by checking for injuries to body surfaces, loss of fur, weight loss, etc. If there are no problems, proceed to step 2.

Step 2: Group together in advance the mouse cohort and pre-adapt these mice to the Intellicage and the cage-mates by placing them in an empty Intellicage (i.e., Tecniplast 2000P type cage, including mouse house). After 1 to 2 weeks, check each mouse again for injuries to its body surfaces, loss of fur, weight loss, etc. If there are no problems, proceed to Step 3. Omission of this phase will render the adaptation period useless for analysis of behavior as it will reflect a mix of situational and social novelty.

Step 3: Transfer the group of mice kept in the same cage in step 2 to a fully equipped Intellicage or place the cover plate with the learning corners on the actual cage to minimize olfactory novelty and start the Intellicage recording.

Other precautions

- To minimize as much as possible stress factors that may induce fighting or social unrest, try to use plastic tunnels or small cages without grabbing the tails of the mice when handling or moving them.
- Reduce the number of mice per Intellicage when running complex protocols that require prolonged presence in a conditioning corner
- Ideally, some pilot studies should be run with a spare Intellicage to check whether a specific protocol has no bugs preventing learning by the mice, to estimate the time needed to learn more complex tasks, and to recognize when an envisioned procedure exceeds the cognitive abilities of normal mice (and sometimes of humans).
- Whenever possible, add to the minimal statistically required number per Intellicage an additional mouse per treatment group and run the experiment with it. This allows to remove mice with displaced transponders or sick looking animals. Do not try to inject a second transponder into a mouse and avoid introducing a “replacement” mouse while an experiment is running as this will cause problems. Note such manipulations in the log file of the controller program and publish them in the method section of papers when such actions were needed.
- If a dominant male is identified (the one with no injuries or the one patrolling alone while the others huddle together) do not try to remove it. This triggers in most cases fighting for the new alpha position, generating social unrest and increased locomotion in the IntelliCage. Just note it in the comment section of the controller.
- When comparing males versus females in the Intellicage, do not keep the Intellicages in the same room. You may run a simultaneous experiment with the same system, just by placing the IntelliCages in adjacent rooms but use long cables for connecting them.
