# Supplementary material for: IntelliCage: the development and perspectives of a mouse- and user-friendly automated behavioral test system
Source: Front Behav Neurosci. 2024 Jan 3;17:1270538. doi: 10.3389/fnbeh.2023.1270538 (PMC10793385; doi:10.3389/fnbeh.2023.1270538)
Supplement: Supplementary file 4 [file Data_Sheet_1.PDF]

Supplementary References

All References for Intellicage 2005 – October 15 2023, alphabetical and chronological ordering

Contains a few references of review papers (marked yellow)

Alphabetical

Ajonijebu, D.C., Abboussi, O., Mabandla, M.V., and Daniels, W.M.U. (2018). Differential epigenetic changes in the hippocampus and prefrontal cortex of female mice that had free access to cocaine. *Metabolic Brain Disease* 33(2)**,** 411-420. doi: 10.1007/s11011-017-0116-z.

Ajonijebu, D.C., Abboussi, O., Mabandla, M.V., and Daniels, W.M.U. (2019). Cocaine-induced inheritable epigenetic marks may be altered by changing early postnatal fostering. *NeuroReport* 30(17)**,** 1157-1165. doi: 10.1097/WNR.0000000000001332.

Akbergenov, R., Duscha, S., Fritz, A.K., Juskeviciene, R., Oishi, N., Schmitt, K., et al. (2018). Mutant MRPS5 affects mitoribosomal accuracy and confers stress-related behavioral alterations. *EMBO reports* 19(11)**,** e46193. doi: 10.15252/embr.201846193.

Alboni, S., Poggini, S., Garofalo, S., Milior, G., El Hajj, H., Lecours, C., et al. (2016). Fluoxetine treatment affects the inflammatory response and microglial function according to the quality of the living environment. *Brain, Behavior, and Immunity* 58**,** 261-271. doi: 10.1016/j.bbi.2016.07.155.

Albuquerque, B., Häussler, A., Vannoni, E., Wolfer, D.P., and Tegeder, I. (2013). Learning and memory with neuropathic pain: impact of old age and progranulin deficiency. *Frontiers in Behavioral Neuroscience* 7. doi: 10.3389/fnbeh.2013.00174.

Alexandrov, V., Brunner, D., Hanania, T., and Leahy, E. (2015). High-throughtput analysis of behavior for drug discovery. *European Journal of Pharmacology* 753**,** 127-134. doi: 10.1016/j.ejphar.2015.02.037.

Alexandrov, V., Brunner, D., Menalled, L.B., Kudwa, A., Watson-Johnson, J., Mazzella, M., et al. (2016). Large-scale phenome analysis defines a behavioral signature for Huntington's disease genotype in mice. *Nature Biotechnology* 34(8)**,** 838-844. doi: 10.1038/nbt.3587.

Arinrad, S., Wilke, J.B.H., Seelbach, A., Doeren, J., Hindermann, M., Butt, U.J., et al. (2021). NMDAR1 autoantibodies amplify behavioral phenotypes of genetic white matter inflammation: a mild encephalitis model with neuropsychiatric relevance. *Molecular Psychiatry*. doi: 10.1038/s41380-021-01392-8.

Atlan, G., Terem, A., Peretz-Rivlin, N., Sehrawat, K., Gonzales, B.J., Pozner, G., et al. (2018). The Claustrum Supports Resilience to Distraction. *Current Biology* 28(17)**,** 2752-2762.e2757. doi: 10.1016/j.cub.2018.06.068.

Aung, K.H., Kyi-Tha-Thu, C., Sano, K., Nakamura, K., Tanoue, A., Nohara, K., et al. (2016). Prenatal Exposure to Arsenic Impairs Behavioral Flexibility and Cortical Structure in Mice. *Frontiers in Neuroscience* 10. doi: 10.3389/fnins.2016.00137.

Bahader, I. (2021). *Behavioral and electrophysiological assessment of hearing function in mice with deficient sound encoding at inner hair cell ribbon synapse.* MD, Georg-August-University.

Balan, S., Iwayama, Y., Ohnishi, T., Fukuda, M., Shirai, A., Yamada, A., et al. (2021). A loss-of-function variant in SUV39H2 identified in autism-spectrum disorder causes altered H3K9 trimethylation and dysregulation of protocadherin β-cluster genes in the developing brain. *Molecular Psychiatry*. doi: 10.1038/s41380-021-01199-7.

Balci, F., Shamy, J.L., El-Khodor, B.F., Filippov, I., Mushlin, R., Port, R., et al. (2013). High-Throughput Automated Phenotyping of Two Genetic Mouse Models of Huntington's Disease. *PLoS Currents*. doi: 10.1371/currents.hd.124aa0d16753f88215776fba102ceb29.

Barlind, A., Karlsson, N., Björk-Eriksson, T., Isgaard, J., and Blomgren, K. (2010). Decreased cytogenesis in the granule cell layer of the hippocampus and impaired place learning after irradiation of the young mouse brain evaluated using the IntelliCage platform. *Experimental Brain Research* 201(4)**,** 781-787. doi: 10.1007/s00221-009-2095-8.

Barranco, A., Garcia, L., Gruart, A., Delgado-Garcia, J.M., Rueda, R., and Ramirez, M. (2022). Effects of β-Hydroxy β-Methylbutyrate Supplementation on Working Memory and Hippocampal Long-Term Potentiation in Rodents. *Nutrients* 14(5)**,** 1090. doi: 10.3390/nu14051090.

Barth, K., Vasic, V., McDonald, B., Heinig, N., Wagner, M.C., Schumann, U., et al. (2023). EGFL7 loss correlates with increased VEGF-D expression, upregulating hippocampal adult neurogenesis and improving spatial learning and memory. *Cell Mol Life Sci* 80(2)**,** 54. doi: 10.1007/s00018-023-04685-z.

Baumann, P., Schriever, S.C., Kullmann, S., Zimprich, A., Peter, A., Gailus-Durner, V., et al. (2021). Diabetes type 2 risk gene Dusp8 is associated with altered sucrose reward behavior in mice and humans. *Brain Behav* 11(1)**,** e01928. doi: 10.1002/brb3.1928.

Ben Abdallah, N.M.B., Filipkowski, R.K., Pruschy, M., Jaholkowski, P., Winkler, J., Kaczmarek, L., et al. (2013). Impaired long-term memory retention: Common denominator for acutely or genetically reduced hippocampal neurogenesis in adult mice. *Behavioural Brain Research* 252**,** 275-286. doi: 10.1016/j.bbr.2013.05.034.

Ben-Simon, Y., Kaefer, K., Velicky, P., Csicsvari, J., Danzl, J.G., and Jonas, P. (2022). A direct excitatory projection from entorhinal layer 6b neurons to the hippocampus contributes to spatial coding and memory. *Nature Communications* 13(1)**,** 4826. doi: 10.1038/s41467-022-32559-8.

Benner, S., Endo, T., Endo, N., Kakeyama, M., and Tohyama, C. (2014). Early deprivation induces competitive subordinance in C57BL/6 male mice. *Physiology & Behavior* 137**,** 42-52. doi: 10.1016/j.physbeh.2014.06.018.

Benner, S., Endo, T., Kakeyama, M., and Tohyama, C. (2015). Environmental insults in early life and submissiveness later in life in mouse models. *Frontiers in Neuroscience* 9. doi: 10.3389/fnins.2015.00091.

Benraiss, A., Wang, S., Herrlinger, S., Li, X., Chandler-Militello, D., Mauceri, J., et al. (2016). Human glia can both induce and rescue aspects of disease phenotype in Huntington disease. *Nature Communications* 7(1). doi: 10.1038/ncomms11758.

Bergamini, G., Cathomas, F., Auer, S., Sigrist, H., Seifritz, E., Patterson, M., et al. (2016). Mouse psychosocial stress reduces motivation and cognitive function in operant reward tests: A model for reward pathology with effects of agomelatine. *European Neuropsychopharmacology* 26(9)**,** 1448-1464. doi: 10.1016/j.euroneuro.2016.06.009.

Beroun, A., Nalberczak-Skóra, M., Harda, Z., Piechota, M., Ziółkowska, M., Cały, A., et al. (2018). Generation of silent synapses in dentate gyrus correlates with development of alcohol addiction. *Neuropsychopharmacology* 43(10)**,** 1989-1999. doi: 10.1038/s41386-018-0119-4.

Berry, A., Amrein, I., Nötzli, S., Lazic, S.E., Bellisario, V., Giorgio, M., et al. (2012). Sustained hippocampal neurogenesis in females is amplified in P66Shc−/− mice: An animal model of healthy aging. *Hippocampus* 22(12)**,** 2249-2259. doi: 10.1002/hipo.22042.

Branchi, I., D'Andrea, I., Cirulli, F., Lipp, H.-P., and Alleva, E. (2010). Shaping brain development: Mouse communal nesting blunts adult neuroendocrine and behavioral response to social stress and modifies chronic antidepressant treatment outcome. *Psychoneuroendocrinology* 35(5)**,** 743-751. doi: 10.1016/j.psyneuen.2009.10.016.

Branchi, I., Santarelli, S., Capoccia, S., Poggini, S., D'Andrea, I., Cirulli, F., et al. (2013). Antidepressant Treatment Outcome Depends on the Quality of the Living Environment: A Pre-Clinical Investigation in Mice. *PLoS ONE* 8(4)**,** e62226. doi: 10.1371/journal.pone.0062226.

Branchi, I., Santarelli, S., D'Andrea, I., and Alleva, E. (2013). Not all stressors are equal: Early social enrichment favors resilience to social but not physical stress in male mice. *Hormones and Behavior* 63(3)**,** 503-509. doi: 10.1016/j.yhbeh.2013.01.003.

Cały, A., Śliwińska, M.A., Ziółkowska, M., Łukasiewicz, K., Pagano, R., Dzik, J.M., et al. (2021). PSD-95 in CA1 Area Regulates Spatial Choice Depending on Age. *The Journal of Neuroscience* 41(11)**,** 2329-2343. doi: 10.1523/JNEUROSCI.1996-20.2020.

Cały, A., Śliwińska, M.A., Ziółkowska, M., Łukasiewicz, K., Pagano, R., Nowacka, A., et al. (2019). Contribution of PSD-95 protein to reward location memory. *bioRxiv*. doi: 10.1101/590109.

Caly, A., Ziolkowska, M., Pagano, R., Salamian, A., Sliwinska, M.A., Sotoudeh, N., et al. (2023). Autophosphorylation of alphaCaMKII regulates alcohol consumption by controlling sedative effects of alcohol and alcohol-induced loss of excitatory synapses. *Addict Biol* 28(5)**,** e13276. doi: 10.1111/adb.13276.

Cao, G., Wei, X., Li, W., Yin, H., Lang, W., Wei, P., et al. (2021). Verification of a multi-function closed maze for the detection of affective disorder and spatial cognitive impairment in post-weaning socially isolated rats. *Neuroscience Letters* 763**,** 136192. doi: 10.1016/j.neulet.2021.136192.

Cathomas, F., Fuertig, R., Sigrist, H., Newman, G.N., Hoop, V., Bizzozzero, M., et al. (2015). CD40-TNF activation in mice induces extended sickness behavior syndrome co-incident with but not dependent on activation of the kynurenine pathway. *Brain, Behavior, and Immunity* 50**,** 125-140. doi: 10.1016/j.bbi.2015.06.184.

Cathomas, F., Sigrist, H., Schmid, L., Seifritz, E., Gassmann, M., Bettler, B., et al. (2017). Behavioural endophenotypes in mice lacking the auxiliary GABA B receptor subunit KCTD16. *Behavioural Brain Research* 317**,** 393-400. doi: 10.1016/j.bbr.2016.10.006.

Cathomas, F., Stegen, M., Sigrist, H., Schmid, L., Seifritz, E., Gassmann, M., et al. (2015). Altered emotionality and neuronal excitability in mice lacking KCTD12, an auxiliary subunit of GABAB receptors associated with mood disorders. *Translational Psychiatry* 5(2)**,** e510-e510. doi: 10.1038/tp.2015.8.

Chen, and de Hoz, L. (2023). The perceptual categorization of multidimensional stimuli is hierarchically organized. *iScience* 26**,** 18. doi: https://doi.org/10.1016/j.isci.2023.106941.

Chen, C., Krueger-Burg, D., and de Hoz, L. (2019). Wide sensory filters underlie performance in memory-based discrimination and generalization. *PLoS ONE* 14(4)**,** e0214817. doi: 10.1371/journal.pone.0214817.

Chotard, É., Mohammadi, F., Julien, P., Berthiaume, L., Rudkowska, I., and Bertrand, N. (2020). Drinkable lecithin nanovesicles to study the biological effects of individual hydrophobic macronutrients and food preferences. *Food Chemistry* 322**,** 126736. doi: 10.1016/j.foodchem.2020.126736.

Chwin, N., Kiryk, A., Bijoch, L., Hamed, A., and Konopka, W. (2019). Up-regulation of PI3K-Akt-mTOR signaling pathway in neurons affects cognitive functions and social interactions in a mouse model. *Acta Neurobiologiae Experimentalis* 79.

Cisbani, G., Poggini, S., Laflamme, N., Pons, V., Tremblay, M.-È., Branchi, I., et al. (2021). The Intellicage system provides a reproducible and standardized method to assess behavioral changes in cuprizone-induced demyelination mouse model. *Behavioural Brain Research* 400**,** 113039. doi: 10.1016/j.bbr.2020.113039.

Codita, A., Gumucio, A., Lannfelt, L., Gellerfors, P., Winblad, B., Mohammed, A.H., et al. (2010). Impaired behavior of female tg-ArcSwe APP mice in the IntelliCage: A longitudinal study. *Behavioural Brain Research* 215(1)**,** 83-94. doi: 10.1016/j.bbr.2010.06.034.

Codita, A., Mohammed, A.H., Willuweit, A., Reichelt, A., Alleva, E., Branchi, I., et al. (2012). Effects of Spatial and Cognitive Enrichment on Activity Pattern and Learning Performance in Three Strains of Mice in the IntelliMaze. *Behavior Genetics* 42(3)**,** 449-460. doi: 10.1007/s10519-011-9512-z.

Coulibaly, A.P. (2022). Neutrophil modulation of behavior and cognition in health and disease: The unexplored role of an innate immune cell. *Immunological Reviews* 311(1)**,** 177-186. doi: 10.1111/imr.13123.

d'Isa, R., Clapcote, S. J., Voikar, V., Wolfer, D. P., Giese, K. P., Brambilla, R., and Fasano, S. (2011). Mice Lacking Ras-GRF1 Show Contextual Fear Conditioning but not Spatial Memory Impairments: Convergent Evidence from Two Independently Generated Mouse Mutant Lines. Front. Behav. Neurosci. 5, 78. doi: 10.3389/fnbeh.2011.00078.

de Hoz, L., Gierej, D., Lioudyno, V., Jaworski, J., Blazejczyk, M., Cruces-Solís, H., et al. (2018). Blocking c-Fos Expression Reveals the Role of Auditory Cortex Plasticity in Sound Frequency Discrimination Learning. *Cerebral Cortex* 28(5)**,** 1645-1655. doi: 10.1093/cercor/bhx060.

de Hoz, L., and Nelken, I. (2014). Frequency Tuning in the Behaving Mouse: Different Bandwidths for Discrimination and Generalization. *PLoS ONE* 9(3)**,** e91676. doi: 10.1371/journal.pone.0091676.

Dere, E., Ronnenberg, A., Tampe, B., Arinrad, S., Schmidt, M., Zeisberg, E., et al. (2018). Cognitive, emotional and social phenotyping of mice in an observer-independent setting. *Neurobiology of Learning and Memory* 150**,** 136-150. doi: 10.1016/j.nlm.2018.02.023.

Dzik, J.M., Puścian, A., Mijakowska, Z., Radwanska, K., and Łęski, S. (2018). PyMICE: A Python library for analysis of IntelliCage data. *Behavior Research Methods* 50(2)**,** 804-815. doi: 10.3758/s13428-017-0907-5.

Dzirkale, Z., Pilipenko, V., Pijet, B., Klimaviciusa, L., Upite, J., Protokowicz, K., Kaczmarek, L., and Jansone, B. (2023). Long-term behavioural alterations in mice following transient cerebral ischemia. Behav. Brain Res. 452, 114589. doi: 10.1016/j.bbr.2023.114589.

Endo, N., Ujita, W., Fujiwara, M., Miyauchi, H., Mishima, H., Makino, Y., et al. (2018). Multiple animal positioning system shows that socially-reared mice influence the social proximity of isolation-reared cagemates. *Communications Biology* 1(1)**,** 225. doi: 10.1038/s42003-018-0213-5.

Endo, T., Kakeyama, M., Uemura, Y., Haijima, A., Okuno, H., Bito, H., et al. (2012). Executive Function Deficits and Social-Behavioral Abnormality in Mice Exposed to a Low Dose of Dioxin In Utero and via Lactation. *PLoS ONE* 7(12)**,** e50741. doi: 10.1371/journal.pone.0050741.

Endo, T., Maekawa, F., Võikar, V., Haijima, A., Uemura, Y., Zhang, Y., et al. (2011). Automated test of behavioral flexibility in mice using a behavioral sequencing task in IntelliCage. *Behavioural Brain Research* 221(1)**,** 172-181. doi: 10.1016/j.bbr.2011.02.037.

Ermakova, O., Piszczek, L., Luciani, L., Cavalli, F.M.G., Ferreira, T., Farley, D., et al. (2011). Sensitized phenotypic screening identifies gene dosage sensitive region on chromosome 11 that

predisposes to disease in mice: Phenotyping of Df11(1) and Dp11(1) mouse lines. *EMBO Molecular Medicine* 3(1)**,** 50-66. doi: 10.1002/emmm.201000112.

Esmaeili, A., Antonova, A., Sitnikova, E., and Smirnov, K. (2022). Whisker trimming during infanthood modifies the development of spike-wave discharges and behavioral sequences in IntelliCage impulsivity paradigm in adult WAG/Rij rats. *Behavioural Brain Research* 418**,** 113627. doi: 10.1016/j.bbr.2021.113627.

Faizi, M., Bader, P.L., Tun, C., Encarnacion, A., Kleschevnikov, A., Belichenko, P., et al. (2011). Comprehensive behavioral phenotyping of Ts65Dn mouse model of Down Syndrome: Activation of β1-adrenergic receptor by xamoterol as a potential cognitive enhancer. *Neurobiology of Disease* 43(2)**,** 397-413. doi: 10.1016/j.nbd.2011.04.011.

Festa, B.P., Berquez, M., Gassama, A., Amrein, I., Ismail, H.M., Samardzija, M., et al. (2019). OCRL deficiency impairs endolysosomal function in a humanized mouse model for Lowe syndrome and Dent disease. *Human Molecular Genetics* 28(12)**,** e1005058. doi: 10.1093/hmg/ddy449.

Fischer, C., Endle, H., Schumann, L., Wilken-Schmitz, A., Kaiser, J., Gerber, S., et al. (2020). Prevention of age-associated neuronal hyperexcitability with improved learning and attention upon knockout or antagonism of LPAR2. *Cellular and Molecular Life Sciences*. doi: 10.1007/s00018-020-03553-4.

Fischer, M., Cabello, V., Popp, S., Krackow, S., Hommers, L., Deckert, J., et al. (2017). Rsk2 Knockout Affects Emotional Behavior in the IntelliCage. *Behavior Genetics* 47(4)**,** 434-448. doi: 10.1007/s10519-017-9853-3.

Fröhlich, H., Kollmeyer, M.L., Linz, V.C., Stuhlinger, M., Groneberg, D., Reigl, A., et al. (2019). Gastrointestinal dysfunction in autism displayed by altered motility and achalasia in *Foxp1*$^{+/-}$ mice. *Proceedings of the National Academy of Sciences* 116(44)**,** 22237-22245. doi: 10.1073/pnas.1911429116.

Frycz, B.A., Nowicka, K., Konopka, A., Hoener, M.C., Bulska, E., Kaczmarek, L., et al. (2023). Activation of trace amine-associated receptor 1 (TAAR1) transiently reduces alcohol drinking in socially housed mice. *Addict Biol* 28(7)**,** e13285. doi: 10.1111/adb.13285.

Fuchs, H., Aguilar-Pimentel, J.A., Amarie, O.V., Becker, L., Calzada-Wack, J., Cho, Y.-L., et al. (2018). Understanding gene functions and disease mechanisms: Phenotyping pipelines in the German Mouse Clinic. *Behavioural Brain Research* 352**,** 187-196. doi: 10.1016/j.bbr.2017.09.048.

Galsworthy, M., Amrein, I., Kuptsov, P., Poletaeva, I., Zinn, P., Rau, A., et al. (2005). A comparison of wild-caught wood mice and bank voles in the Intellicage: assessing exploration, daily activity patterns and place learning paradigms. *Behavioural Brain Research* 157(2)**,** 211-217. doi: 10.1016/j.bbr.2004.06.021.

Gapp, K., Soldado-Magraner, S., Alvarez-Sánchez, M., Bohacek, J., Vernaz, G., Shu, H., et al. (2014). Early life stress in fathers improves behavioural flexibility in their offspring. *Nature Communications* 5(1). doi: 10.1038/ncomms6466.

Garrett, L., Chang, Y.J., Niedermeier, K.M., Heermann, T., Enard, W., Fuchs, H., et al. (2020). A truncating Aspm allele leads to a complex cognitive phenotype and region-specific reductions in parvalbuminergic neurons. *Translational Psychiatry* 10(1)**,** 66. doi: 10.1038/s41398-020-0686-0.

Geraghty, A.C., Gibson, E.M., Ghanem, R.A., Greene, J.J., Ocampo, A., Goldstein, A.K., et al. (2019). Loss of Adaptive Myelination Contributes to Methotrexate Chemotherapy-Related Cognitive Impairment. *Neuron* 103(2)**,** 250-265.e258. doi: 10.1016/j.neuron.2019.04.032.

Goncerzewicz, A., Górkiewicz, T., Dzik, J.M., Jędrzejewska-Szmek, J., Knapska, E., and Konarzewski, M. (2022). Brain size, gut size and cognitive abilities: the energy trade-offs tested in

artificial selection experiment. *Proceedings of the Royal Society B: Biological Sciences* 289(1972), 20212747. doi: 10.1098/rspb.2021.2747.

Grieco, F., Bernstein, B.J., Biemans, B., Bikovski, L., Burnett, C.J., Cushman, J.D., et al. (2021). Measuring Behavior in the Home Cage: Study Design, Applications, Challenges, and Perspectives. *Frontiers in Behavioral Neuroscience* 15, 735387. doi: 10.3389/fnbeh.2021.735387.

Gumucio, A., Lannfelt, L., and Nilsson, L.N.G. (2013). Lack of exon 10 in the murine tau gene results in mild sensorimotor defects with aging. *BMC Neuroscience* 14(1), 148. doi: 10.1186/1471-2202-14-148.

Gundersen, B.B., O'Brien, T.O., Schaffler, M.D., Schultz, M.N., Tsukahara, T., Martin Lorenzo, S., et al. (2023). Towards Preclinical Validation of Arbaclofen (R-baclofen) Treatment for 16p11.2 Deletion Syndrome. *bioRxiv*, 2023.2005.2001.538987. doi: 10.1101/2023.05.01.538987.

Hahnefeld, L., Vogel, A., Gurke, R., Geisslinger, G., Schäfer, M.K.E., and Tegeder, I. (2022). Phosphatidylethanolamine Deficiency and Triglyceride Overload in Perilesional Cortex Contribute to Non-Goal-Directed Hyperactivity after Traumatic Brain Injury in Mice. *Biomedicines* 10(4), 914. doi: 10.3390/biomedicines10040914.

Harda, Z., Dzik, J.M., Nalberczak-Skóra, M., Meyza, K., Łukasiewicz, K., Łęski, S., et al. (2018). Autophosphorylation of αCaMKII affects social interactions in mice. *Genes, Brain and Behavior* 17(5), e12457. doi: 10.1111/gbb.12457.

Harda, Z., Spyrka, J., Jastrzębska, K., Szumiec, Ł., Bryksa, A., Klimczak, M., et al. (2020). Loss of mu and delta opioid receptors on neurons expressing dopamine receptor D1 has no effect on reward sensitivity. *Neuropharmacology* 180, 108307. doi: 10.1016/j.neuropharm.2020.108307.

Hardt, S., Fischer, C., Vogel, A., Wilken-Schmitz, A., and Tegeder, I. (2019). Distal infraorbital nerve injury: a model for persistent facial pain in mice. *PAIN* 160(6), 1431-1447. doi: 10.1097/j.pain.0000000000001518.

Hardt, S., Heidler, J., Albuquerque, B., Valek, L., Altmann, C., Wilken-Schmitz, A., et al. (2017). Loss of synaptic zinc transport in progranulin deficient mice may contribute to progranulin-associated psychopathology and chronic pain. *Biochimica et Biophysica Acta (BBA) - Molecular Basis of Disease* 1863(11), 2727-2745. doi: 10.1016/j.bbadis.2017.07.014.

Heidari, M., Johnstone, D.M., Bassett, B., Graham, R.M., Chua, A.C.G., House, M.J., et al. (2016). Brain iron accumulation affects myelin-related molecular systems implicated in a rare neurogenetic disease family with neuropsychiatric features. *Molecular Psychiatry* 21(11), 1599-1607. doi: 10.1038/mp.2015.192.

Heinla, I., Åhlgren, J., Vasar, E., and Voikar, V. (2018). Behavioural characterization of C57BL/6N and BALB/c female mice in social home cage – Effect of mixed housing in complex environment. *Physiology & Behavior* 188, 32-41. doi: 10.1016/j.physbeh.2018.01.024.

Hikida, T. (2018). Homeostatic regulation of basal ganglia circuit for flexible behavior. *Folia Pharmacologica Japonica*.

Hikida, T., Macpherson, T., and Morita, M. (2017). Basal ganglia circuit mechanisms in cognitive learning. *Japanese Journal of Neuropsychopharmacology* 37(2), 35-38.

Holgate, J.Y., Garcia, H., Chatterjee, S., and Bartlett, S.E. (2017). Social and environmental enrichment has different effects on ethanol and sucrose consumption in mice. *Brain and Behavior* 7(8), e00767. doi: 10.1002/brb3.767.

Hölter, S.M., Garrett, L., Einicke, J., Sperling, B., Dirscherl, P., Zimprich, A., et al. (2015). "Assessing Cognition in Mice: Assessing Cognition in Mice," in *Current Protocols in Mouse Biology,* eds. J. Auwerx, S.L. Ackerman, S.D. Brown, M.J. Justice & J. Nadeau. (Hoboken, NJ, USA: John Wiley & Sons, Inc.), 331-358.

Horigane, S.i., Ozawa, Y., Zhang, J., Todoroki, H., Miao, P., Haijima, A., et al. (2020). A mouse model of Timothy syndrome exhibits altered social competitive dominance and inhibitory neuron development. *FEBS Open Bio* 10(8)**,** 1436-1446. doi: 10.1002/2211-5463.12924.

Hühne, A., Echtler, L., Kling, C., Stephan, M., Schmidt, M.V., Rossner, M.J., et al. (2022). Circadian gene × environment perturbations influence alcohol drinking in *Cryptochrome*-deficient mice. *Addiction Biology* 27(1). doi: 10.1111/adb.13105.

Hühne, A., Volkmann, P., Stephan, M., Rossner, M., and Landgraf, D. (2020). An in-depth neurobehavioral characterization shows anxiety-like traits, impaired habituation behavior, and restlessness in male *Cryptochrome*-deficient mice. *Genes, Brain and Behavior* 19(8). doi: 10.1111/gbb.12661.

Hühne-Landgraf, A., Laurent, K., Frisch, M.K., Wehr, M.C., Rossner, M.J., and Landgraf, D. (2023). Rescue of Comorbid Behavioral and Metabolic Phenotypes of Arrhythmic Mice by Restoring Circadian Cryptochrome1/2 Expression in the Suprachiasmatic Nucleus. *Biological Psychiatry Global Open Science*. doi: https://doi.org/10.1016/j.bpsgos.2023.06.002.

Huo, K., Sun, Y., Li, H., Du, X., Wang, X., Karlsson, N., et al. (2012). Lithium reduced neural progenitor apoptosis in the hippocampus and ameliorated functional deficits after irradiation to the immature mouse brain. *Molecular and Cellular Neuroscience* 51(1-2)**,** 32-42. doi: 10.1016/j.mcn.2012.07.002.

Ikuta, K., Joho, D., Kakeyama, M., and Matsumoto, M. (2023). Bifidobacterium animalis subsp. lactis and arginine mixture intake improves cognitive flexibility in mice. *Frontiers in Nutrition* 10. doi: 10.3389/fnut.2023.1164809.

Iman, I.N., Ahmad, N.A.Z., Mohd Yusof, N.A., Talib, U.N., Norazit, A., Kumar, J., et al. (2021). Mitragynine (Kratom)-Induced Cognitive Impairments in Mice Resemble Delta9-THC and Morphine Effects: Reversal by Cannabinoid CB(1) Receptor Antagonism. *Front Pharmacol* 12**,** 708055. doi: 10.3389/fphar.2021.708055.

Iman, I.N., Yusof, N.A.M., Talib, U.N., Ahmad, N.A.Z., Norazit, A., Kumar, J., et al. (2021). The IntelliCage System: A Review of Its Utility as a Novel Behavioral Platform for a Rodent Model of Substance Use Disorder. *Frontiers in Behavioral Neuroscience* 15**,** 683780. doi: 10.3389/fnbeh.2021.683780.

Ishii, K., Kubo, K.i., Endo, T., Yoshida, K., Benner, S., Ito, Y., et al. (2015). Neuronal Heterotopias Affect the Activities of Distant Brain Areas and Lead to Behavioral Deficits. *Journal of Neuroscience* 35(36)**,** 12432-12445. doi: 10.1523/JNEUROSCI.3648-14.2015.

Ismail, N.I.W., Jayabalan, N., Mansor, S.M., Müller, C.P., and Muzaimi, M. (2017). Chronic mitragynine (kratom) enhances punishment resistance in natural reward seeking and impairs place learning in mice: Mitragynine and cognition. *Addiction Biology* 22(4)**,** 967-976. doi: 10.1111/adb.12385.

Iwata, R., Ohi, K., Kobayashi, Y., Masuda, A., Iwama, M., Yasuda, Y., et al. (2014). RacGAP α2-Chimaerin Function in Development Adjusts Cognitive Ability in Adulthood. *Cell Reports* 8(5)**,** 1257-1264. doi: 10.1016/j.celrep.2014.07.047.

Jablonska, J., Szumiec, L., Zielinski, P., and Parkitna, J.R. (2021). Time elapsed between choices in a probabilistic task correlates with repeating the same decision. *European Journal of Neuroscience* 53(8)**,** 2639-2654. doi: 10.1111/ejn.15144.

Jaholkowski, P., Kiryk, A., Jedynak, P., Ben Abdallah, N.M., Knapska, E., Kowalczyk, A., et al. (2009). New hippocampal neurons are not obligatory for memory formation; cyclin D2 knockout mice with no adult brain neurogenesis show learning. *Learning & Memory* 16(7)**,** 439-451. doi: 10.1101/lm.1459709.

Jastrzębska, K., Walczak, M., Cieślak, P.E., Szumiec, Ł., Turbasa, M., Engblom, D., et al. (2016). Loss of NMDA receptors in dopamine neurons leads to the development of affective disorder-like symptoms in mice. *Scientific Reports* 6(1). doi: 10.1038/srep37171.

Jedynak, P., Jaholkowski, P., Wozniak, G., Sandi, C., Kaczmarek, L., and Filipkowski, R.K. (2012). Lack of cyclin D2 impairing adult brain neurogenesis alters hippocampal-dependent behavioral tasks without reducing learning ability. *Behavioural Brain Research* 227(1)**,** 159-166. doi: 10.1016/j.bbr.2011.11.007.

Jensen, L.R., Garrett, L., Hölter, S.M., Rathkolb, B., Rácz, I., Adler, T., et al. (2019). A mouse model for intellectual disability caused by mutations in the X-linked 2′-O-methyltransferase Ftsj1 gene. *Biochimica et Biophysica Acta (BBA) - Molecular Basis of Disease* 1865(9)**,** 2083-2093. doi: 10.1016/j.bbadis.2018.12.011.

Jensen, M., Ratner, C., Rudenko, O., Christiansen, S.H., Skov, L.J., Hundahl, C., et al. (2016). Anxiolytic-Like Effects of Increased Ghrelin Receptor Signaling in the Amygdala. *International Journal of Neuropsychopharmacology* 19(5)**,** pyv123. doi: 10.1093/ijnp/pyv123.

Jetsonen, E., Didio, G., Winkel, F., Llach Pou, M., Boj, C., Kuczynski-Noyau, L., et al. (2023). Activation of TrkB in Parvalbumin interneurons is required for the promotion of reversal learning in spatial and fear memory by antidepressants. *Neuropsychopharmacology* 48(7)**,** 1021-1030. doi: 10.1038/s41386-023-01562-y.

Jörimann, M., Maliković, J., Wolfer, D.P., Pryce, C.R., Endo, T., Benner, S., et al. (2023). Bank Voles Show More Impulsivity in IntelliCage Learning Tasks than Wood Mice. *Neuroscience* 510**,** 157-170. doi: 10.1016/j.neuroscience.2022.11.011.

Juskeviciene, R., Fritz, A.-K., Brilkova, M., Akbergenov, R., Schmitt, K., Rehrauer, H., et al. (2022). Phenotype of Mrps5-Associated Phylogenetic Polymorphisms Is Intimately Linked to Mitoribosomal Misreading. *International Journal of Molecular Sciences* 23(8)**,** 4384. doi: 10.3390/ijms23084384.

Kahnau, P., Guenther, A., Boon, M.N., Terzenbach, J.D., Hanitzsch, E., Lewejohann, L., et al. (2021). Lifetime Observation of Cognition and Physiological Parameters in Male Mice. *Frontiers in Behavioral Neuroscience* 15**,** 709775. doi: 10.3389/fnbeh.2021.709775.

Kahnau, P., Jaap, A., Diederich, K., Gygax, L., Rudeck, J., and Lewejohann, L. (2022). Determining the value of preferred goods based on consumer demand in a home-cage based test for mice. *Behavior Research Methods*. doi: 10.3758/s13428-022-01813-8.

Kahnau, P., Jaap, A., Urmersbach, B., Diederich, K., and Lewejohann, L. (2023). Development of an IntelliCage based Cognitive Bias Test for Mice. *Open Research Europe 2022* 2:128. doi: https://doi.org/10.12688/openreseurope.15294.1.

Kahnau, P., Mieske, P., Wilzopolski, J., Kalliokoski, O., Mandillo, S., Hölter, S.M., et al. (2023). Development and Application of Home Cage Monitoring in Laboratory Mice and Rats: a Systematic Review. bioRxiv).

Kalm, M., Andreasson, U., Björk-Eriksson, T., Zetterberg, H., Pekny, M., Blennow, K., et al. (2016). C3 deficiency ameliorates the negative effects of irradiation of the young brain on hippocampal development and learning. *Oncotarget* 7(15). doi: 10.18632/oncotarget.8400.

Kalm, M., Karlsson, N., Nilsson, M.K.L., and Blomgren, K. (2013). Loss of hippocampal neurogenesis, increased novelty-induced activity, decreased home cage activity, and impaired reversal learning one year after irradiation of the young mouse brain. *Experimental Neurology* 247**,** 402-409. doi: 10.1016/j.expneurol.2013.01.006.

Kandadai, K.S., Kotur, M.B., Dokalis, N., Amrein, I., Keller, C.W., Munz, C., et al. (2021). ATG5 in microglia does not contribute vitally to autoimmune neuroinflammation in mice. *Autophagy* 17(11)**,** 3566-3576. doi: 10.1080/15548627.2021.1883880.

Karlsson, N., Kalm, M., Nilsson, M.K.L., Mallard, C., Björk-Eriksson, T., and Blomgren, K. (2011). Learning and Activity after Irradiation of the Young Mouse Brain Analyzed in Adulthood Using Unbiased Monitoring in a Home Cage Environment. *Radiation Research* 175(3)**,** 336-346. doi: 10.1667/RR2231.1.

Kato, T.M., Kubota-Sakashita, M., Fujimori-Tonou, N., Saitow, F., Fuke, S., Masuda, A., et al. (2018). Ant1 mutant mice bridge the mitochondrial and serotonergic dysfunctions in bipolar disorder. *Molecular Psychiatry* 23(10)**,** 2039-2049. doi: 10.1038/s41380-018-0074-9.

Kempermann, G., Lopes, J.B., Zocher, S., Schilling, S., Ehret, F., Garthe, A., et al. (2022). The individuality paradigm: Automated longitudinal activity tracking of large cohorts of genetically identical mice in an enriched environment. *Neurobiology of Disease* 175**,** 105916. doi: 10.1016/j.nbd.2022.105916.

Kimura, E., Suzuki, G., Uramaru, N., Endo, T., and Maekawa, F. (2020). Behavioral impairments in infant and adult mouse offspring exposed to 2,3,7,8-tetrabromodibenzofuran in utero and via lactation. *Environment International* 142**,** 105833. doi: 10.1016/j.envint.2020.105833.

Kiryk, A., Aida, T., Tanaka, K., Banerjee, P., Wilczynski, G.M., Meyza, K., et al. (2008). Behavioral characterization of GLT1 (+/-) mice as a model of mild glutamatergic hyperfunction. *Neurotoxicity Research* 13(1)**,** 19-30. doi: 10.1007/BF03033364.

Kiryk, A., Janusz, A., Zglinicki, B., Turkes, E., Knapska, E., Konopka, W., Lipp, H.-P., and Kaczmarek, L. (2020). IntelliCage as a tool for measuring mouse behavior – 20 years perspective. *Behavioural Brain Research* 388**,** 112620. doi: 10.1016/j.bbr.2020.112620.

Kiryk, A., Mochol, G., K. Filipkowski, R., Wawrzyniak, M., Lioudyno, V., Knapska, E., et al. (2011). Cognitive Abilities of Alzheimers Disease Transgenic Mice are Modulated by Social Context and Circadian Rhythm. *Current Alzheimer Research* 8(8)**,** 883-892. doi: 10.2174/156720511798192745.

Klein, C.J.M.I., Budiman, T., Homberg, J.R., Verma, D., Keijer, J., and van Schothorst, E.M. (2022). Measuring Locomotor Activity and Behavioral Aspects of Rodents Living in the Home-Cage. *Frontiers in Behavioral Neuroscience* 16. doi: 10.3389/fnbeh.2022.877323.

Knapska, E., Walasek, G., Nikolaev, E., Neuhäusser-Wespy, F., Lipp, H.-P., and Kaczmarek, L. (2006). Differential involvement of the central amygdala in appetitive versus aversive learning. *Learn Mem* 13(2)**,** 192-200. doi: 10.1101/lm.54706.

Knapska, E., Lioudyno, V., Kiryk, A., Mikosz, M., Gorkiewicz, T., Michaluk, P., et al. (2013). Reward Learning Requires Activity of Matrix Metalloproteinase-9 in the Central Amygdala. *Journal of Neuroscience* 33(36)**,** 14591-14600. doi: 10.1523/JNEUROSCI.5239-12.2013.

Kobayashi, Y., Sano, Y., Vannoni, E., Goto, H., Suzuki, H., Oba, A., et al. (2013). Genetic dissection of medial habenula–interpeduncular nucleus pathway function in mice. *Frontiers in Behavioral Neuroscience* 7. doi: 10.3389/fnbeh.2013.00017.

Konarzewski, M., Goncerzewicz, A., Knapska, E., Dzik, J., and rkiewicz, T.G. (2020). Energetic Costs of Cognitive Abilities: Testing the Expensive Tissue Hypothesis. *Authorea*. doi: 10.22541/au.159069206.66900218.

Konopka, W., Kiryk, A., Novak, M., Herwerth, M., Parkitna, J.R., Wawrzyniak, M., et al. (2010). MicroRNA Loss Enhances Learning and Memory in Mice. *Journal of Neuroscience* 30(44)**,** 14835-14842. doi: 10.1523/JNEUROSCI.3030-10.2010.

Koskela, M., Piepponen, T.P., Andressoo, J.-O., Võikar, V., and Airavaara, M. (2018). Towards developing a model to study alcohol drinking and craving in female mice housed in automated cages. *Behavioural Brain Research* 352**,** 116-124. doi: 10.1016/j.bbr.2018.03.027.

Koskela, M., Piepponen, T.P., Andressoo, J.-O., Võikar, V., and Airavaara, M. (2021). Female C57BL/6J Mice Show Alcohol-Seeking Behaviour after Withdrawal from Prolonged Alcohol Consumption in the Social Environment. *Alcohol and Alcoholism***,** agab032. doi: 10.1093/alcalc/agab032.

Koskela, M., Piepponen, T.P., Lindahl, M., Harvey, B.K., Andressoo, J.-O., Võikar, V., et al. (2021). The overexpression of GDNF in nucleus accumbens suppresses alcohol-seeking behavior in group-housed C57Bl/6J female mice. *Journal of Biomedical Science* 28(1)**,** 87. doi: 10.1186/s12929-021-00782-y.

Koss, D.J., Robinson, L., Drever, B.D., Plucińska, K., Stoppelkamp, S., Veselcic, P., et al. (2016). Mutant Tau knock-in mice display frontotemporal dementia relevant behaviour and histopathology. *Neurobiology of Disease* 91**,** 105-123. doi: 10.1016/j.nbd.2016.03.002.

Krackow, S., Vannoni, E., Codita, A., Mohammed, A.H., Cirulli, F., Branchi, I., et al. (2010). Consistent behavioral phenotype differences between inbred mouse strains in the IntelliCage. *Genes, Brain and Behavior* 9(7)**,** 722-731. doi: 10.1111/j.1601-183X.2010.00606.x.

Kraft, V., Schmitz, K., Wilken-Schmitz, A., Geisslinger, G., Sisignano, M., and Tegeder, I. (2021). Trehalose Reduces Nerve Injury Induced Nociception in Mice but Negatively Affects Alertness. *Nutrients* 13(9)**,** 2953. doi: 10.3390/nu13092953.

Kulesskaya, N., Karpova, N.N., Ma, L., Tian, L., and Voikar, V. (2014). Mixed housing with DBA/2 mice induces stress in C57BL/6 mice: implications for interventions based on social enrichment. *Frontiers in Behavioral Neuroscience* 8. doi: 10.3389/fnbeh.2014.00257.

Kulesskaya, N., Võikar, V., Peltola, M., Yegutkin, G.G., Salmi, M., Jalkanen, S., et al. (2013). CD73 Is a Major Regulator of Adenosinergic Signalling in Mouse Brain. *PLoS ONE* 8(6)**,** e66896. doi: 10.1371/journal.pone.0066896.

Lan, W.-C.J., Priestley, M., Mayoral, S.R., Tian, L., Shamloo, M., and Penn, A.A. (2011). Sex-Specific Cognitive Deficits and Regional Brain Volume Loss in Mice Exposed to Chronic, Sublethal Hypoxia. *Pediatric Research* 70(1)**,** 15-20. doi: 10.1203/PDR.0b013e31821b98a3.

Langfelder, P., Gao, F., Wang, N., Howland, D., Kwak, S., Vogt, T.F., et al. (2018). MicroRNA signatures of endogenous Huntingtin CAG repeat expansion in mice. *PLoS ONE* 13(1)**,** e0190550. doi: 10.1371/journal.pone.0190550.

Lebedeva-Georgievskaya, K.B., Matveeva, M.I., Bazian, A.S., Kudrin, V.S., Narkevich, V.B., Perevezentsev, A.A., et al. (2017). Effect of tail-suspension on the activity, environmental adaptation, exploratory behavior and monoamine turnover in the brain of mice. *Aerospace and Environmental Medicine* 51(1)**,** 39-45. doi: 10.21687/0233-528X-2017-51-1-39-45.

Lebitko, T., Dzik, J., Jędrzejewska-Szmek, J., Chaturvedi, M., Jaworski, T., Nikolaev, T., et al. (2020). c-Fos-MMP-9 pathway in central amygdala mediates approach motivation but not reward consumption. *bioRxiv*. doi: 10.1101/2020.04.17.044792.

Lee, K., Kobayashi, Y., Seo, H., Kwak, J.-H., Masuda, A., Lim, C.-S., et al. (2015). Involvement of cAMP-guanine nucleotide exchange factor II in hippocampal long-term depression and behavioral flexibility. *Molecular Brain* 8(1). doi: 10.1186/s13041-015-0130-1.

Leite-Almeida, H., Castelhano-Carlos, M.J., and Sousa, N. (2022). New Horizons for Phenotyping Behavior in Rodents: The Example of Depressive-Like Behavior. *Frontiers in Behavioral Neuroscience* 15**,** 811987. doi: 10.3389/fnbeh.2021.811987.

Li, L., Wang, Q., Sun, X., Li, Z., Liu, S., Zhang, X., et al. (2023). Activation of RhoA pathway participated in the changes of emotion, cognitive function and hippocampal synaptic plasticity in juvenile chronic stress rats. *International Journal of Biological Macromolecules* 233**,** 123652. doi: https://doi.org/10.1016/j.ijbiomac.2023.123652.

Li, X., Gao, Y., Han, X., Tang, S., Li, N., Liu, X., et al. (2023). Maresin1 ameliorates postoperative cognitive dysfunction in aged rats by potentially regulating the NF-kappaB pathway to inhibit astrocyte activation. *Exp Gerontol* 176**,** 112168. doi: 10.1016/j.exger.2023.112168.

Lipp, H.-P. (2005). High-throughput and Automated Behavioural Screening of Normal and Genetically Modified Mice. *Business Briefing: Future Drug Discovery***,** 5.

Lipp, H.-P., and Wolfer, D.P. (2022). Behavior is movement only but how to interpret it? Problems and pitfalls in translational neuroscience—a 40-year experience. *Frontiers in Behavioral Neuroscience* 16**,** 958067. doi: 10.3389/fnbeh.2022.958067.

Lipp, H.P., Galsworthy, M., Vyssotski, D.L., Zinn, P., Rau, A.E., Neuhäusser-Wespy, F., et al. (2005). Automated behavioral analysis of mice using INTELLICAGE: inter-laboratory comparisons and validation with exploratory behavior and spatial learning. *Proceedings of Measuring Behaviour 2005***,** 66-69.

Liu, S., Jin, Z., Zhang, Y., Rong, S., He, W., Sun, K., et al. (2020). The Glucagon-Like Peptide-1 Analogue Liraglutide Reduces Seizures Susceptibility, Cognition Dysfunction and Neuronal Apoptosis in a Mouse Model of Dravet Syndrome. *Front Pharmacol* 11**,** 136. doi: 10.3389/fphar.2020.00136.

Liu, Y., Burton, T., Rayner, B.S., San Gabriel, P.T., Shi, H., El Kazzi, M., et al. (2020). The role of sodium thiocyanate supplementation during dextran sodium sulphate-stimulated experimental colitis. *Archives of Biochemistry and Biophysics* 692**,** 108490. doi: 10.1016/j.abb.2020.108490.

Lopez-Caperuchipi, S. (2022). *Charakterisierung zellulärer Veränderungen und kognitiver Verhaltensweisen in einem Model vom Schädel-Hirn Trauma in männlichen Mäusen.* MD, Julius-Maximilians-Universität Würzburg,.

Lopez-Caperuchipi, S., Kürzinger, L., Hopp-Krämer, S., Albert-Weißenberger, C., Paul, M.M., Sirén, A.-L., et al. (2021). Posttraumatic learning deficits correlate with initial trauma severity and chronic cellular reactions after closed head injury in male mice. *Experimental Neurology* 341**,** 113721. doi: 10.1016/j.expneurol.2021.113721.

Macpherson, T., Morita, M., Wang, Y., Sasaoka, T., Sawa, A., and Hikida, T. (2016). Nucleus accumbens dopamine D2-receptor expressing neurons control behavioral flexibility in a place discrimination task in the IntelliCage. *Learning & Memory* 23(7)**,** 359-364. doi: 10.1101/lm.042507.116.

Markova, E.V., and Knyazheva, M.A. (2021). IMMUNE CELLS AS A POTENTIAL THERAPEUTIC AGENT IN THE TREATMENT OF DEPRESSION. *Medical Immunology (Russia)* 23(4)**,** 699-704. doi: 10.15789/1563-0625-ICA-2277.

Markova, E.V., Knyazheva, M.A., Tikhonova, M.A., and Amstislavskaya, T.G. (2022). Structural and functional characteristics of the hippocampus in depressive-like recipients after transplantation of in vitro caffeine-modulated immune cells. *Neuroscience Letters* 786**,** 136790. doi: 10.1016/j.neulet.2022.136790.

Maroteaux, G., Arefin, T.M., Harsan, L.A., Darcq, E., Ben Hamida, S., and Kieffer, B.L. (2018). Lack of anticipatory behavior in Gpr88 knockout mice showed by automatized home cage phenotyping. *Genes, Brain and Behavior* 17(8)**,** e12473. doi: 10.1111/gbb.12473.

Marwari, S., and Dawe, G.S. (2018). (R)-fluoxetine enhances cognitive flexibility and hippocampal cell proliferation in mice. *Journal of Psychopharmacology* 32(4)**,** 441-457. doi: 10.1177/0269881118754733.

Marwari, S., and Dawe, G.S. (2019). Effects of haloperidol on cognitive function and behavioural flexibility in the IntelliCage social home cage environment. *Behavioural Brain Research* 371**,** 111976. doi: 10.1016/j.bbr.2019.111976.

Masuda, A., Kobayashi, Y., and Itohara, S. (2018). Automated, Long-term Behavioral Assay for Cognitive Functions in Multiple Genetic Models of Alzheimer's Disease, Using IntelliCage. *Journal of Visualized Experiments* (138). doi: 10.3791/58009.

Masuda, A., Kobayashi, Y., Kogo, N., Saito, T., Saido, T.C., and Itohara, S. (2016). Cognitive deficits in single App knock-in mouse models. *Neurobiology of Learning and Memory* 135**,** 73-82. doi: 10.1016/j.nlm.2016.07.001.

Mateusz, S.L., Andrzej, L.M., Ewa, L., Joanna, U.-C., Ludwika, K., Puscian, A., et al. (2020). β-catenin signaling via astrocyte-encoded TCF7L2 regulates neuronal excitability and social behavior. *bioRxiv*. doi: 10.1101/2020.11.28.402099.

Mätlik, K., Garton, D.R., Montaño-Rodríguez, A.R., Olfat, S., Eren, F., Casserly, L., et al. (2022). Elevated endogenous GDNF induces altered dopamine signalling in mice and correlates with clinical severity in schizophrenia. *Molecular Psychiatry* 27(8)**,** 3247-3261. doi: 10.1038/s41380-022-01554-2.

Mätlik, K., Võikar, V., Vilenius, C., Kulesskaya, N., and Andressoo, J.-O. (2018). Two-fold elevation of endogenous GDNF levels in mice improves motor coordination without causing side-effects. *Scientific Reports* 8(1). doi: 10.1038/s41598-018-29988-1.

Mechan, A.O., Wyss, A., Rieger, H., and Mohajeri, M.H. (2009). A comparison of learning and memory characteristics of young and middle-aged wild-type mice in the IntelliCage. *Journal of Neuroscience Methods* 180(1)**,** 43-51. doi: 10.1016/j.jneumeth.2009.02.018.

Mehr, A., Hick, M., Ludewig, S., Müller, M., Herrmann, U., von Engelhardt, J., et al. (2020). Lack of APP and APLP2 in GABAergic Forebrain Neurons Impairs Synaptic Plasticity and Cognition. *Cerebral Cortex* 30(7)**,** 4044-4063. doi: 10.1093/cercor/bhaa025.

Menalled, L.B., Kudwa, A.E., Miller, S., Fitzpatrick, J., Watson-Johnson, J., Keating, N., et al. (2012). Comprehensive Behavioral and Molecular Characterization of a New Knock-In Mouse Model of Huntington's Disease: zQ175. *PLoS ONE* 7(12)**,** e49838. doi: 10.1371/journal.pone.0049838.

Menalled, L.B., Kudwa, A.E., Oakeshott, S., Farrar, A., Paterson, N., Filippov, I., et al. (2014). Genetic Deletion of Transglutaminase 2 Does Not Rescue the Phenotypic Deficits Observed in R6/2 and zQ175 Mouse Models of Huntington's Disease. *PLoS ONE* 9(6)**,** e99520. doi: 10.1371/journal.pone.0099520.

Meng, H.-R., Suenaga, T., Edamura, M., Fukuda, A., Ishida, Y., Nakahara, D., et al. (2021). Functional MHCI deficiency induces ADHD-like symptoms with increased dopamine D1 receptor expression. *Brain, Behavior, and Immunity* 97**,** 22-31. doi: 10.1016/j.bbi.2021.05.015.

Mifflin, M.A., Winslow, W., Surendra, L., Tallino, S., Vural, A., and Velazquez, R. (2021). Sex differences in the IntelliCage and the Morris water maze in the APP/PS1 mouse model of amyloidosis. *Neurobiology of Aging* 101**,** 130-140. doi: 10.1016/j.neurobiolaging.2021.01.018.

Mijakowska, Z., Łukasiewicz, K., Ziółkowska, M., Lipiński, M., Trąbczyńska, A., Matuszek, Ż., et al. (2017). Autophosphorylation of alpha isoform of calcium/calmodulin-dependent kinase II regulates alcohol addiction-related behaviors: αCaMKII and alcohol addiction. *Addiction Biology* 22(2)**,** 331-341. doi: 10.1111/adb.12327.

Milior, G., Lecours, C., Samson, L., Bisht, K., Poggini, S., Pagani, F., et al. (2016). Fractalkine receptor deficiency impairs microglial and neuronal responsiveness to chronic stress. *Brain, Behavior, and Immunity* 55**,** 114-125. doi: 10.1016/j.bbi.2015.07.024.

Mingrone, A., Kaffman, A., and Kaffman, A. (2020). The Promise of Automated Home-Cage Monitoring in Improving Translational Utility of Psychiatric Research in Rodents. *Frontiers in Neuroscience* 14**,** 618593. doi: 10.3389/fnins.2020.618593.

Mitjans, M., Begemann, M., Ju, A., Dere, E., Wüstefeld, L., Hofer, S., et al. (2017). Sexual dimorphism of AMBRA1-related autistic features in human and mouse. *Translational Psychiatry* 7(10), e1247. doi: 10.1038/tp.2017.213.

Mohammadi, F., Bertrand, N., and Rudkowska, I. (2023). C57bl/6 Mice Show Equivalent Taste Preferences toward Ruminant and Industrial Trans Fatty Acids. *Nutrients* 15(3). doi: 10.3390/nu15030610.

Mohammadi, S.A., Burton, T.J., and Christie, M.J. (2017). α9-nAChR knockout mice exhibit dysregulation of stress responses, affect and reward-related behaviour. *Behavioural Brain Research* 328, 105-114. doi: 10.1016/j.bbr.2017.04.005.

Morello, F., Voikar, V., Parkkinen, P., Panhelainen, A., Rosenholm, M., Makkonen, A., et al. (2020). ADHD-like behaviors caused by inactivation of a transcription factor controlling the balance of inhibitory and excitatory neuron development in the mouse anterior brainstem. *Translational Psychiatry* 10(1), 357. doi: 10.1038/s41398-020-01033-8.

Morozova, M.V., Borisova, M.A., Snytnikova, O.A., Achasova, K.M., Litvinova, E.A., Tsentalovich, Y.P., et al. (2022). Colitis-associated intestinal microbiota regulates brain glycine and host behavior in mice. *bioRxiv*. doi: 10.1101/2022.03.07.483210.

Muthuraju, S., Pati, S., Rafiqul, M., Abdullah, J.M., and Jaafar, H. (2012). IntelliCage provides voluntary exercise and an enriched environment, improving locomotive activity in mice following fluid percussion injury. *Basal Ganglia* 2(3), 143-151. doi: 10.1016/j.baga.2012.06.004.

Muthuraju, S., Taha, S., Pati, S., Rafique, M., Jaafar, H., and Abdullah, J.M. (2013). Normabaric Hyperoxia Treatment Improved Locomotor Activity of C57BL/6J Mice through Enhancing Dopamine Genes Following Fluid-Percussion Injury in Striatum. *International Journal of Biomedical Science* 9(4), 11.

Nagaeva, E., Schäfer, A., Linden, A.-M., Elsilä, L.V., Ryazantseva, M., Umemori, J., et al. (2023). Somatostatin-expressing neurons in the ventral tegmental area innervate specific forebrain regions and are involved in the stress response. *bioRxiv*.

Nakamura, T., Nakajima, K., Kobayashi, Y., Itohara, S., Kasahara, T., Tsuboi, T., et al. (2021). Functional and behavioral effects of <i>de novo</i> mutations in calcium-related genes in patients with bipolar disorder. *Human Molecular Genetics* 30(19), 1851-1862. doi: 10.1093/hmg/ddab152.

Nalberczak-Skóra, M., Beroun, A., Skonieczna, E., Cały, A., Ziółkowska, M., Pagano, R., et al. (2023). Impaired synaptic transmission in dorsal dentate gyrus increases impulsive alcohol seeking. *Neuropsychopharmacology* 48(3), 436-447. doi: 10.1038/s41386-022-01464-5.

Netrakanti, P.R., Cooper, B.H., Dere, E., Poggi, G., Winkler, D., Brose, N., et al. (2015). Fast Cerebellar Reflex Circuitry Requires Synaptic Vesicle Priming by Munc13-3. *The Cerebellum* 14(3), 264-283. doi: 10.1007/s12311-015-0645-0.

Nieraad, H., de Bruin, N., Arne, O., Hofmann, M.C.J., Schmidt, M., Saito, T., et al. (2020). Impact of Hyperhomocysteinemia and Different Dietary Interventions on Cognitive Performance in a Knock-in Mouse Model for Alzheimer's Disease. *Nutrients* 12(11), 3248. doi: 10.3390/nu12113248.

Niiranen, L., Stenback, V., Tulppo, M., Herzig, K.H., and Makela, K. (2023). Interplay between Learning and Voluntary Wheel Running in Male C57BL/6NCrl Mice. *International Journal of Molecular Sciences* 24(5). doi: ARTN 4259

10.3390/ijms24054259.

Nowak, A., Werka, T., and Knapska, E. (2013). Social modulation in extinction of aversive memories. *Behavioural Brain Research* 238**,** 200-205. doi: 10.1016/j.bbr.2012.10.031.

Oakeshott, S., Balci, F., Filippov, I., Murphy, C., Port, R., Connor, D., et al. (2011). Circadian Abnormalities in Motor Activity in a BAC Transgenic Mouse Model of Huntington's Disease. *PLoS Currents* 3**,** RRN1225. doi: 10.1371/currents.RRN1225.

Oakeshott, S., Port, R., Cummins-Sutphen, J., Berger, J., Watson-Johnson, J., Ramboz, S., et al. (2012). A mixed fixed ratio/progressive ratio procedure reveals an apathy phenotype in the BAC HD and the z_Q175 KI mouse models of Huntington's disease. *PLoS Currents*. doi: 10.1371/4f972cffe82c0.

Ogi, H., Itoh, K., and Fushiki, S. (2013). Social behavior is perturbed in mice after exposure to bisphenol A: a novel assessment employing an IntelliCage. *Brain and Behavior* 3(3)**,** 223-228. doi: 10.1002/brb3.130.

Ogi, H., Itoh, K., Ikegaya, H., and Fushiki, S. (2015). Alterations of neurotransmitter norepinephrine and gamma-aminobutyric acid correlate with murine behavioral perturbations related to bisphenol A exposure. *Brain and Development* 37(8)**,** 739-746. doi: 10.1016/j.braindev.2014.12.008.

Oizumi, H., Miyazaki, S., Tabuchi, M., Endo, T., Omiya, Y., and Mizoguchi, K. (2020). Kamikihito Enhances Cognitive Functions and Reward-Related Behaviors of Aged C57BL/6J Mice in an Automated Behavioral Assay System. *Frontiers in Pharmacology* 11**,** 1037. doi: 10.3389/fphar.2020.01037.

Ojanen, S., Kuznetsova, T., Kharybina, Z., Voikar, V., Lauri, S.E., and Taira, T. (2023). Interneuronal GluK1 kainate receptors control maturation of GABAergic transmission and network synchrony in the hippocampus. *Mol Brain* 16(1)**,** 43. doi: 10.1186/s13041-023-01035-9.

Oliveros, E., Vázquez, E., Barranco, A., Ramírez, M., Gruart, A., Delgado-García, J., et al. (2018). Sialic Acid and Sialylated Oligosaccharide Supplementation during Lactation Improves Learning and Memory in Rats. *Nutrients* 10(10)**,** 1519. doi: 10.3390/nu10101519.

Onishchenko, N., Tamm, C., Vahter, M., Hökfelt, T., Johnson, J.A., Johnson, D.A., et al. (2007). Developmental Exposure to Methylmercury Alters Learning and Induces Depression-like Behavior in Male Mice. *Toxicological Sciences* 97(2)**,** 428-437. doi: 10.1093/toxsci/kfl199.

Orock, A., Logan, S., and Deak, F. (2018). Munc18-1 haploinsufficiency impairs learning and memory by reduced synaptic vesicular release in a model of Ohtahara syndrome. *Molecular and Cellular Neuroscience* 88**,** 33-42. doi: 10.1016/j.mcn.2017.12.002.

Osman, A.M., Zhou, K., Zhu, C., and Blomgren, K. (2014). Transplantation of Enteric Neural Stem/Progenitor Cells into the Irradiated Young Mouse Hippocampus. *Cell Transplantation* 23(12)**,** 1657-1671. doi: 10.3727/096368913X674648.

Pagano, R., Salamian, A., Zielinski, J., Beroun, A., Nalberczak-Skóra, M., Skonieczna, E., et al. (2022). Arc controls alcohol cue relapse by a central amygdala mechanism. *Molecular Psychiatry*. doi: 10.1038/s41380-022-01849-4.

Pan, H., Oliveira, B., Saher, G., Dere, E., Tapken, D., Mitjans, M., et al. (2018). Uncoupling the widespread occurrence of anti-NMDAR1 autoantibodies from neuropsychiatric disease in a novel autoimmune model. *Molecular Psychiatry*. doi: 10.1038/s41380-017-0011-3.

Parkitna, J.R., Sikora, M., Gołda, S., Gołembiowska, K., Bystrowska, B., Engblom, D., et al. (2013). Novelty-Seeking Behaviors and the Escalation of Alcohol Drinking After Abstinence in Mice Are Controlled by Metabotropic Glutamate Receptor 5 on Neurons Expressing Dopamine D1 Receptors. *Biological Psychiatry* 73(3)**,** 263-270. doi: 10.1016/j.biopsych.2012.07.019.

Patrikainen, M., Pan, P., Kulesskaya, N., Voikar, V., and Parkkila, S. (2014). The role of carbonic anhydrase VI in bitter taste perception: evidence from the Car6 −/− mouse model. *Journal of Biomedical Science* 21(1). doi: 10.1186/s12929-014-0082-2.

Pelsőczi, P., Kelemen, K., Csölle, C., Nagy, G., Lendvai, B., Román, V., et al. (2020). Disrupted Social Hierarchy in Prenatally Valproate-Exposed Autistic-Like Rats. *Frontiers in Behavioral Neuroscience* 13**,** 295. doi: 10.3389/fnbeh.2019.00295.

Pelsőczi, P., and Lévay, G. (2017). Effect of Scopolamine on Mice Motor Activity, Lick Behavior and Reversal Learning in the IntelliCage. *Neurochemical Research* 42(12)**,** 3597-3602. doi: 10.1007/s11064-017-2408-4.

Peltola, M.A., Kuja-Panula, J., Liuhanen, J., Võikar, V., Piepponen, P., Hiekkalinna, T., et al. (2015). AMIGO-Kv2.1 Potassium Channel Complex is Associated With Schizophrenia-Related Phenotypes. *Schizophrenia Bulletin***,** sbv105. doi: 10.1093/schbul/sbv105.

Perez-Alcazar, M., Daborg, J., Stokowska, A., Wasling, P., Björefeldt, A., Kalm, M., et al. (2014). Altered cognitive performance and synaptic function in the hippocampus of mice lacking C3. *Experimental Neurology* 253**,** 154-164. doi: 10.1016/j.expneurol.2013.12.013.

Perschler, L. (2023). *Die Effekte von Mitragynin auf den Alkoholkonsum beim sozialen Trinken.* MD, Friedrich-Alexander-Universität Erlangen-Nürnberg.

Perschler, L. (2023). *Die Effekte von Mitragynin auf den Alkoholkonsum beim sozialen Trinken : Eine präklinische Studie im Mausmodell.*

Pham, H., Yin, T., and D'Adamio, L. (2022). Initial assessment of the spatial learning, reversal, and sequencing task capabilities of knock- in rats with humanizing mutations in the Aβ-coding region of *App*. *bioRxiv*. doi: 10.1101/2022.01.24.477482.

Picard, K., Bisht, K., Poggini, S., Garofalo, S., Golia, M.T., Basilico, B., et al. (2021). Microglial-glucocorticoid receptor depletion alters the response of hippocampal microglia and neurons in a chronic unpredictable mild stress paradigm in female mice. *Brain, Behavior, and Immunity* 97**,** 423-439. doi: 10.1016/j.bbi.2021.07.022.

Piechota, M., Korostynski, M., Sikora, M., Golda, S., Dzbek, J., and Przewlocki, R. (2012). Common transcriptional effects in the mouse striatum following chronic treatment with heroin and methamphetamine. *Genes, Brain and Behavior* 11(4)**,** 404-414. doi: 10.1111/j.1601-183X.2012.00777.x.

Plum, T., Binzberger, R., Thiele, R., Shang, F., Postrach, D., Fung, C., et al. (2023). Mast cells link immune sensing to antigen-avoidance behaviour. *Nature*. doi: 10.1038/s41586-023-06188-0.

Poggini, S., Golia, M.T., Alboni, S., Milior, G., Sciarria, L.P., Viglione, A., et al. (2019). Combined Fluoxetine and Metformin Treatment Potentiates Antidepressant Efficacy Increasing IGF2 Expression in the Dorsal Hippocampus. *Neural Plasticity* 2019**,** 1-12. doi: 10.1155/2019/4651031.

Poggini, S., Lopez, M.B., Albanese, N.C., Golia, M.T., Ibáñez, F.G., Limatola, C., et al. (2023). Minocycline treatment improves cognitive and functional plasticity in a preclinical mouse model of major depressive disorder. *Behavioural Brain Research***,** 114295. doi: 10.1016/j.bbr.2023.114295.

Poggini, S., Matte Bon, G., Golia, M.T., Ciano Albanese, N., Viglione, A., Poleggi, A., et al. (2021). Selecting antidepressants according to a drug-by-environment interaction: A comparison of fluoxetine and minocycline effects in mice living either in enriched or stressful conditions. *Behavioural Brain Research* 408**,** 113256. doi: 10.1016/j.bbr.2021.113256.

Pupikina, M., and Sitnikova, E. (2023). Sex Differences in Behavior and Learning Abilities in Adult Rats. *Life (Basel)* 13(2). doi: 10.3390/life13020547.

Puścian, A., and Knapska, E. (2022). Blueprints for measuring natural behavior. *iScience* 25(7), 104635. doi: 10.1016/j.isci.2022.104635.

Puścian, A., Łęski, S., Gorkiewicz, T., Meyza, K., Lipp, H.-P., and Knapska, E. (2014). A novel automated behavioral test battery assessing cognitive rigidity in two genetic mouse models of autism. *Frontiers in Behavioral Neuroscience* 8. doi: 10.3389/fnbeh.2014.00140.

Puścian, A., Winiarski, M., Borowska, J., Łęski, S., Górkiewicz, T., Chaturvedi, M., et al. (2022). Targeted therapy of cognitive deficits in fragile X syndrome. *Molecular Psychiatry*. doi: 10.1038/s41380-022-01527-5.

Puścian, A., Winiarski, M., Łęski, S., Charzewski, Ł., Nikolaev, T., Borowska, J., et al. (2020). Chronic fluoxetine treatment impairs motivation and reward learning by affecting neuronal plasticity in the central amygdala. *British Journal of Pharmacology*, bph.15319. doi: 10.1111/bph.15319.

Qiu, H., Li, S., and Le, W. (2015). Impacts of chronic sleep deprivation on learning and memory, autophagy and neuronal apoptosis in mice. *Chinese Journal of Neurology* 48(7), 564-569.

Raab, A., Popp, S., Lesch, K.P., Lohse, M.J., Fischer, M., Deckert, J., et al. (2018). Increased fear learning, spatial learning as well as neophobia in Rgs2−/− mice: Increased fear learning spatial learning and neophobia in Rgs2$^{-/-}$ mice. *Genes, Brain and Behavior* 17(4), e12420. doi: 10.1111/gbb.12420.

Radlicka, A., Jabłońska, J., Lenarczyk, M., Szumiec, Ł., Harda, Z., Bagińska, M., et al. (2023). Nonmotor symptoms associated with progressive loss of dopaminergic neurons in a mouse model of Parkinson's disease. *BioRxiv*. doi: 10.1101/2023.01.23.525182.

Radwańska, K., and Kaczmarek, L. (2012). Characterization of an alcohol addiction-prone phenotype in mice: Characterization of an alcohol addiction-prone phenotype. *Addiction Biology* 17(3), 601-612. doi: 10.1111/j.1369-1600.2011.00394.x.

Radwańska, K, Pagano R, Salamian A, Skonieczna E, Wojtas B, Gielniewski B, Harda Z, Cały A, Havekes R, Abel T. (2023). Molecular fingerprints in the hippocampus of alcohol seeking during withdrawal. Res. Sq. 2023, rs.3.rs-3337670. doi: 10.21203/rs.3.rs-3337670/v1.

Ramakers, G.J.A., Wolfer, D., Rosenberger, G., Kuchenbecker, K., Kreienkamp, H.-J., Prange-Kiel, J., et al. (2012). Dysregulation of Rho GTPases in the αPix/Arhgef6 mouse model of X-linked intellectual disability is paralleled by impaired structural and synaptic plasticity and cognitive deficits. *Human Molecular Genetics* 21(2), 268-286. doi: 10.1093/hmg/ddr457.

Rankovic, V., Vogl, C., Dörje, N.M., Bahader, I., Duque-Afonso, C.J., Thirumalai, A., et al. (2021). Overloaded Adeno-Associated Virus as a Novel Gene Therapeutic Tool for Otoferlin-Related Deafness. *Frontiers in Molecular Neuroscience* 13, 600051. doi: 10.3389/fnmol.2020.600051.

Ratner, C., Skov, L.J., Raida, Z., Bächler, T., Bellmann-Sickert, K., Le Foll, C., et al. (2016). Effects of Peripheral Neurotensin on Appetite Regulation and Its Role in Gastric Bypass Surgery. *Endocrinology* 157(9), 3482-3492. doi: 10.1210/en.2016-1329.

Richter, S.H. (2020). Automated Home-Cage Testing as a Tool to Improve Reproducibility of Behavioral Research? *Frontiers in Neuroscience* 14, 383. doi: 10.3389/fnins.2020.00383.

Rivero, O., Alhama-Riba, J., Ku, H.-P., Fischer, M., Ortega, G., Álmos, P., et al. (2021). Haploinsufficiency of the Attention-Deficit/Hyperactivity Disorder Risk Gene St3gal3 in Mice Causes Alterations in Cognition and Expression of Genes Involved in Myelination and Sialylation. *Frontiers in Genetics* 12, 688488. doi: 10.3389/fgene.2021.688488.

Robinson, L., and Riedel, G. (2014). Comparison of automated home-cage monitoring systems: Emphasis on feeding behaviour, activity and spatial learning following pharmacological interventions. *Journal of Neuroscience Methods* 234, 13-25. doi: 10.1016/j.jneumeth.2014.06.013.

Roccaro-Waldmeyer, D.M., Girard, F., Milani, D., Vannoni, E., Prétôt, L., Wolfer, D.P., et al. (2018). Eliminating the VGlut2-Dependent Glutamatergic Transmission of Parvalbumin-Expressing Neurons Leads to Deficits in Locomotion and Vocalization, Decreased Pain Sensitivity, and Increased Dominance. *Frontiers in Behavioral Neuroscience* 12. doi: 10.3389/fnbeh.2018.00146.

Roughton, K., Kalm, M., and Blomgren, K. (2012). Sex-dependent differences in behavior and hippocampal neurogenesis after irradiation to the young mouse brain: Gender differences after IR to the rodent brain. *European Journal of Neuroscience* 36(6)**,** 2763-2772. doi: 10.1111/j.1460-9568.2012.08197.x.

Rudenko, O., Springer, C., Skov, L.J., Madsen, A.N., Hasholt, L., Nørremølle, A., et al. (2019). Ghrelin-mediated improvements in the metabolic phenotype in the R6/2 mouse model of Huntington's disease. *Journal of Neuroendocrinology* 31(7). doi: 10.1111/jne.12699.

Rudenko, O., Tkach, V., Berezin, V., and Bock, E. (2009). Detection of early behavioral markers of Huntington's disease in R6/2 mice employing an automated social home cage. *Behavioural Brain Research* 203(2)**,** 188-199. doi: 10.1016/j.bbr.2009.04.034.

Ruffini, N., Müller, M.B., Schmitt, U., and Gerber, S. (2021). IntelliPy: a GUI for analyzing IntelliCage data. *Bioinformatics* 37(21)**,** 3972-3973. doi: 10.1093/bioinformatics/btab682.

Ruud, J., Alber, J., Tokarska, A., Engström Ruud, L., Nolte, H., Biglari, N., et al. (2019). The Fat Mass and Obesity-Associated Protein (FTO) Regulates Locomotor Responses to Novelty via D2R Medium Spiny Neurons. *Cell Reports* 27(11)**,** 3182-3198.e3189. doi: 10.1016/j.celrep.2019.05.037.

Ryan, D., Koss, D., Porcu, E., Woodcock, H., Robinson, L., Platt, B., et al. (2013). Spatial learning impairments in PLB1Triple knock-in Alzheimer mice are task-specific and age-dependent. *Cellular and Molecular Life Sciences* 70(14)**,** 2603-2619. doi: 10.1007/s00018-013-1314-4.

Safi, K., Neuhäusser-Wespy, F., Welzl, H., and Lipp, H.-P. (2006). Mouse Anxiety Models and an Example of an Experimental Setup Using Unconditioned Avoidance in an Automated System - Intellicage. *Cognition Brain & Behavior***,** 15.

Sano, K., Isobe, T., Yang, J., Win-Shwe, T.-T., Yoshikane, M., Nakayama, S.F., et al. (2016). In utero and Lactational Exposure to Acetamiprid Induces Abnormalities in Socio-Sexual and Anxiety-Related Behaviors of Male Mice. *Frontiers in Neuroscience* 10. doi: 10.3389/fnins.2016.00228.

Sasaki, T., Saito, H., Furukawa, Y., Tominaga, T., Kitajima, S., Kanno, J., et al. (2023). Exposure to bisphenol A or its phenolic analogs during early life induces different types of anxiety-like behaviors after maturity in male mice. *The Journal of Toxicological Sciences* 48(4)**,** 211-219. doi: 10.2131/jts.48.211.

Sato, Y., Shinjyo, N., Sato, M., Nilsson, M.K.L., Osato, K., Zhu, C., et al. (2018). Grafting Neural Stem and Progenitor Cells Into the Hippocampus of Juvenile, Irradiated Mice Normalizes Behavior Deficits. *Frontiers in Neurology* 9. doi: 10.3389/fneur.2018.00715.

Schacke, S., Kirkpatrick, J., Stocksdale, A., Bauer, R., Hagel, C., Riecken, L.B., et al. (2022). Ezrin deficiency triggers glial fibrillary acidic protein upregulation and a distinct reactive astrocyte phenotype. *Glia* 70(12)**,** 2309-2329. doi: 10.1002/glia.24253.

Schroeder, S., Hofer, S.J., Zimmermann, A., Pechlaner, R., Dammbrueck, C., Pendl, T., et al. (2021). Dietary spermidine improves cognitive function. *Cell Reports* 35(2)**,** 108985. doi: 10.1016/j.celrep.2021.108985.

Schuler, B., Vogel, J., Grenacher, B., Jacobs, R.A., Arras, M., and Gassmann, M. (2012). Acute and chronic elevation of erythropoietin in the brain improves exercise performance in mice without inducing erythropoiesis. *The FASEB Journal* 26(9)**,** 3884-3890. doi: 10.1096/fj.11-191197.

Schumann, L., Wilken-Schmitz, A., Trautmann, S., Vogel, A., Schreiber, Y., Hahnefeld, L., et al. (2021). Increased Fat Taste Preference in Progranulin-Deficient Mice. *Nutrients* 13(11)**,** 4125. doi: 10.3390/nu13114125.

Sekiguchi, K., Imamura, S., Yamaguchi, T., Tabuchi, M., Kanno, H., Terawaki, K., et al. (2011). Effects of yokukansan and donepezil on learning disturbance and aggressiveness induced by intracerebroventricular injection of amyloid β protein in mice. *Phytotherapy Research* 25(4)**,** 501-507. doi: 10.1002/ptr.3287.

Serchov, T., Schwarz, I., Theiss, A., Sun, L., Holz, A., Döbrössy, M.D., et al. (2020). Enhanced adenosine A1 receptor and Homer1a expression in hippocampus modulates the resilience to stress-induced depression-like behavior. *Neuropharmacology* 162**,** 107834. doi: 10.1016/j.neuropharm.2019.107834.

Serykh, A., Khrapova, M.V., Dubrovina, N.I., Petrova, E.S., Mikhnevich, N., Starostina, M.V., et al. (2020). The increased density of the habenular neurons, high impulsivity, aggression and resistant fear memory in Disc1-Q31L genetic mouse model of depression. *Behavioural Brain Research* 392**,** 112693. doi: 10.1016/j.bbr.2020.112693.

Shemesh, Y., and Chen, A. (2023). A paradigm shift in translational psychiatry through rodent neuroethology. *Mol Psychiatry* 28(3)**,** 993-1003. doi: 10.1038/s41380-022-01913-z.

Shishelova, A.Y., Smirnov, K., and Raevskiĭ, V.V. (2022). Influence of early social isolation on general activity and spatial learning in adult WAG/Rij rats. *Developmental Psychobiology* 64(7). doi: 10.1002/dev.22319.

Shuaishuai, C., Lei, W., Tianjiao, X., Yin, C., Zhengliang, M., and Xiaoping, G. (2016). Effects of ambient temperature on spatial memory, weight and activity in mice. *International Journal of Anesthesiology and Resuscitation* 37(4)**,** 330-334.

Simmons, D.A., Belichenko, N.P., Ford, E.C., Semaan, S., Monbureau, M., Aiyaswamy, S., et al. (2016). A small molecule p75NTR ligand normalizes signalling and reduces Huntington's disease phenotypes in R6/2 and BACHD mice. *Human Molecular Genetics***,** ddw316. doi: 10.1093/hmg/ddw316.

Simmons, K.E., Healey, K.L., Li, Q., Moore, S.D., and Klein, R.C. (2021). Effects of sex and genotype in human *APOE*-targeted replacement mice on alcohol self-administration measured with the automated IntelliCage system before and after repeated mild traumatic brain injury. *Alcoholism: Clinical and Experimental Research* 45(11)**,** 2231-2245. doi: 10.1111/acer.14717.

Simmons, K.E., White, M.A., Healey, K.L., and Klein, R.C. (2021). Comparison of Enriched Housing Conditions on Automated Activity Monitoring and Behavior Following Repeated Mild Traumatic Brain Injury in Female C57BL/6 Mice. *SSRN Electronic Journal*. doi: 10.2139/ssrn.3899303.

Skupio, U., Sikora, M., Korostynski, M., Wawrzczak-Bargiela, A., Piechota, M., Ficek, J., et al. (2017). Behavioral and transcriptional patterns of protracted opioid self-administration in mice: Patterns of opioid addiction. *Addiction Biology* 22(6)**,** 1802-1816. doi: 10.1111/adb.12449.

Śliwińska, M.A., Cały, A., Borczyk, M., Ziółkowska, M., Skonieczna, E., Chilimoniuk, M., et al. (2020). Long-term Memory Upscales Volume of Postsynaptic Densities in the Process that Requires Autophosphorylation of αCaMKII. *Cerebral Cortex* 30(4)**,** 2573-2585. doi: 10.1093/cercor/bhz261.

Smutek, M., Turbasa, M., Sikora, M., Piechota, M., Zajdel, J., Przewlocki, R., et al. (2014). A Model of Alcohol Drinking under an Intermittent Access Schedule Using Group-Housed Mice. *PLoS ONE* 9(5)**,** e96787. doi: 10.1371/journal.pone.0096787.

Srimat Kandadai, K., Kotur, M.B., Dokalis, N., Amrein, I., Keller, C.W., Münz, C., et al. (2021). ATG5 in microglia does not contribute vitally to autoimmune neuroinflammation in mice. *Autophagy* 17(11)**,** 3566-3576. doi: 10.1080/15548627.2021.1883880.

Stefaniuk, M., Beroun, A., Lebitko, T., Markina, O., Leski, S., Meyza, K., et al. (2017). Matrix Metalloproteinase-9 and Synaptic Plasticity in the Central Amygdala in Control of Alcohol-Seeking Behavior. *Biological Psychiatry* 81(11)**,** 907-917. doi: 10.1016/j.biopsych.2016.12.026.

Stefaniuk, M., Pawlowska, M., Baranski, M., Nowicka, K., Zielinski, Z., Bijoch, L., et al. (2023). Global brain c-Fos profiling reveals major functional brain networks rearrangements after alcohol reexposure. *Neurobiol Dis* 178**,** 106006. doi: 10.1016/j.nbd.2023.106006.

Stefaniuk, M., Pawłowska, M., Nowicka, K., Barański, M., Zielinski, Z., Bijoch, Ł., et al. (2021). A whole-brain imaging-based systems approach to understand origin of addiction in binge-like drinking model. *bioRxiv*. doi: 10.1101/2021.02.17.431586.

Stephan, M., Schoeller, J., Raabe, F.J., Schmitt, A., Hasan, A., Falkai, P., et al. (2022). Spironolactone alleviates schizophrenia-related reversal learning in Tcf4 transgenic mice subjected to social defeat. *Schizophrenia* 8(1)**,** 77. doi: 10.1038/s41537-022-00290-4.

Stetter, C., Lopez-Caperuchipi, S., Hopp-Krämer, S., Bieber, M., Kleinschnitz, C., Sirén, A.-L., et al. (2021). Amelioration of Cognitive and Behavioral Deficits after Traumatic Brain Injury in Coagulation Factor XII Deficient Mice. *International Journal of Molecular Sciences* 22(9)**,** 4855. doi: 10.3390/ijms22094855.

Stribl, C., Samara, A., Trümbach, D., Peis, R., Neumann, M., Fuchs, H., et al. (2014). Mitochondrial Dysfunction and Decrease in Body Weight of a Transgenic Knock-in Mouse Model for TDP-43. *Journal of Biological Chemistry* 289(15)**,** 10769-10784. doi: 10.1074/jbc.M113.515940.

Sun, L., Verkaik-Schakel, R.-N., Biber, K., Plösch, T., and Serchov, T. (2021). Antidepressant treatment is associated with epigenetic alterations of Homer1 promoter in a mouse model of chronic depression. *Journal of Affective Disorders* 279**,** 501-509. doi: 10.1016/j.jad.2020.10.040.

Syding, L.A. (2022). *Mouse models for Angelman syndrome: generation and characterization.* PhD, Charles University Prague.

Syding, L.A., Kubik-Zahorodna, A., Nickl, P., Novosadova, V., Kopkanova, J., Kasparek, P., et al. (2022). Generation and Characterization of a Novel Angelman Syndrome Mouse Model with a Full Deletion of the Ube3a Gene. *Cells* 11(18)**,** 2815. doi: 10.3390/cells11182815.

Tegeder, I., Vogel, A., Ueberbach, T., Wilken-Schmitz, A., Jungenitz, T., Schmid, T., et al. (2022). Optogenetic early life pain leads to cortical hyperexcitability, nociceptive hypersensitivity and repetitive behavior. *Research Square*.

Tikhonova, M.A., Amstislavskaya, T.G., Ho, Y.-J., Akopyan, A.A., Tenditnik, M.V., Ovsyukova, M.V., et al. (2021). Neuroprotective Effects of Ceftriaxone Involve the Reduction of Aβ Burden and Neuroinflammatory Response in a Mouse Model of Alzheimer's Disease. *Frontiers in Neuroscience* 15**,** 736786. doi: 10.3389/fnins.2021.736786.

Too, L.K., Ball, H.J., McGregor, I.S., and Hunt, N.H. (2014). A novel automated test battery reveals enduring behavioural alterations and cognitive impairments in survivors of murine pneumococcal meningitis. *Brain, Behavior, and Immunity* 35**,** 107-124. doi: 10.1016/j.bbi.2013.09.007.

Too, L.K., Ball, H.J., McGregor, I.S., and Hunt, N.H. (2014). The pro-inflammatory cytokine interferon-gamma is an important driver of neuropathology and behavioural sequelae in experimental pneumococcal meningitis. *Brain, Behavior, and Immunity* 40**,** 252-268. doi: 10.1016/j.bbi.2014.02.020.

Too, L.K., Li, K.M., Suarna, C., Maghzal, G.J., Stocker, R., McGregor, I.S., et al. (2016). Deletion of TDO2, IDO-1 and IDO-2 differentially affects mouse behavior and cognitive function. *Behavioural Brain Research* 312**,** 102-117. doi: 10.1016/j.bbr.2016.06.018.

Too, L.K., Li, K.M., Suarna, C., Maghzal, G.J., Stocker, R., McGregor, I.S., et al. (2016). Behavioral and cognitive data in mice with different tryptophan-metabolizing enzymes knocked out. *Data in Brief* 9**,** 275-287. doi: 10.1016/j.dib.2016.08.071.

Too, L.K., McGregor, I.S., Baxter, A.G., and Hunt, N.H. (2016). Altered behaviour and cognitive function following combined deletion of Toll-like receptors 2 and 4 in mice. *Behavioural Brain Research* 303**,** 1-8. doi: 10.1016/j.bbr.2016.01.024.

Too, L.K., McQuillan, J.A., Ball, H.J., Kanai, M., Nakamura, T., Funakoshi, H., et al. (2014). The kynurenine pathway contributes to long-term neuropsychological changes in experimental pneumococcal meningitis. *Behavioural Brain Research* 270**,** 179-195. doi: 10.1016/j.bbr.2014.05.018.

Too, L.K., Mitchell, A.J., McGregor, I.S., and Hunt, N.H. (2016). Antibody-induced neutrophil depletion prior to the onset of pneumococcal meningitis influences long-term neurological complications in mice. *Brain, Behavior, and Immunity* 56**,** 68-83. doi: 10.1016/j.bbi.2016.01.021.

Too, L.K., Mitchell, A.J., Yau, B., Ball, H.J., McGregor, I.S., and Hunt, N.H. (2014). Interleukin-18 deficiency and its long-term behavioural and cognitive impacts in a murine model of pneumococcal meningitis. *Behavioural Brain Research* 263**,** 176-189. doi: 10.1016/j.bbr.2014.01.035.

Too, L.K., Yau, B., Baxter, A.G., McGregor, I.S., and Hunt, N.H. (2019). Double deficiency of toll-like receptors 2 and 4 alters long-term neurological sequelae in mice cured of pneumococcal meningitis. *Scientific Reports* 9(1)**,** 16189. doi: 10.1038/s41598-019-52212-7.

Tran, B.N., Valek, L., Wilken-Schmitz, A., Fuhrmann, D.C., Namgaladze, D., Wittig, I., et al. (2021). Reduced exploratory behavior in neuronal nucleoredoxin knockout mice. *Redox Biology* 45**,** 102054. doi: 10.1016/j.redox.2021.102054.

Ujita, W., Kohyama-Koganeya, A., Endo, N., Saito, T., and Oyama, H. (2018). Mice lacking a functional NMDA receptor exhibit social subordination in a group-housed environment. *The FEBS Journal* 285(1)**,** 188-196. doi: 10.1111/febs.14334.

Urbach, Y.K., Raber, K.A., Canneva, F., Plank, A.-C., Andreasson, T., Ponten, H., et al. (2014). Automated phenotyping and advanced data mining exemplified in rats transgenic for Huntington's disease. *Journal of Neuroscience Methods* 234**,** 38-53. doi: 10.1016/j.jneumeth.2014.06.017.

van Dijk, R.M., Lazic, S.E., Slomianka, L., Wolfer, D.P., and Amrein, I. (2016). Large-scale phenotyping links adult hippocampal neurogenesis to the reaction to novelty: Large scale phenotyping links neurogenesis to novelty. *Hippocampus* 26(5)**,** 646-657. doi: 10.1002/hipo.22548.

van Dijk, R.M., Wiget, F., Wolfer, D.P., Slomianka, L., and Amrein, I. (2019). Consistent within-group covariance of septal and temporal hippocampal neurogenesis with behavioral phenotypes for exploration and memory retention across wild and laboratory small rodents. *Behavioural Brain Research***,** 11.

Vannoni, E., Voikar, V., Colacicco, G., Sánchez, M.A., Lipp, H.-P., and Wolfer, D.P. (2014). Spontaneous behavior in the social homecage discriminates strains, lesions and mutations in mice. *Journal of Neuroscience Methods* 234**,** 26-37. doi: 10.1016/j.jneumeth.2014.04.026.

Vasić, V., Barth, K., Bicker, F., Schumann, U., Maurer, C., Heinig, N., et al. (2022). Less is more - loss of EGFL7 improves memory by upregulation of VEGF-D. *bioRxiv*. doi: 10.1101/2022.04.07.487327.

Vázquez, E., Barranco, A., Ramírez, M., Gruart, A., Delgado-García, J.M., Martínez-Lara, E., et al. (2015). Effects of a human milk oligosaccharide, 2′-fucosyllactose, on hippocampal long-term potentiation and learning capabilities in rodents. *The Journal of Nutritional Biochemistry* 26(5)**,** 455-465. doi: 10.1016/j.jnutbio.2014.11.016.

Viosca, J., Schuhmacher, A.J., Guerra, C., and Barco, A. (2009). Germline expression of H-RasG12V causes neurological deficits associated to Costello syndrome. *Genes, Brain and Behavior* 8(1)**,** 60-71. doi: 10.1111/j.1601-183X.2008.00443.x.

Vogel, A., Ueberbach, T., Wilken-Schmitz, A., Hahnefeld, L., Franck, L., Weyer, M.-P., et al. (2023). Repetitive and compulsive behavior after Early-Life-Pain associated with reduced long-chain sphingolipid species.

Vogel, A., Wilken-Schmitz, A., Hummel, R., Lang, M., Gurke, R., Schreiber, Y., et al. (2020). Low brain endocannabinoids associated with persistent non-goal directed nighttime hyperactivity after traumatic brain injury in mice. *Scientific Reports* 10(1)**,** 14929. doi: 10.1038/s41598-020-71879-x.

Voikar, V., Colacicco, G., Gruber, O., Vannoni, E., Lipp, H.-P., and Wolfer, D.P. (2010). Conditioned response suppression in the IntelliCage: assessment of mouse strain differences and effects of hippocampal and striatal lesions on acquisition and retention of memory. *Behavioural Brain Research* 213(2)**,** 304-312. doi: 10.1016/j.bbr.2010.05.019.

Voikar, V., and Gaburro, S. (2020). Three Pillars of Automated Home-Cage Phenotyping of Mice: Novel Findings, Refinement, and Reproducibility Based on Literature and Experience. *Frontiers in Behavioral Neuroscience* 14**,** 575434. doi: 10.3389/fnbeh.2020.575434.

Voikar, V., Krackow, S., Lipp, H.-P., Rau, A., Colacicco, G., and Wolfer, D.P. (2018). Automated dissection of permanent effects of hippocampal or prefrontal lesions on performance at spatial, working memory and circadian timing tasks of C57BL/6 mice in IntelliCage. *Behavioural Brain Research* 352**,** 8-22. doi: 10.1016/j.bbr.2017.08.048.

Voikar, V., Kulesskaya, N., Laakso, T., Lauren, J., Strittmatter, S.M., and Airaksinen, M.S. (2013). LRRTM1-deficient mice show a rare phenotype of avoiding small enclosures—A tentative mouse model for claustrophobia-like behaviour. *Behavioural Brain Research* 238**,** 69-78. doi: 10.1016/j.bbr.2012.10.013.

Volkmann, P., Stephan, M., Krackow, S., Jensen, N., and Rossner, M.J. (2021). PsyCoP – A Platform for Systematic Semi-Automated Behavioral and Cognitive Profiling Reveals Gene and Environment Dependent Impairments of Tcf4 Transgenic Mice Subjected to Social Defeat. *Frontiers in Behavioral Neuroscience* 14**,** 618180. doi: 10.3389/fnbeh.2020.618180.

Vyssotski, D.L. (2011). Heritable compensation of disturbed functionality. *Evolocus***,** 13.

Wahlsten, D. (2011). *Mouse Behavioral Testing. How to Use Mice in Behavioral Neuroscience. Chapter 11: How to motivate mice*. Academic Press, Elsevier.

Wang, L., Wang, F., Zhang, Z., Wu, N., and Sun, T. (2017). Effects of GABAB Receptor Expression in Rat Insular Cortex on the Ability of Spatial Exploration. *Journal of Ningxia Medical University* 39(4).

Welz, P.-S., Zinna, V.M., Symeonidi, A., Koronowski, K.B., Kinouchi, K., Smith, J.G., et al. (2019). BMAL1-Driven Tissue Clocks Respond Independently to Light to Maintain Homeostasis. *Cell* 177(6)**,** 1436-1447.e1412. doi: 10.1016/j.cell.2019.05.009.

Wen, Y., Ding, X., Guan, Q., Hu, W., Wang, B., Hu, Q., et al. (2021). Effects of exposure to urban particulate matter SRM 1648a during pregnancy on the neurobehavioral development of offspring mice. *Ecotoxicology and Environmental Safety* 215**,** 112142. doi: 10.1016/j.ecoenv.2021.112142.

Weyer, S.W., Klevanski, M., Delekate, A., Voikar, V., Aydin, D., Hick, M., et al. (2011). APP and APLP2 are essential at PNS and CNS synapses for transmission, spatial learning and LTP: APP and APLP2 synergize for synaptic function. *The EMBO Journal* 30(11)**,** 2266-2280. doi: 10.1038/emboj.2011.119.

Wilhelmsson, U., Kalm, M., Pekna, M., and Pekny, M. (2019). Nestin Null Mice Show Improved Reversal Place Learning. *Neurochemical Research*. doi: 10.1007/s11064-019-02854-w.

Wilhelmsson, U., Pozo-Rodrigalvarez, A., Kalm, M., de Pablo, Y., Widestrand, Å., Pekna, M., et al. (2019). The role of GFAP and vimentin in learning and memory. *Biological Chemistry***,** 10.

Wilke, J.B.H., Hindermann, M., Berghoff, S.A., Zihsler, S., Arinrad, S., Ronnenberg, A., et al. (2021). Autoantibodies against NMDA receptor 1 modify rather than cause encephalitis. *Molecular Psychiatry*. doi: 10.1038/s41380-021-01238-3.

Winiarski, M., Kondrakiewicz, L., Kondrakiewicz, K., Jędrzejewska-Szmek, J., Turzyński, K., Knapska, E., et al. (2022). Social deficits in BTBR T+ Itpr3tf/J mice vary with ecological validity of the test. *Genes, Brain and Behavior* 21(5). doi: 10.1111/gbb.12814.

Winslow, W., McDonough, I., Tallino, S., Decker, A., Vural, A.S., and Velazquez, R. (2021). IntelliCage Automated Behavioral Phenotyping Reveals Behavior Deficits in the 3xTg-AD Mouse Model of Alzheimer's Disease Associated With Brain Weight. *Frontiers in Aging Neuroscience* 13**,** 720214. doi: 10.3389/fnagi.2021.720214.

Wolfer, D., Võikar, V., Vannoni, E., Colacicco, G., and Lipp, H. (Year). "Mouse phenotyping in the IntelliCage: from spontaneous behavior to cognitive function", in: *Measuring Behavior*: Citeseer), 66.

Wu, N., Sun, T., Wu, X., Chen, H., and Zhang, Z. (2023). Modulation of GABAB receptors in the insula bidirectionally affects associative memory of epilectic rats in both spatial and non-spatial operant tasks. *Frontiers in Behavioral Neuroscience* 16**,** 1042227. doi: 10.3389/fnbeh.2022.1042227.

Wu, N., Wang, F., Jin, Z., Zhang, Z., Wang, L.-K., Zhang, C., et al. (2017). Effects of GABAB receptors in the insula on recognition memory observed with intellicage. *Behavioral and Brain Functions* 13(1). doi: 10.1186/s12993-017-0125-4.

Xia, T., Cui, Y., Chu, S., Song, J., Qian, Y., Ma, Z., et al. (2016). Melatonin pretreatment prevents isoflurane-induced cognitive dysfunction by modulating sleep–wake rhythm in mice. *Brain Research* 1634**,** 12-20. doi: 10.1016/j.brainres.2015.10.036.

Xiao, L., Jiang, S., Wang, Y., Gao, C., Liu, C., Huo, X., et al. (2022). Continuous high-frequency deep brain stimulation of the anterior insula modulates autism-like behavior in a valproic acid-induced rat model. *Journal of Translational Medicine* 20(1)**,** 570. doi: 10.1186/s12967-022-03787-9.

Xing, L., Kubik-Zahorodna, A., Namba, T., Pinson, A., Florio, M., Prochazka, J., et al. (2021). Expression of human-specific ARHGAP11B in mice leads to neocortex expansion and increased memory flexibility. *The EMBO Journal* 40(13). doi: 10.15252/embj.2020107093.

Yamamoto, H., Lee-Okada, H.C., Ikeda, M., Nakamura, T., Saito, T., Takata, A., et al. (2023). GWAS-identified bipolar disorder risk allele in the FADS1/2 gene region links mood episodes and unsaturated fatty acid metabolism in mutant mice. *Mol Psychiatry*. doi: 10.1038/s41380-023-01988-2.

Yang, X., Wang, F., Wu, N., Zhao, X., and Sun, T. (2018). Effects of GABAB2 Receptor Expression on the Impairment of Recognition Memory in Lithium Chloride-pilocarpine Epileptic Rat Insular Cortex. *Journal of Ningxia Medical University*.

Yang, Y., Chen, X., Min, H., Song, S., Zhang, J., Fan, S., et al. (2017). Persistent mitoKATP Activation Is Involved in the Isoflurane-induced Cytotoxicity. *Molecular Neurobiology* 54(2)**,** 1101-1110. doi: 10.1007/s12035-016-9710-z.

Yang, Y., Liu, Y., Zhu, J., Song, S., Huang, Y., Zhang, W., et al. (2022). Neuroinflammation-mediated mitochondrial dysregulation involved in postoperative cognitive dysfunction. *Free Radical Biology and Medicine* 178**,** 134-146. doi: 10.1016/j.freeradbiomed.2021.12.004.

Yang, Y., Sun, K., Liu, W., Li, X., Tian, W., Shuai, P., et al. (2021). The phosphatidylserine flippase β-subunit Tmem30a is essential for normal insulin maturation and secretion. *Molecular Therapy* 29(9)**,** 2854-2872. doi: 10.1016/j.ymthe.2021.04.026.

Yang, Y., Wang, H., Song, S., Liu, Y., Zhang, W., Zhang, J., et al. (2021). STAT3 Involved in Cellular Vulnerability to Isoflurane. *Research Square*.

Yesiltepe, M., Yin, T., Tambini, M.D., Breuillaud, L., Zehntner, S.P., and D'Adamio, L. (2022). Late-long-term potentiation magnitude, but not Aβ levels and amyloid pathology, is associated with behavioral performance in a rat knock-in model of Alzheimer disease. *Frontiers in Aging Neuroscience* 14**,** 1040576. doi: 10.3389/fnagi.2022.1040576.

Zhang, C., Liu, Q., Yu, C.-Y., Wang, F., Shao, Y., Sun, K.-S., et al. (2020). G Protein-Coupled Estrogen Receptor 1 Knockout Deteriorates MK-801-Induced Learning and Memory Impairment in Mice. *Frontiers in Behavioral Neuroscience* 14**,** 157. doi: 10.3389/fnbeh.2020.00157.

Zheng, J., Jiang, R., Chen, M., Maimaitiming, Z., Wang, J., Anderson, G.J., et al. (2018). Multi-Copper Ferroxidase–Deficient Mice Have Increased Brain Iron Concentrations and Learning and Memory Deficits. *The Journal of Nutrition* 148(4)**,** 643-649. doi: 10.1093/jn/nxy012.

Zheng, Y., Wu, M., Gao, T., Meng, L., Ding, X., Meng, Y., et al. (2020). GPER-Deficient Rats Exhibit Lower Serum Corticosterone Level and Increased Anxiety-Like Behavior. *Neural Plasticity* 2020**,** 1-22. doi: 10.1155/2020/8866187.

Zhu, C., Gao, J., Karlsson, N., Li, Q., Zhang, Y., Huang, Z., et al. (2010). Isoflurane Anesthesia Induced Persistent, Progressive Memory Impairment, Caused a Loss of Neural Stem Cells, and Reduced Neurogenesis in Young, but Not Adult, Rodents. *Journal of Cerebral Blood Flow & Metabolism* 30(5)**,** 1017-1030. doi: 10.1038/jcbfm.2009.274.

Zhu, S., Shi, J., Jin, Q., Zhang, Y., Zhang, R., Chen, X., Wang, C., Shi, T., and Li, L. (2023). Mitochondrial dysfunction following repeated administration of alprazolam causes attenuation of hippocampus-dependent memory consolidation in mice. Aging 15. doi: 10.18632/aging.205087.

All References for Intellicage 2005 – October 15 2023, chronological

Contains a few references of review papers (marked yellow)

2005

Galsworthy, M., Amrein, I., Kuptsov, P., Poletaeva, I., Zinn, P., Rau, A., et al. (2005). A comparison of wild-caught wood mice and bank voles in the Intellicage: assessing exploration, daily activity patterns and place learning paradigms. *Behavioural Brain Research* 157(2)**,** 211-217. doi: 10.1016/j.bbr.2004.06.021.

Lipp, H.-P. (2005). High-throughput and Automated Behavioural Screening of Normal and Genetically Modified Mice. *Business Briefing: Future Drug Discovery***,** 5.

Lipp, H.P., Galsworthy, M., Vyssotski, D.L., Zinn, P., Rau, A.E., Neuhäusser-Wespy, F., et al. (2005). Automated behavioral analysis of mice using INTELLICAGE: inter-laboratory comparisons and validation with exploratory behavior and spatial learning. *Proceedings of Measuring Behaviour 2005***,** 66-69.

2006

Knapska, E. (2006). Differential involvement of the central amygdala in appetitive versus aversive learning. *Learning & Memory* 13(2)**,** 192-200. doi: 10.1101/lm.54706.

Safi, K., Neuhäusser-Wespy, F., Welzl, H., and Lipp, H.-P. (2006). Mouse Anxiety Models and an Example of an Experimental Setup Using Unconditioned Avoidance in an Automated System - Intellicage. *Cognition Brain & Behavior***,** 15.

2007

Onishchenko, N., Tamm, C., Vahter, M., Hökfelt, T., Johnson, J.A., Johnson, D.A., et al. (2007). Developmental Exposure to Methylmercury Alters Learning and Induces Depression-like Behavior in Male Mice. *Toxicological Sciences* 97(2)**,** 428-437. doi: 10.1093/toxsci/kfl199.

2008

Kiryk, A., Aida, T., Tanaka, K., Banerjee, P., Wilczynski, G.M., Meyza, K., et al. (2008). Behavioral characterization of GLT1 (+/-) mice as a model of mild glutamatergic hyperfunction. *Neurotoxicity Research* 13(1)**,** 19-30. doi: 10.1007/BF03033364.

2009

Jaholkowski, P., Kiryk, A., Jedynak, P., Ben Abdallah, N.M., Knapska, E., Kowalczyk, A., et al. (2009). New hippocampal neurons are not obligatory for memory formation; cyclin D2 knockout mice with no adult brain neurogenesis show learning. *Learning & Memory* 16(7)**,** 439-451. doi: 10.1101/lm.1459709.

Mechan, A.O., Wyss, A., Rieger, H., and Mohajeri, M.H. (2009). A comparison of learning and memory characteristics of young and middle-aged wild-type mice in the IntelliCage. *Journal of Neuroscience Methods* 180(1)**,** 43-51. doi: 10.1016/j.jneumeth.2009.02.018.

Rudenko, O., Tkach, V., Berezin, V., and Bock, E. (2009). Detection of early behavioral markers of Huntington's disease in R6/2 mice employing an automated social home cage. *Behavioural Brain Research* 203(2)**,** 188-199. doi: 10.1016/j.bbr.2009.04.034.

Viosca, J., Schuhmacher, A.J., Guerra, C., and Barco, A. (2009). Germline expression of H-RasG12V causes neurological deficits associated to Costello syndrome. *Genes, Brain and Behavior* 8(1)**,** 60-71. doi: 10.1111/j.1601-183X.2008.00443.x.

2010

Barlind, A., Karlsson, N., Björk-Eriksson, T., Isgaard, J., and Blomgren, K. (2010). Decreased cytogenesis in the granule cell layer of the hippocampus and impaired place learning after irradiation of the young mouse brain evaluated using the IntelliCage platform. *Experimental Brain Research* 201(4)**,** 781-787. doi: 10.1007/s00221-009-2095-8.

Branchi, I., D'Andrea, I., Cirulli, F., Lipp, H.-P., and Alleva, E. (2010). Shaping brain development: Mouse communal nesting blunts adult neuroendocrine and behavioral response to social stress and modifies chronic antidepressant treatment outcome. *Psychoneuroendocrinology* 35(5)**,** 743-751. doi: 10.1016/j.psyneuen.2009.10.016.

Codita, A., Gumucio, A., Lannfelt, L., Gellerfors, P., Winblad, B., Mohammed, A.H., et al. (2010). Impaired behavior of female tg-ArcSwe APP mice in the IntelliCage: A longitudinal study. *Behavioural Brain Research* 215(1)**,** 83-94. doi: 10.1016/j.bbr.2010.06.034.

Konopka, W., Kiryk, A., Novak, M., Herwerth, M., Parkitna, J.R., Wawrzyniak, M., et al. (2010). MicroRNA Loss Enhances Learning and Memory in Mice. *Journal of Neuroscience* 30(44)**,** 14835-14842. doi: 10.1523/JNEUROSCI.3030-10.2010.

Krackow, S., Vannoni, E., Codita, A., Mohammed, A.H., Cirulli, F., Branchi, I., et al. (2010). Consistent behavioral phenotype differences between inbred mouse strains in the IntelliCage. *Genes, Brain and Behavior* 9(7)**,** 722-731. doi: 10.1111/j.1601-183X.2010.00606.x.

Voikar, V., Colacicco, G., Gruber, O., Vannoni, E., Lipp, H.-P., and Wolfer, D.P. (2010). Conditioned response suppression in the IntelliCage: assessment of mouse strain differences and effects of hippocampal and striatal lesions on acquisition and retention of memory. *Behavioural Brain Research* 213(2)**,** 304-312. doi: 10.1016/j.bbr.2010.05.019.

Zhu, C., Gao, J., Karlsson, N., Li, Q., Zhang, Y., Huang, Z., et al. (2010). Isoflurane Anesthesia Induced Persistent, Progressive Memory Impairment, Caused a Loss of Neural Stem Cells, and Reduced Neurogenesis in Young, but Not Adult, Rodents. *Journal of Cerebral Blood Flow & Metabolism* 30(5)**,** 1017-1030. doi: 10.1038/jcbfm.2009.274.

2011

d'Isa, R., Clapcote, S. J., Voikar, V., Wolfer, D. P., Giese, K. P., Brambilla, R., and Fasano, S. (2011). Mice Lacking Ras-GRF1 Show Contextual Fear Conditioning but not Spatial Memory Impairments: Convergent Evidence from Two Independently Generated Mouse Mutant Lines. Front. Behav. Neurosci. 5, 78. doi: 10.3389/fnbeh.2011.00078.

Endo, T., Maekawa, F., Võikar, V., Haijima, A., Uemura, Y., Zhang, Y., et al. (2011). Automated test of behavioral flexibility in mice using a behavioral sequencing task in IntelliCage. *Behavioural Brain Research* 221(1)**,** 172-181. doi: 10.1016/j.bbr.2011.02.037.

Ermakova, O., Piszczek, L., Luciani, L., Cavalli, F.M.G., Ferreira, T., Farley, D., et al. (2011). Sensitized phenotypic screening identifies gene dosage sensitive region on chromosome 11 that predisposes to disease in mice: Phenotyping of Df11(1) and Dp11(1) mouse lines. *EMBO Molecular Medicine* 3(1)**,** 50-66. doi: 10.1002/emmm.201000112.

Faizi, M., Bader, P.L., Tun, C., Encarnacion, A., Kleschevnikov, A., Belichenko, P., et al. (2011). Comprehensive behavioral phenotyping of Ts65Dn mouse model of Down Syndrome: Activation of β1-adrenergic receptor by xamoterol as a potential cognitive enhancer. *Neurobiology of Disease* 43(2)**,** 397-413. doi: 10.1016/j.nbd.2011.04.011.

Karlsson, N., Kalm, M., Nilsson, M.K.L., Mallard, C., Björk-Eriksson, T., and Blomgren, K. (2011). Learning and Activity after Irradiation of the Young Mouse Brain Analyzed in Adulthood Using Unbiased Monitoring in a Home Cage Environment. *Radiation Research* 175(3)**,** 336-346. doi: 10.1667/RR2231.1.

Kiryk, A., Mochol, G., K. Filipkowski, R., Wawrzyniak, M., Lioudyno, V., Knapska, E., et al. (2011). Cognitive Abilities of Alzheimers Disease Transgenic Mice are Modulated by Social Context and Circadian Rhythm. *Current Alzheimer Research* 8(8)**,** 883-892. doi: 10.2174/156720511798192745.

Lan, W.-C.J., Priestley, M., Mayoral, S.R., Tian, L., Shamloo, M., and Penn, A.A. (2011). Sex-Specific Cognitive Deficits and Regional Brain Volume Loss in Mice Exposed to Chronic, Sublethal Hypoxia. *Pediatric Research* 70(1)**,** 15-20. doi: 10.1203/PDR.0b013e31821b98a3.

Oakeshott, S., Balci, F., Filippov, I., Murphy, C., Port, R., Connor, D., et al. (2011). Circadian Abnormalities in Motor Activity in a BAC Transgenic Mouse Model of Huntington's Disease. *PLoS Currents* 3**,** RRN1225. doi: 10.1371/currents.RRN1225.

Sekiguchi, K., Imamura, S., Yamaguchi, T., Tabuchi, M., Kanno, H., Terawaki, K., et al. (2011). Effects of yokukansan and donepezil on learning disturbance and aggressiveness induced by intracerebroventricular injection of amyloid β protein in mice. *Phytotherapy Research* 25(4)**,** 501-507. doi: 10.1002/ptr.3287.

Vyssotski, D.L. (2011). Heritable compensation of disturbed functionality. *Evolocus***,** 13.

Wahlsten, D. (2011). Mouse Behavioral Testing. How to Use Mice in Behavioral Neuroscience. Chapter 11. How to motivate mice. Academic Press, Elsevier, ISBN 9780123756756.

Weyer, S.W., Klevanski, M., Delekate, A., Voikar, V., Aydin, D., Hick, M., et al. (2011). APP and APLP2 are essential at PNS and CNS synapses for transmission, spatial learning and LTP: APP and APLP2 synergize for synaptic function. *The EMBO Journal* 30(11)**,** 2266-2280. doi: 10.1038/emboj.2011.119.

2012

Berry, A., Amrein, I., Nötzli, S., Lazic, S.E., Bellisario, V., Giorgio, M., et al. (2012). Sustained hippocampal neurogenesis in females is amplified in P66Shc−/− mice: An animal model of healthy aging. *Hippocampus* 22(12)**,** 2249-2259. doi: 10.1002/hipo.22042.

Codita, A., Mohammed, A.H., Willuweit, A., Reichelt, A., Alleva, E., Branchi, I., et al. (2012). Effects of Spatial and Cognitive Enrichment on Activity Pattern and Learning Performance in Three Strains of Mice in the IntelliMaze. *Behavior Genetics* 42(3)**,** 449-460. doi: 10.1007/s10519-011-9512-z.

Endo, T., Kakeyama, M., Uemura, Y., Haijima, A., Okuno, H., Bito, H., et al. (2012). Executive Function Deficits and Social-Behavioral Abnormality in Mice Exposed to a Low Dose of Dioxin In Utero and via Lactation. *PLoS ONE* 7(12)**,** e50741. doi: 10.1371/journal.pone.0050741.

Huo, K., Sun, Y., Li, H., Du, X., Wang, X., Karlsson, N., et al. (2012). Lithium reduced neural progenitor apoptosis in the hippocampus and ameliorated functional deficits after irradiation to the immature mouse brain. *Molecular and Cellular Neuroscience* 51(1-2)**,** 32-42. doi: 10.1016/j.mcn.2012.07.002.

Jedynak, P., Jaholkowski, P., Wozniak, G., Sandi, C., Kaczmarek, L., and Filipkowski, R.K. (2012). Lack of cyclin D2 impairing adult brain neurogenesis alters hippocampal-dependent behavioral tasks without reducing learning ability. *Behavioural Brain Research* 227(1)**,** 159-166. doi: 10.1016/j.bbr.2011.11.007.

Menalled, L.B., Kudwa, A.E., Miller, S., Fitzpatrick, J., Watson-Johnson, J., Keating, N., et al. (2012). Comprehensive Behavioral and Molecular Characterization of a New Knock-In Mouse Model of Huntington's Disease: zQ175. *PLoS ONE* 7(12)**,** e49838. doi: 10.1371/journal.pone.0049838.

Muthuraju, S., Pati, S., Rafiqul, M., Abdullah, J.M., and Jaafar, H. (2012). IntelliCage provides voluntary exercise and an enriched environment, improving locomotive activity in mice following fluid percussion injury. *Basal Ganglia* 2(3)**,** 143-151. doi: 10.1016/j.baga.2012.06.004.

Oakeshott, S., Port, R., Cummins-Sutphen, J., Berger, J., Watson-Johnson, J., Ramboz, S., et al. (2012). A mixed fixed ratio/progressive ratio procedure reveals an apathy phenotype in the BAC HD and the z_Q175 KI mouse models of Huntington's disease. *PLoS Currents*. doi: 10.1371/4f972cffe82c0.

Piechota, M., Korostynski, M., Sikora, M., Golda, S., Dzbek, J., and Przewlocki, R. (2012). Common transcriptional effects in the mouse striatum following chronic treatment with heroin and methamphetamine. *Genes, Brain and Behavior* 11(4)**,** 404-414. doi: 10.1111/j.1601-183X.2012.00777.x.

Radwanska, K., and Kaczmarek, L. (2012). Characterization of an alcohol addiction-prone phenotype in mice: Characterization of an alcohol addiction-prone phenotype. *Addiction Biology* 17(3)**,** 601-612. doi: 10.1111/j.1369-1600.2011.00394.x.

Ramakers, G.J.A., Wolfer, D., Rosenberger, G., Kuchenbecker, K., Kreienkamp, H.-J., Prange-Kiel, J., et al. (2012). Dysregulation of Rho GTPases in the αPix/Arhgef6 mouse model of X-linked intellectual disability is paralleled by impaired structural and synaptic plasticity and cognitive deficits. *Human Molecular Genetics* 21(2)**,** 268-286. doi: 10.1093/hmg/ddr457.

Roughton, K., Kalm, M., and Blomgren, K. (2012). Sex-dependent differences in behavior and hippocampal neurogenesis after irradiation to the young mouse brain: Gender differences after IR to the rodent brain. *European Journal of Neuroscience* 36(6)**,** 2763-2772. doi: 10.1111/j.1460-9568.2012.08197.x.

Schuler, B., Vogel, J., Grenacher, B., Jacobs, R.A., Arras, M., and Gassmann, M. (2012). Acute and chronic elevation of erythropoietin in the brain improves exercise performance in mice without inducing erythropoiesis. *The FASEB Journal* 26(9)**,** 3884-3890. doi: 10.1096/fj.11-191197.

Wolfer, D., Võikar, V., Vannoni, E., Colacicco, G., and Lipp, H. (Year). "Mouse phenotyping in the IntelliCage: from spontaneous behavior to cognitive function", in: *Measuring Behavior*: Citeseer), 66.

2013

Albuquerque, B., Häussler, A., Vannoni, E., Wolfer, D.P., and Tegeder, I. (2013). Learning and memory with neuropathic pain: impact of old age and progranulin deficiency. *Frontiers in Behavioral Neuroscience* 7. doi: 10.3389/fnbeh.2013.00174.

Balci, F., Shamy, J.L., El-Khodor, B.F., Filippov, I., Mushlin, R., Port, R., et al. (2013). High-Throughput Automated Phenotyping of Two Genetic Mouse Models of Huntington's Disease. *PLoS Currents*. doi: 10.1371/currents.hd.124aa0d16753f88215776fba102ceb29.

Ben Abdallah, N.M.B., Filipkowski, R.K., Pruschy, M., Jaholkowski, P., Winkler, J., Kaczmarek, L., et al. (2013). Impaired long-term memory retention: Common denominator for acutely or genetically reduced hippocampal neurogenesis in adult mice. *Behavioural Brain Research* 252**,** 275-286. doi: 10.1016/j.bbr.2013.05.034.

Branchi, I., Santarelli, S., Capoccia, S., Poggini, S., D'Andrea, I., Cirulli, F., et al. (2013). Antidepressant Treatment Outcome Depends on the Quality of the Living Environment: A Pre-Clinical Investigation in Mice. *PLoS ONE* 8(4)**,** e62226. doi: 10.1371/journal.pone.0062226.

Branchi, I., Santarelli, S., D'Andrea, I., and Alleva, E. (2013). Not all stressors are equal: Early social enrichment favors resilience to social but not physical stress in male mice. *Hormones and Behavior* 63(3)**,** 503-509. doi: 10.1016/j.yhbeh.2013.01.003.

Gumucio, A., Lannfelt, L., and Nilsson, L.N.G. (2013). Lack of exon 10 in the murine tau gene results in mild sensorimotor defects with aging. *BMC Neuroscience* 14(1), 148. doi: 10.1186/1471-2202-14-148.

Kalm, M., Karlsson, N., Nilsson, M.K.L., and Blomgren, K. (2013). Loss of hippocampal neurogenesis, increased novelty-induced activity, decreased home cage activity, and impaired reversal learning one year after irradiation of the young mouse brain. *Experimental Neurology* 247, 402-409. doi: 10.1016/j.expneurol.2013.01.006.

Knapska, E., Lioudyno, V., Kiryk, A., Mikosz, M., Gorkiewicz, T., Michaluk, P., et al. (2013). Reward Learning Requires Activity of Matrix Metalloproteinase-9 in the Central Amygdala. *Journal of Neuroscience* 33(36), 14591-14600. doi: 10.1523/JNEUROSCI.5239-12.2013.

Kobayashi, Y., Sano, Y., Vannoni, E., Goto, H., Suzuki, H., Oba, A., et al. (2013). Genetic dissection of medial habenula–interpeduncular nucleus pathway function in mice. *Frontiers in Behavioral Neuroscience* 7. doi: 10.3389/fnbeh.2013.00017.

Kulesskaya, N., Võikar, V., Peltola, M., Yegutkin, G.G., Salmi, M., Jalkanen, S., et al. (2013). CD73 Is a Major Regulator of Adenosinergic Signalling in Mouse Brain. *PLoS ONE* 8(6), e66896. doi: 10.1371/journal.pone.0066896.

Muthuraju, S., Taha, S., Pati, S., Rafique, M., Jaafar, H., and Abdullah, J.M. (2013). Normabaric Hyperoxia Treatment Improved Locomotor Activity of C57BL/6J Mice through Enhancing Dopamine Genes Following Fluid-Percussion Injury in Striatum. *International Journal of Biomedical Science* 9(4), 11.

Nowak, A., Werka, T., and Knapska, E. (2013). Social modulation in extinction of aversive memories. *Behavioural Brain Research* 238, 200-205. doi: 10.1016/j.bbr.2012.10.031.

Oakeshott, S., Farrar, A., Port, R., Cummins-Sutphen, J., Berger, J., Watson-Johnson, J., et al. (2013). Deficits in a Simple Visual Go/No-go Discrimination Task in Two Mouse Models of Huntington's Disease. *PLoS Currents*. doi: 10.1371/currents.hd.fe74c94bdd446a0470f6f905a30b5dd1.

Ogi, H., Itoh, K., and Fushiki, S. (2013). Social behavior is perturbed in mice after exposure to bisphenol A: a novel assessment employing an IntelliCage. *Brain and Behavior* 3(3), 223-228. doi: 10.1002/brb3.130.

Parkitna, J.R., Sikora, M., Gołda, S., Gołembiowska, K., Bystrowska, B., Engblom, D., et al. (2013). Novelty-Seeking Behaviors and the Escalation of Alcohol Drinking After Abstinence in Mice Are Controlled by Metabotropic Glutamate Receptor 5 on Neurons Expressing Dopamine D1 Receptors. *Biological Psychiatry* 73(3), 263-270. doi: 10.1016/j.biopsych.2012.07.019.

Ryan, D., Koss, D., Porcu, E., Woodcock, H., Robinson, L., Platt, B., et al. (2013). Spatial learning impairments in PLB1Triple knock-in Alzheimer mice are task-specific and age-dependent. *Cellular and Molecular Life Sciences* 70(14), 2603-2619. doi: 10.1007/s00018-013-1314-4.

Voikar, V., Kulesskaya, N., Laakso, T., Lauren, J., Strittmatter, S.M., and Airaksinen, M.S. (2013). LRRTM1-deficient mice show a rare phenotype of avoiding small enclosures—A tentative mouse model for claustrophobia-like behaviour. *Behavioural Brain Research* 238, 69-78. doi: 10.1016/j.bbr.2012.10.013.

2014

Benner, S., Endo, T., Endo, N., Kakeyama, M., and Tohyama, C. (2014). Early deprivation induces competitive subordinance in C57BL/6 male mice. *Physiology & Behavior* 137, 42-52. doi: 10.1016/j.physbeh.2014.06.018.

de Hoz, L., and Nelken, I. (2014). Frequency Tuning in the Behaving Mouse: Different Bandwidths for Discrimination and Generalization. *PLoS ONE* 9(3), e91676. doi: 10.1371/journal.pone.0091676.

Gapp, K., Soldado-Magraner, S., Alvarez-Sánchez, M., Bohacek, J., Vernaz, G., Shu, H., et al. (2014). Early life stress in fathers improves behavioural flexibility in their offspring. *Nature Communications* 5(1). doi: 10.1038/ncomms6466.

Iwata, R., Ohi, K., Kobayashi, Y., Masuda, A., Iwama, M., Yasuda, Y., et al. (2014). RacGAP α2-Chimaerin Function in Development Adjusts Cognitive Ability in Adulthood. *Cell Reports* 8(5)**,** 1257-1264. doi: 10.1016/j.celrep.2014.07.047.

Kulesskaya, N., Karpova, N.N., Ma, L., Tian, L., and Voikar, V. (2014). Mixed housing with DBA/2 mice induces stress in C57BL/6 mice: implications for interventions based on social enrichment. *Frontiers in Behavioral Neuroscience* 8. doi: 10.3389/fnbeh.2014.00257.

Menalled, L.B., Kudwa, A.E., Oakeshott, S., Farrar, A., Paterson, N., Filippov, I., et al. (2014). Genetic Deletion of Transglutaminase 2 Does Not Rescue the Phenotypic Deficits Observed in R6/2 and zQ175 Mouse Models of Huntington's Disease. *PLoS ONE* 9(6)**,** e99520. doi: 10.1371/journal.pone.0099520.

Osman, A.M., Zhou, K., Zhu, C., and Blomgren, K. (2014). Transplantation of Enteric Neural Stem/Progenitor Cells into the Irradiated Young Mouse Hippocampus. *Cell Transplantation* 23(12)**,** 1657-1671. doi: 10.3727/096368913X674648.

Patrikainen, M., Pan, P., Kulesskaya, N., Voikar, V., and Parkkila, S. (2014). The role of carbonic anhydrase VI in bitter taste perception: evidence from the Car6 −/− mouse model. *Journal of Biomedical Science* 21(1). doi: 10.1186/s12929-014-0082-2.

Perez-Alcazar, M., Daborg, J., Stokowska, A., Wasling, P., Björefeldt, A., Kalm, M., et al. (2014). Altered cognitive performance and synaptic function in the hippocampus of mice lacking C3. *Experimental Neurology* 253**,** 154-164. doi: 10.1016/j.expneurol.2013.12.013.

Puścian, A., Łęski, S., Gorkiewicz, T., Meyza, K., Lipp, H.-P., and Knapska, E. (2014). A novel automated behavioral test battery assessing cognitive rigidity in two genetic mouse models of autism. *Frontiers in Behavioral Neuroscience* 8. doi: 10.3389/fnbeh.2014.00140.

Robinson, L., and Riedel, G. (2014). Comparison of automated home-cage monitoring systems: Emphasis on feeding behaviour, activity and spatial learning following pharmacological interventions. *Journal of Neuroscience Methods* 234**,** 13-25. doi: 10.1016/j.jneumeth.2014.06.013.

Smutek, M., Turbasa, M., Sikora, M., Piechota, M., Zajdel, J., Przewlocki, R., et al. (2014). A Model of Alcohol Drinking under an Intermittent Access Schedule Using Group-Housed Mice. *PLoS ONE* 9(5)**,** e96787. doi: 10.1371/journal.pone.0096787.

Stribl, C., Samara, A., Trümbach, D., Peis, R., Neumann, M., Fuchs, H., et al. (2014). Mitochondrial Dysfunction and Decrease in Body Weight of a Transgenic Knock-in Mouse Model for TDP-43. *Journal of Biological Chemistry* 289(15)**,** 10769-10784. doi: 10.1074/jbc.M113.515940.

Too, L.K., Ball, H.J., McGregor, I.S., and Hunt, N.H. (2014). A novel automated test battery reveals enduring behavioural alterations and cognitive impairments in survivors of murine pneumococcal meningitis. *Brain, Behavior, and Immunity* 35**,** 107-124. doi: 10.1016/j.bbi.2013.09.007.

Too, L.K., Ball, H.J., McGregor, I.S., and Hunt, N.H. (2014). The pro-inflammatory cytokine interferon-gamma is an important driver of neuropathology and behavioural sequelae in experimental pneumococcal meningitis. *Brain, Behavior, and Immunity* 40**,** 252-268. doi: 10.1016/j.bbi.2014.02.020.

Too, L.K., McQuillan, J.A., Ball, H.J., Kanai, M., Nakamura, T., Funakoshi, H., et al. (2014). The kynurenine pathway contributes to long-term neuropsychological changes in experimental pneumococcal meningitis. *Behavioural Brain Research* 270**,** 179-195. doi: 10.1016/j.bbr.2014.05.018.

Too, L.K., Mitchell, A.J., Yau, B., Ball, H.J., McGregor, I.S., and Hunt, N.H. (2014). Interleukin-18 deficiency and its long-term behavioural and cognitive impacts in a murine model of pneumococcal meningitis. *Behavioural Brain Research* 263**,** 176-189. doi: 10.1016/j.bbr.2014.01.035.

Urbach, Y.K., Raber, K.A., Canneva, F., Plank, A.-C., Andreasson, T., Ponten, H., et al. (2014). Automated phenotyping and advanced data mining exemplified in rats transgenic for Huntington's disease. *Journal of Neuroscience Methods* 234**,** 38-53. doi: 10.1016/j.jneumeth.2014.06.017.

Vannoni, E., Voikar, V., Colacicco, G., Sánchez, M.A., Lipp, H.-P., and Wolfer, D.P. (2014). Spontaneous behavior in the social homecage discriminates strains, lesions and mutations in mice. *Journal of Neuroscience Methods* 234**,** 26-37. doi: 10.1016/j.jneumeth.2014.04.026.

2015

Alexandrov, V., Brunner, D., Hanania, T., and Leahy, E. (2015). Highthroughtput analysis of behavior for drug discovery. *European Journal of Pharmacology* 753**,** 127-134. doi: 10.1016/j.ejphar.2015.02.037.

Benner, S., Endo, T., Kakeyama, M., and Tohyama, C. (2015). Environmental insults in early life and submissiveness later in life in mouse models. *Frontiers in Neuroscience* 9. doi: 10.3389/fnins.2015.00091.

Cathomas, F., Fuertig, R., Sigrist, H., Newman, G.N., Hoop, V., Bizzozzero, M., et al. (2015). CD40-TNF activation in mice induces extended sickness behavior syndrome co-incident with but not dependent on activation of the kynurenine pathway. *Brain, Behavior, and Immunity* 50**,** 125-140. doi: 10.1016/j.bbi.2015.06.184.

Cathomas, F., Stegen, M., Sigrist, H., Schmid, L., Seifritz, E., Gassmann, M., et al. (2015). Altered emotionality and neuronal excitability in mice lacking KCTD12, an auxiliary subunit of GABAB receptors associated with mood disorders. *Translational Psychiatry* 5(2)**,** e510-e510. doi: 10.1038/tp.2015.8.

Hölter, S.M., Garrett, L., Einicke, J., Sperling, B., Dirscherl, P., Zimprich, A., et al. (2015). "Assessing Cognition in Mice: Assessing Cognition in Mice," in *Current Protocols in Mouse Biology,* eds. J. Auwerx, S.L. Ackerman, S.D. Brown, M.J. Justice & J. Nadeau. (Hoboken, NJ, USA: John Wiley & Sons, Inc.), 331-358.

Ishii, K., Kubo, K.i., Endo, T., Yoshida, K., Benner, S., Ito, Y., et al. (2015). Neuronal Heterotopias Affect the Activities of Distant Brain Areas and Lead to Behavioral Deficits. *Journal of Neuroscience* 35(36)**,** 12432-12445. doi: 10.1523/JNEUROSCI.3648-14.2015.

Lee, K., Kobayashi, Y., Seo, H., Kwak, J.-H., Masuda, A., Lim, C.-S., et al. (2015). Involvement of cAMP-guanine nucleotide exchange factor II in hippocampal long-term depression and behavioral flexibility. *Molecular Brain* 8(1). doi: 10.1186/s13041-015-0130-1.

Netrakanti, P.R., Cooper, B.H., Dere, E., Poggi, G., Winkler, D., Brose, N., et al. (2015). Fast Cerebellar Reflex Circuitry Requires Synaptic Vesicle Priming by Munc13-3. *The Cerebellum* 14(3)**,** 264-283. doi: 10.1007/s12311-015-0645-0.

Ogi, H., Itoh, K., Ikegaya, H., and Fushiki, S. (2015). Alterations of neurotransmitter norepinephrine and gamma-aminobutyric acid correlate with murine behavioral perturbations related to bisphenol A exposure. *Brain and Development* 37(8)**,** 739-746. doi: 10.1016/j.braindev.2014.12.008.

Peltola, M.A., Kuja-Panula, J., Liuhanen, J., Võikar, V., Piepponen, P., Hiekkalinna, T., et al. (2015). AMIGO-Kv2.1 Potassium Channel Complex is Associated With Schizophrenia-Related Phenotypes. *Schizophrenia Bulletin***,** sbv105. doi: 10.1093/schbul/sbv105.

Qiu, H., Li, S., and Le, W. (2015). Impacts of chronic sleep deprivation on learning and memory, autophagy and neuronal apoptosis in mice. *Chinese Journal of Neurology* 48(7)**,** 564-569.

Vázquez, E., Barranco, A., Ramírez, M., Gruart, A., Delgado-García, J.M., Martínez-Lara, E., et al. (2015). Effects of a human milk oligosaccharide, 2′-fucosyllactose, on hippocampal long-term potentiation and learning capabilities in rodents. *The Journal of Nutritional Biochemistry* 26(5)**,** 455-465. doi: 10.1016/j.jnutbio.2014.11.016.

2016

Alboni, S., Poggini, S., Garofalo, S., Milior, G., El Hajj, H., Lecours, C., et al. (2016). Fluoxetine treatment affects the inflammatory response and microglial function according to the quality of the living environment. *Brain, Behavior, and Immunity* 58**,** 261-271. doi: 10.1016/j.bbi.2016.07.155.

Alexandrov, V., Brunner, D., Menalled, L.B., Kudwa, A., Watson-Johnson, J., Mazzella, M., et al. (2016). Large-scale phenome analysis defines a behavioral signature for Huntington's disease genotype in mice. *Nature Biotechnology* 34(8)**,** 838-844. doi: 10.1038/nbt.3587.

Aung, K.H., Kyi-Tha-Thu, C., Sano, K., Nakamura, K., Tanoue, A., Nohara, K., et al. (2016). Prenatal Exposure to Arsenic Impairs Behavioral Flexibility and Cortical Structure in Mice. *Frontiers in Neuroscience* 10. doi: 10.3389/fnins.2016.00137.

Benraiss, A., Wang, S., Herrlinger, S., Li, X., Chandler-Militello, D., Mauceri, J., et al. (2016). Human glia can both induce and rescue aspects of disease phenotype in Huntington disease. *Nature Communications* 7(1). doi: 10.1038/ncomms11758.

Bergamini, G., Cathomas, F., Auer, S., Sigrist, H., Seifritz, E., Patterson, M., et al. (2016). Mouse psychosocial stress reduces motivation and cognitive function in operant reward tests: A model for reward pathology with effects of agomelatine. *European Neuropsychopharmacology* 26(9)**,** 1448-1464. doi: 10.1016/j.euroneuro.2016.06.009.

Heidari, M., Johnstone, D.M., Bassett, B., Graham, R.M., Chua, A.C.G., House, M.J., et al. (2016). Brain iron accumulation affects myelin-related molecular systems implicated in a rare neurogenetic disease family with neuropsychiatric features. *Molecular Psychiatry* 21(11)**,** 1599-1607. doi: 10.1038/mp.2015.192.

Jastrzębska, K., Walczak, M., Cieślak, P.E., Szumiec, Ł., Turbasa, M., Engblom, D., et al. (2016). Loss of NMDA receptors in dopamine neurons leads to the development of affective disorder-like symptoms in mice. *Scientific Reports* 6(1). doi: 10.1038/srep37171.

Jensen, M., Ratner, C., Rudenko, O., Christiansen, S.H., Skov, L.J., Hundahl, C., et al. (2016). Anxiolytic-Like Effects of Increased Ghrelin Receptor Signaling in the Amygdala. *International Journal of Neuropsychopharmacology* 19(5)**,** pyv123. doi: 10.1093/ijnp/pyv123.

Kalm, M., Andreasson, U., Björk-Eriksson, T., Zetterberg, H., Pekny, M., Blennow, K., et al. (2016). C3 deficiency ameliorates the negative effects of irradiation of the young brain on hippocampal development and learning. *Oncotarget* 7(15). doi: 10.18632/oncotarget.8400.

Koss, D.J., Robinson, L., Drever, B.D., Plucińska, K., Stoppelkamp, S., Veselcic, P., et al. (2016). Mutant Tau knock-in mice display frontotemporal dementia relevant behaviour and histopathology. *Neurobiology of Disease* 91**,** 105-123. doi: 10.1016/j.nbd.2016.03.002.

Macpherson, T., Morita, M., Wang, Y., Sasaoka, T., Sawa, A., and Hikida, T. (2016). Nucleus accumbens dopamine D2-receptor expressing neurons control behavioral flexibility in a place discrimination task in the IntelliCage. *Learning & Memory* 23(7)**,** 359-364. doi: 10.1101/lm.042507.116.

Masuda, A., Kobayashi, Y., Kogo, N., Saito, T., Saido, T.C., and Itohara, S. (2016). Cognitive deficits in single App knock-in mouse models. *Neurobiology of Learning and Memory* 135**,** 73-82. doi: 10.1016/j.nlm.2016.07.001.

Milior, G., Lecours, C., Samson, L., Bisht, K., Poggini, S., Pagani, F., et al. (2016). Fractalkine receptor deficiency impairs microglial and neuronal responsiveness to chronic stress. *Brain, Behavior, and Immunity* 55**,** 114-125. doi: 10.1016/j.bbi.2015.07.024.

Ratner, C., Skov, L.J., Raida, Z., Bächler, T., Bellmann-Sickert, K., Le Foll, C., et al. (2016). Effects of Peripheral Neurotensin on Appetite Regulation and Its Role in Gastric Bypass Surgery. *Endocrinology* 157(9)**,** 3482-3492. doi: 10.1210/en.2016-1329.

Sano, K., Isobe, T., Yang, J., Win-Shwe, T.-T., Yoshikane, M., Nakayama, S.F., et al. (2016). In utero and Lactational Exposure to Acetamiprid Induces Abnormalities in Socio-Sexual and Anxiety-Related Behaviors of Male Mice. *Frontiers in Neuroscience* 10. doi: 10.3389/fnins.2016.00228.

Shuaishuai, C., Lei, W., Tianjiao, X., Yin, C., Zhengliang, M., and Xiaoping, G. (2016). Effects of ambient temperature on spatial memory, weight and activity in mice. *International Journal of Anesthesiology and Resuscitation* 37(4)**,** 330-334.

Simmons, D.A., Belichenko, N.P., Ford, E.C., Semaan, S., Monbureau, M., Aiyaswamy, S., et al. (2016). A small molecule p75NTR ligand normalizes signalling and reduces Huntington's disease phenotypes in R6/2 and BACHD mice. *Human Molecular Genetics***,** ddw316. doi: 10.1093/hmg/ddw316.

Too, L.K., Li, K.M., Suarna, C., Maghzal, G.J., Stocker, R., McGregor, I.S., et al. (2016). Deletion of TDO2, IDO-1 and IDO-2 differentially affects mouse behavior and cognitive function. *Behavioural Brain Research* 312**,** 102-117. doi: 10.1016/j.bbr.2016.06.018.

Too, L.K., Li, K.M., Suarna, C., Maghzal, G.J., Stocker, R., McGregor, I.S., et al. (2016). Behavioral and cognitive data in mice with different tryptophan-metabolizing enzymes knocked out. *Data in Brief* 9**,** 275-287. doi: 10.1016/j.dib.2016.08.071.

Too, L.K., McGregor, I.S., Baxter, A.G., and Hunt, N.H. (2016). Altered behaviour and cognitive function following combined deletion of Toll-like receptors 2 and 4 in mice. *Behavioural Brain Research* 303**,** 1-8. doi: 10.1016/j.bbr.2016.01.024.

Too, L.K., Mitchell, A.J., McGregor, I.S., and Hunt, N.H. (2016). Antibody-induced neutrophil depletion prior to the onset of pneumococcal meningitis influences long-term neurological complications in mice. *Brain, Behavior, and Immunity* 56**,** 68-83. doi: 10.1016/j.bbi.2016.01.021.

van Dijk, R.M., Lazic, S.E., Slomianka, L., Wolfer, D.P., and Amrein, I. (2016). Large-scale phenotyping links adult hippocampal neurogenesis to the reaction to novelty: Large scale phenotyping links neurogenesis to novelty. *Hippocampus* 26(5)**,** 646-657. doi: 10.1002/hipo.22548.

Xia, T., Cui, Y., Chu, S., Song, J., Qian, Y., Ma, Z., et al. (2016). Melatonin pretreatment prevents isoflurane-induced cognitive dysfunction by modulating sleep–wake rhythm in mice. *Brain Research* 1634**,** 12-20. doi: 10.1016/j.brainres.2015.10.036.

2017

Cathomas, F., Sigrist, H., Schmid, L., Seifritz, E., Gassmann, M., Bettler, B., et al. (2017). Behavioural endophenotypes in mice lacking the auxiliary GABA B receptor subunit KCTD16. *Behavioural Brain Research* 317**,** 393-400. doi: 10.1016/j.bbr.2016.10.006.

Fischer, M., Cabello, V., Popp, S., Krackow, S., Hommers, L., Deckert, J., et al. (2017). Rsk2 Knockout Affects Emotional Behavior in the IntelliCage. *Behavior Genetics* 47(4)**,** 434-448. doi: 10.1007/s10519-017-9853-3.

Hardt, S., Heidler, J., Albuquerque, B., Valek, L., Altmann, C., Wilken-Schmitz, A., et al. (2017). Loss of synaptic zinc transport in progranulin deficient mice may contribute to progranulin-associated psychopathology and chronic pain. *Biochimica et Biophysica Acta (BBA) - Molecular Basis of Disease* 1863(11)**,** 2727-2745. doi: 10.1016/j.bbadis.2017.07.014.

Hikida, T., Macpherson, T., and Morita, M. (2017). Basal ganglia circuit mechanisms in cognitive learning. *Japanese Journal of Neuropsychopharmacology* 37(2), 35-38.

Holgate, J.Y., Garcia, H., Chatterjee, S., and Bartlett, S.E. (2017). Social and environmental enrichment has different effects on ethanol and sucrose consumption in mice. *Brain and Behavior* 7(8), e00767. doi: 10.1002/brb3.767.

Ismail, N.I.W., Jayabalan, N., Mansor, S.M., Müller, C.P., and Muzaimi, M. (2017). Chronic mitragynine (kratom) enhances punishment resistance in natural reward seeking and impairs place learning in mice: Mitragynine and cognition. *Addiction Biology* 22(4), 967-976. doi: 10.1111/adb.12385.

Lebedeva-Georgievskaya, K.B., Matveeva, M.I., Bazian, A.S., Kudrin, V.S., Narkevich, V.B., Perevezentsev, A.A., et al. (2017). Effect of tail-suspension on the activity, environmental adaptation, exploratory behavior and monoamine turnover in the brain of mice. *Aerospace and Environmental Medicine* 51(1), 39-45. doi: 10.21687/0233-528X-2017-51-1-39-45.

Mijakowska, Z., Łukasiewicz, K., Ziółkowska, M., Lipiński, M., Trąbczyńska, A., Matuszek, Ż., et al. (2017). Autophosphorylation of alpha isoform of calcium/calmodulin-dependent kinase II regulates alcohol addiction-related behaviors: αCaMKII and alcohol addiction. *Addiction Biology* 22(2), 331-341. doi: 10.1111/adb.12327.

Mitjans, M., Begemann, M., Ju, A., Dere, E., Wüstefeld, L., Hofer, S., et al. (2017). Sexual dimorphism of AMBRA1-related autistic features in human and mouse. *Translational Psychiatry* 7(10), e1247. doi: 10.1038/tp.2017.213.

Mohammadi, S.A., Burton, T.J., and Christie, M.J. (2017). α9-nAChR knockout mice exhibit dysregulation of stress responses, affect and reward-related behaviour. *Behavioural Brain Research* 328, 105-114. doi: 10.1016/j.bbr.2017.04.005.

Pelsőczi, P., and Lévay, G. (2017). Effect of Scopolamine on Mice Motor Activity, Lick Behavior and Reversal Learning in the IntelliCage. *Neurochemical Research* 42(12), 3597-3602. doi: 10.1007/s11064-017-2408-4.

Skupio, U., Sikora, M., Korostynski, M., Wawrzczak-Bargiela, A., Piechota, M., Ficek, J., et al. (2017). Behavioral and transcriptional patterns of protracted opioid self-administration in mice: Patterns of opioid addiction. *Addiction Biology* 22(6), 1802-1816. doi: 10.1111/adb.12449.

Stefaniuk, M., Beroun, A., Lebitko, T., Markina, O., Leski, S., Meyza, K., et al. (2017). Matrix Metalloproteinase-9 and Synaptic Plasticity in the Central Amygdala in Control of Alcohol-Seeking Behavior. *Biological Psychiatry* 81(11), 907-917. doi: 10.1016/j.biopsych.2016.12.026.

Wang, L., Wang, F., Zhang, Z., Wu, N., and Sun, T. (2017). Effects of GABAB Receptor Expression in Rat Insular Cortex on the Ability of Spatial Exploration. *Journal of Ningxia Medical University* 39(4).

Wu, N., Wang, F., Jin, Z., Zhang, Z., Wang, L.-K., Zhang, C., et al. (2017). Effects of GABAB receptors in the insula on recognition memory observed with intellicage. *Behavioral and Brain Functions* 13(1). doi: 10.1186/s12993-017-0125-4.

Yang, Y., Chen, X., Min, H., Song, S., Zhang, J., Fan, S., et al. (2017). Persistent mitoKATP Activation Is Involved in the Isoflurane-induced Cytotoxicity. *Molecular Neurobiology* 54(2), 1101-1110. doi: 10.1007/s12035-016-9710-z.

2018

Ajonijebu, D.C., Abboussi, O., Mabandla, M.V., and Daniels, W.M.U. (2018). Differential epigenetic changes in the hippocampus and prefrontal cortex of female mice that had free access to cocaine. *Metabolic Brain Disease* 33(2), 411-420. doi: 10.1007/s11011-017-0116-z.

Akbergenov, R., Duscha, S., Fritz, A.K., Juskeviciene, R., Oishi, N., Schmitt, K., et al. (2018). Mutant MRPS5 affects mitoribosomal accuracy and confers stress-related behavioral alterations. *EMBO reports* 19(11)**,** e46193. doi: 10.15252/embr.201846193.

Atlan, G., Terem, A., Peretz-Rivlin, N., Sehrawat, K., Gonzales, B.J., Pozner, G., et al. (2018). The Claustrum Supports Resilience to Distraction. *Current Biology* 28(17)**,** 2752-2762.e2757. doi: 10.1016/j.cub.2018.06.068.

Beroun, A., Nalberczak-Skóra, M., Harda, Z., Piechota, M., Ziółkowska, M., Cały, A., et al. (2018). Generation of silent synapses in dentate gyrus correlates with development of alcohol addiction. *Neuropsychopharmacology* 43(10)**,** 1989-1999. doi: 10.1038/s41386-018-0119-4.

de Hoz, L., Gierej, D., Lioudyno, V., Jaworski, J., Blazejczyk, M., Cruces-Solís, H., et al. (2018). Blocking c-Fos Expression Reveals the Role of Auditory Cortex Plasticity in Sound Frequency Discrimination Learning. *Cerebral Cortex* 28(5)**,** 1645-1655. doi: 10.1093/cercor/bhx060.

Dere, E., Ronnenberg, A., Tampe, B., Arinrad, S., Schmidt, M., Zeisberg, E., et al. (2018). Cognitive, emotional and social phenotyping of mice in an observer-independent setting. *Neurobiology of Learning and Memory* 150**,** 136-150. doi: 10.1016/j.nlm.2018.02.023.

Dzik, J.M., Puścian, A., Mijakowska, Z., Radwanska, K., and Łęski, S. (2018). PyMICE: A Python library for analysis of IntelliCage data. *Behavior Research Methods* 50(2)**,** 804-815. doi: 10.3758/s13428-017-0907-5.

Endo, N., Ujita, W., Fujiwara, M., Miyauchi, H., Mishima, H., Makino, Y., et al. (2018). Multiple animal positioning system shows that socially-reared mice influence the social proximity of isolation-reared cagemates. *Communications Biology* 1(1)**,** 225. doi: 10.1038/s42003-018-0213-5.

Fuchs, H., Aguilar-Pimentel, J.A., Amarie, O.V., Becker, L., Calzada-Wack, J., Cho, Y.-L., et al. (2018). Understanding gene functions and disease mechanisms: Phenotyping pipelines in the German Mouse Clinic. *Behavioural Brain Research* 352**,** 187-196. doi: 10.1016/j.bbr.2017.09.048.

Harda, Z., Dzik, J.M., Nalberczak-Skóra, M., Meyza, K., Łukasiewicz, K., Łęski, S., et al. (2018). Autophosphorylation of αCaMKII affects social interactions in mice. *Genes, Brain and Behavior* 17(5)**,** e12457. doi: 10.1111/gbb.12457.

Heinla, I., Åhlgren, J., Vasar, E., and Voikar, V. (2018). Behavioural characterization of C57BL/6N and BALB/c female mice in social home cage – Effect of mixed housing in complex environment. *Physiology & Behavior* 188**,** 32-41. doi: 10.1016/j.physbeh.2018.01.024.

Hikida, T. (2018). Homeostatic regulation of basal ganglia circuit for flexible behavior. *Folia Pharmacologica Japonica*.

Kato, T.M., Kubota-Sakashita, M., Fujimori-Tonou, N., Saitow, F., Fuke, S., Masuda, A., et al. (2018). Ant1 mutant mice bridge the mitochondrial and serotonergic dysfunctions in bipolar disorder. *Molecular Psychiatry* 23(10)**,** 2039-2049. doi: 10.1038/s41380-018-0074-9.

Koskela, M., Piepponen, T.P., Andressoo, J.-O., Võikar, V., and Airavaara, M. (2018). Towards developing a model to study alcohol drinking and craving in female mice housed in automated cages. *Behavioural Brain Research* 352**,** 116-124. doi: 10.1016/j.bbr.2018.03.027.

Langfelder, P., Gao, F., Wang, N., Howland, D., Kwak, S., Vogt, T.F., et al. (2018). MicroRNA signatures of endogenous Huntingtin CAG repeat expansion in mice. *PLoS ONE* 13(1)**,** e0190550. doi: 10.1371/journal.pone.0190550.

Maroteaux, G., Arefin, T.M., Harsan, L.A., Darcq, E., Ben Hamida, S., and Kieffer, B.L. (2018). Lack of anticipatory behavior in Gpr88 knockout mice showed by automatized home cage phenotyping. *Genes, Brain and Behavior* 17(8)**,** e12473. doi: 10.1111/gbb.12473.

Marwari, S., and Dawe, G.S. (2018). (R)-fluoxetine enhances cognitive flexibility and hippocampal cell proliferation in mice. *Journal of Psychopharmacology* 32(4)**,** 441-457. doi: 10.1177/0269881118754733.

Masuda, A., Kobayashi, Y., and Itohara, S. (2018). Automated, Long-term Behavioral Assay for Cognitive Functions in Multiple Genetic Models of Alzheimer's Disease, Using IntelliCage. *Journal of Visualized Experiments* (138). doi: 10.3791/58009.

Mätlik, K., Võikar, V., Vilenius, C., Kulesskaya, N., and Andressoo, J.-O. (2018). Two-fold elevation of endogenous GDNF levels in mice improves motor coordination without causing side-effects. *Scientific Reports* 8(1). doi: 10.1038/s41598-018-29988-1.

Oliveros, E., Vázquez, E., Barranco, A., Ramírez, M., Gruart, A., Delgado-García, J., et al. (2018). Sialic Acid and Sialylated Oligosaccharide Supplementation during Lactation Improves Learning and Memory in Rats. *Nutrients* 10(10)**,** 1519. doi: 10.3390/nu10101519.

Orock, A., Logan, S., and Deak, F. (2018). Munc18-1 haploinsufficiency impairs learning and memory by reduced synaptic vesicular release in a model of Ohtahara syndrome. *Molecular and Cellular Neuroscience* 88**,** 33-42. doi: 10.1016/j.mcn.2017.12.002.

Pan, H., Oliveira, B., Saher, G., Dere, E., Tapken, D., Mitjans, M., et al. (2018). Uncoupling the widespread occurrence of anti-NMDAR1 autoantibodies from neuropsychiatric disease in a novel autoimmune model. *Molecular Psychiatry*. doi: 10.1038/s41380-017-0011-3.

Raab, A., Popp, S., Lesch, K.P., Lohse, M.J., Fischer, M., Deckert, J., et al. (2018). Increased fear learning, spatial learning as well as neophobia in Rgs2−/− mice: Increased fear learning spatial learning and neophobiain Rgs2 −/− mice. *Genes, Brain and Behavior* 17(4)**,** e12420. doi: 10.1111/gbb.12420.

Roccaro-Waldmeyer, D.M., Girard, F., Milani, D., Vannoni, E., Prétôt, L., Wolfer, D.P., et al. (2018). Eliminating the VGlut2-Dependent Glutamatergic Transmission of Parvalbumin-Expressing Neurons Leads to Deficits in Locomotion and Vocalization, Decreased Pain Sensitivity, and Increased Dominance. *Frontiers in Behavioral Neuroscience* 12. doi: 10.3389/fnbeh.2018.00146.

Sato, Y., Shinjyo, N., Sato, M., Nilsson, M.K.L., Osato, K., Zhu, C., et al. (2018). Grafting Neural Stem and Progenitor Cells Into the Hippocampus of Juvenile, Irradiated Mice Normalizes Behavior Deficits. *Frontiers in Neurology* 9. doi: 10.3389/fneur.2018.00715.

Ujita, W., Kohyama-Koganeya, A., Endo, N., Saito, T., and Oyama, H. (2018). Mice lacking a functional NMDA receptor exhibit social subordination in a group-housed environment. *The FEBS Journal* 285(1)**,** 188-196. doi: 10.1111/febs.14334.

Voikar, V., Krackow, S., Lipp, H.-P., Rau, A., Colacicco, G., and Wolfer, D.P. (2018). Automated dissection of permanent effects of hippocampal or prefrontal lesions on performance at spatial, working memory and circadian timing tasks of C57BL/6 mice in IntelliCage. *Behavioural Brain Research* 352**,** 8-22. doi: 10.1016/j.bbr.2017.08.048.

Yang, X., Wang, F., Wu, N., Zhao, X., and Sun, T. (2018). Effects of GABAB2 Receptor Expression on the Impairment of Recognition Memory in Lithium Chloride-pilocarpine Epileptic Rat Insular Cortex. *Journal of Ningxia Medical University*.

Zheng, J., Jiang, R., Chen, M., Maimaitiming, Z., Wang, J., Anderson, G.J., et al. (2018). Multi-Copper Ferroxidase–Deficient Mice Have Increased Brain Iron Concentrations and Learning and Memory Deficits. *The Journal of Nutrition* 148(4)**,** 643-649. doi: 10.1093/jn/nxy012.

2019

Ajonijebu, D.C., Abboussi, O., Mabandla, M.V., and Daniels, W.M.U. (2019). Cocaine-induced inheritable epigenetic marks may be altered by changing early postnatal fostering. *NeuroReport* 30(17)**,** 1157-1165. doi: 10.1097/WNR.0000000000001332.

Cały, A., Śliwińska, M.A., Ziółkowska, M., Łukasiewicz, K., Pagano, R., Nowacka, A., et al. (2019). Contribution of PSD-95 protein to reward location memory. *bioRxiv*. doi: 10.1101/590109.

Chen, C., Krueger-Burg, D., and de Hoz, L. (2019). Wide sensory filters underlie performance in memory-based discrimination and generalization. *PLoS ONE* 14(4)**,** e0214817. doi: 10.1371/journal.pone.0214817.

Chwin, N., Kiryk, A., Bijoch, L., Hamed, A., and Konopka, W. (2019). Up-regulation of PI3K-Akt-mTOR signaling pathway in neurons affects cognitive functions and social interactions in a mouse model. *Acta Neurobiologiae Experimentalis* 79.

Festa, B.P., Berquez, M., Gassama, A., Amrein, I., Ismail, H.M., Samardzija, M., et al. (2019). OCRL deficiency impairs endolysosomal function in a humanized mouse model for Lowe syndrome and Dent disease. *Human Molecular Genetics* 28(12)**,** e1005058. doi: 10.1093/hmg/ddy449.

Fröhlich, H., Kollmeyer, M.L., Linz, V.C., Stuhlinger, M., Groneberg, D., Reigl, A., et al. (2019). Gastrointestinal dysfunction in autism displayed by altered motility and achalasia *Foxp1* mice. *Proceedings of the National Academy of Sciences* 116(44)**,** 22237-22245. doi: 10.1073/pnas.1911429116.

Geraghty, A.C., Gibson, E.M., Ghanem, R.A., Greene, J.J., Ocampo, A., Goldstein, A.K., et al. (2019). Loss of Adaptive Myelination Contributes to Methotrexate Chemotherapy-Related Cognitive Impairment. *Neuron* 103(2)**,** 250-265.e258. doi: 10.1016/j.neuron.2019.04.032.

Hardt, S., Fischer, C., Vogel, A., Wilken-Schmitz, A., and Tegeder, I. (2019). Distal infraorbital nerve injury: a model for persistent facial pain in mice. *PAIN* 160(6)**,** 1431-1447. doi: 10.1097/j.pain.0000000000001518.

Jensen, L.R., Garrett, L., Hölter, S.M., Rathkolb, B., Rácz, I., Adler, T., et al. (2019). A mouse model for intellectual disability caused by mutations in the X-linked 2′-O-methyltransferase Ftsj1 gene. *Biochimica et Biophysica Acta (BBA) - Molecular Basis of Disease* 1865(9)**,** 2083-2093. doi: 10.1016/j.bbadis.2018.12.011.

Marwari, S., and Dawe, G.S. (2019). Effects of haloperidol on cognitive function and behavioural flexibility in the IntelliCage social home cage environment. *Behavioural Brain Research* 371**,** 111976. doi: 10.1016/j.bbr.2019.111976.

Poggini, S., Golia, M.T., Alboni, S., Milior, G., Sciarria, L.P., Viglione, A., et al. (2019). Combined Fluoxetine and Metformin Treatment Potentiates Antidepressant Efficacy Increasing IGF2 Expression in the Dorsal Hippocampus. *Neural Plasticity* 2019**,** 1-12. doi: 10.1155/2019/4651031.

Rudenko, O., Springer, C., Skov, L.J., Madsen, A.N., Hasholt, L., Nørremølle, A., et al. (2019). Ghrelin-mediated improvements in the metabolic phenotype in the R6/2 mouse model of Huntington's disease. *Journal of Neuroendocrinology* 31(7). doi: 10.1111/jne.12699.

Ruud, J., Alber, J., Tokarska, A., Engström Ruud, L., Nolte, H., Biglari, N., et al. (2019). The Fat Mass and Obesity-Associated Protein (FTO) Regulates Locomotor Responses to Novelty via D2R Medium Spiny Neurons. *Cell Reports* 27(11)**,** 3182-3198.e3189. doi: 10.1016/j.celrep.2019.05.037.

Too, L.K., Yau, B., Baxter, A.G., McGregor, I.S., and Hunt, N.H. (2019). Double deficiency of toll-like receptors 2 and 4 alters long-term neurological sequelae in mice cured of pneumococcal meningitis. *Scientific Reports* 9(1)**,** 16189. doi: 10.1038/s41598-019-52212-7.

van Dijk, R.M., Wiget, F., Wolfer, D.P., Slomianka, L., and Amrein, I. (2019). Consistent within-group covariance of septal and temporal hippocampal neurogenesis with behavioral phenotypes for exploration and memory retention across wild and laboratory small rodents. *Behavioural Brain Research***,** 11.

Welz, P.-S., Zinna, V.M., Symeonidi, A., Koronowski, K.B., Kinouchi, K., Smith, J.G., et al. (2019). BMAL1-Driven Tissue Clocks Respond Independently to Light to Maintain Homeostasis. *Cell* 177(6)**,** 1436-1447.e1412. doi: 10.1016/j.cell.2019.05.009.

Wilhelmsson, U., Kalm, M., Pekna, M., and Pekny, M. (2019). Nestin Null Mice Show Improved Reversal Place Learning. *Neurochemical Research*. doi: 10.1007/s11064-019-02854-w.

Wilhelmsson, U., Pozo-Rodrigalvarez, A., Kalm, M., de Pablo, Y., Widestrand, Å., Pekna, M., et al. (2019). The role of GFAP and vimentin in learning and memory. *Biological Chemistry***,** 10.

2020

Chotard, É., Mohammadi, F., Julien, P., Berthiaume, L., Rudkowska, I., and Bertrand, N. (2020). Drinkable lecithin nanovesicles to study the biological effects of individual hydrophobic macronutrients and food preferences. *Food Chemistry* 322**,** 126736. doi: 10.1016/j.foodchem.2020.126736.

Fischer, C., Endle, H., Schumann, L., Wilken-Schmitz, A., Kaiser, J., Gerber, S., et al. (2020). Prevention of age-associated neuronal hyperexcitability with improved learning and attention upon knockout or antagonism of LPAR2. *Cellular and Molecular Life Sciences*. doi: 10.1007/s00018-020-03553-4.

Garrett, L., Chang, Y.J., Niedermeier, K.M., Heermann, T., Enard, W., Fuchs, H., et al. (2020). A truncating Aspm allele leads to a complex cognitive phenotype and region-specific reductions in parvalbuminergic neurons. *Translational Psychiatry* 10(1)**,** 66. doi: 10.1038/s41398-020-0686-0.

Harda, Z., Spyrka, J., Jastrzębska, K., Szumiec, Ł., Bryksa, A., Klimczak, M., et al. (2020). Loss of mu and delta opioid receptors on neurons expressing dopamine receptor D1 has no effect on reward sensitivity. *Neuropharmacology* 180**,** 108307. doi: 10.1016/j.neuropharm.2020.108307.

Horigane, S.i., Ozawa, Y., Zhang, J., Todoroki, H., Miao, P., Haijima, A., et al. (2020). A mouse model of Timothy syndrome exhibits altered social competitive dominance and inhibitory neuron development. *FEBS Open Bio* 10(8)**,** 1436-1446. doi: 10.1002/2211-5463.12924.

Hühne, A., Volkmann, P., Stephan, M., Rossner, M., and Landgraf, D. (2020). An in-depth neurobehavioral characterization shows anxiety-like traits, impaired habituation behavior, and restlessness in male <i>Cryptochrome</i> -deficient mice. *Genes, Brain and Behavior* 19(8). doi: 10.1111/gbb.12661.

Kimura, E., Suzuki, G., Uramaru, N., Endo, T., and Maekawa, F. (2020). Behavioral impairments in infant and adult mouse offspring exposed to 2,3,7,8-tetrabromodibenzofuran in utero and via lactation. *Environment International* 142**,** 105833. doi: 10.1016/j.envint.2020.105833.

Kiryk, A., Janusz, A., Zglinicki, B., Turkes, E., Knapska, E., Konopka, W., Lipp, H.-P. and Kaczmarek, L. (2020). IntelliCage as a tool for measuring mouse behavior – 20 years perspective. *Behavioural Brain Research* 388**,** 112620. doi: 10.1016/j.bbr.2020.112620.

Konarzewski, M., Goncerzewicz, A., Knapska, E., Dzik, J., and rkiewicz, T.G. (2020). Energetic Costs of Cognitive Abilities: Testing the Expensive Tissue Hypothesis. *Authorea*. doi: 10.22541/au.159069206.66900218.

Lebitko, T., Dzik, J., Jędrzejewska-Szmek, J., Chaturvedi, M., Jaworski, T., Nikolaev, T., et al. (2020). c-Fos-MMP-9 pathway in central amygdala mediates approach motivation but not reward consumption. *bioRxiv*. doi: 10.1101/2020.04.17.044792.

Liu, S., Jin, Z., Zhang, Y., Rong, S., He, W., Sun, K., et al. (2020). The Glucagon-Like Peptide-1 Analogue Liraglutide Reduces Seizures Susceptibility, Cognition Dysfunction and Neuronal Apoptosis in a Mouse Model of Dravet Syndrome. *Front Pharmacol* 11**,** 136. doi: 10.3389/fphar.2020.00136.

Liu, Y., Burton, T., Rayner, B.S., San Gabriel, P.T., Shi, H., El Kazzi, M., et al. (2020). The role of sodium thiocyanate supplementation during dextran sodium sulphate-stimulated experimental colitis. *Archives of Biochemistry and Biophysics* 692**,** 108490. doi: 10.1016/j.abb.2020.108490.

Mateusz, S.L., Andrzej, L.M., Ewa, L., Joanna, U.-C., Ludwika, K., Puscian, A., et al. (2020). β-catenin signaling via astrocyte-encoded TCF7L2 regulates neuronal excitability and social behavior. *bioRxiv*. doi: 10.1101/2020.11.28.402099.

Mehr, A., Hick, M., Ludewig, S., Müller, M., Herrmann, U., von Engelhardt, J., et al. (2020). Lack of APP and APLP2 in GABAergic Forebrain Neurons Impairs Synaptic Plasticity and Cognition. *Cerebral Cortex* 30(7)**,** 4044-4063. doi: 10.1093/cercor/bhaa025.

Mingrone, A., Kaffman, A., and Kaffman, A. (2020). The Promise of Automated Home-Cage Monitoring in Improving Translational Utility of Psychiatric Research in Rodents. *Frontiers in Neuroscience* 14**,** 618593. doi: 10.3389/fnins.2020.618593.

Morello, F., Voikar, V., Parkkinen, P., Panhelainen, A., Rosenholm, M., Makkonen, A., et al. (2020). ADHD-like behaviors caused by inactivation of a transcription factor controlling the balance of inhibitory and excitatory neuron development in the mouse anterior brainstem. *Translational Psychiatry* 10(1)**,** 357. doi: 10.1038/s41398-020-01033-8.

Nieraad, H., de Bruin, N., Arne, O., Hofmann, M.C.J., Schmidt, M., Saito, T., et al. (2020). Impact of Hyperhomocysteinemia and Different Dietary Interventions on Cognitive Performance in a Knock-in Mouse Model for Alzheimer's Disease. *Nutrients* 12(11)**,** 3248. doi: 10.3390/nu12113248.

Oizumi, H., Miyazaki, S., Tabuchi, M., Endo, T., Omiya, Y., and Mizoguchi, K. (2020). Kamikihito Enhances Cognitive Functions and Reward-Related Behaviors of Aged C57BL/6J Mice in an Automated Behavioral Assay System. *Frontiers in Pharmacology* 11**,** 1037. doi: 10.3389/fphar.2020.01037.

Pelsőczi, P., Kelemen, K., Csölle, C., Nagy, G., Lendvai, B., Román, V., et al. (2020). Disrupted Social Hierarchy in Prenatally Valproate-Exposed Autistic-Like Rats. *Frontiers in Behavioral Neuroscience* 13**,** 295. doi: 10.3389/fnbeh.2019.00295.

Puścian, A., Winiarski, M., Łęski, S., Charzewski, Ł., Nikolaev, T., Borowska, J., et al. (2020). Chronic fluoxetine treatment impairs motivation and reward learning by affecting neuronal plasticity in the central amygdala. *British Journal of Pharmacology***,** bph.15319. doi: 10.1111/bph.15319.

Richter, S.H. (2020). Automated Home-Cage Testing as a Tool to Improve Reproducibility of Behavioral Research? *Frontiers in Neuroscience* 14**,** 383. doi: 10.3389/fnins.2020.00383.

Serchov, T., Schwarz, I., Theiss, A., Sun, L., Holz, A., Döbrössy, M.D., et al. (2020). Enhanced adenosine A1 receptor and Homer1a expression in hippocampus modulates the resilience to stress-induced depression-like behavior. *Neuropharmacology* 162**,** 107834. doi: 10.1016/j.neuropharm.2019.107834.

Serykh, A., Khrapova, M.V., Dubrovina, N.I., Petrova, E.S., Mikhnevich, N., Starostina, M.V., et al. (2020). The increased density of the habenular neurons, high impulsivity, aggression and resistant fear memory in Disc1-Q31L genetic mouse model of depression. *Behavioural Brain Research* 392**,** 112693. doi: 10.1016/j.bbr.2020.112693.

Śliwińska, M.A., Cały, A., Borczyk, M., Ziółkowska, M., Skonieczna, E., Chilimoniuk, M., et al. (2020). Long-term Memory Upscales Volume of Postsynaptic Densities in the Process that Requires Autophosphorylation of αCaMKII. *Cerebral Cortex* 30(4)**,** 2573-2585. doi: 10.1093/cercor/bhz261.

Vogel, A., Wilken-Schmitz, A., Hummel, R., Lang, M., Gurke, R., Schreiber, Y., et al. (2020). Low brain endocannabinoids associated with persistent non-goal directed nighttime hyperactivity after traumatic brain injury in mice. *Scientific Reports* 10(1)**,** 14929. doi: 10.1038/s41598-020-71879-x.

Voikar, V., and Gaburro, S. (2020). Three Pillars of Automated Home-Cage Phenotyping of Mice: Novel Findings, Refinement, and Reproducibility Based on Literature and Experience. *Frontiers in Behavioral Neuroscience* 14**,** 575434. doi: 10.3389/fnbeh.2020.575434.

Zhang, C., Liu, Q., Yu, C.-Y., Wang, F., Shao, Y., Sun, K.-S., et al. (2020). G Protein-Coupled Estrogen Receptor 1 Knockout Deteriorates MK-801-Induced Learning and Memory Impairment in Mice. *Frontiers in Behavioral Neuroscience* 14**,** 157. doi: 10.3389/fnbeh.2020.00157.

Zheng, Y., Wu, M., Gao, T., Meng, L., Ding, X., Meng, Y., et al. (2020). GPER-Deficient Rats Exhibit Lower Serum Corticosterone Level and Increased Anxiety-Like Behavior. *Neural Plasticity* 2020**,** 1-22. doi: 10.1155/2020/8866187.

2021

Arinrad, S., Wilke, J.B.H., Seelbach, A., Doeren, J., Hindermann, M., Butt, U.J., et al. (2021). NMDAR1 autoantibodies amplify behavioral phenotypes of genetic white matter inflammation: a mild encephalitis model with neuropsychiatric relevance. *Molecular Psychiatry*. doi: 10.1038/s41380-021-01392-8.

Bahader, I. (2021). *Behavioral and electrophysiological assessment of hearing function in mice with deficient sound encoding at inner hair cell ribbon synapse.* MD, Georg-August-University.

Balan, S., Iwayama, Y., Ohnishi, T., Fukuda, M., Shirai, A., Yamada, A., et al. (2021). A loss-of-function variant in SUV39H2 identified in autism-spectrum disorder causes altered H3K9 trimethylation and dysregulation of protocadherin β-cluster genes in the developing brain. *Molecular Psychiatry*. doi: 10.1038/s41380-021-01199-7.

Baumann, P., Schriever, S.C., Kullmann, S., Zimprich, A., Peter, A., Gailus-Durner, V., et al. (2021). Diabetes type 2 risk gene Dusp8 is associated with altered sucrose reward behavior in mice and humans. *Brain Behav* 11(1)**,** e01928. doi: 10.1002/brb3.1928.

Cały, A., Śliwińska, M.A., Ziółkowska, M., Łukasiewicz, K., Pagano, R., Dzik, J.M., et al. (2021). PSD-95 in CA1 Area Regulates Spatial Choice Depending on Age. *The Journal of Neuroscience* 41(11)**,** 2329-2343. doi: 10.1523/JNEUROSCI.1996-20.2020.

Cao, G., Wei, X., Li, W., Yin, H., Lang, W., Wei, P., et al. (2021). Verification of a multi-function closed maze for the detection of affective disorder and spatial cognitive impairment in post-weaning socially isolated rats. *Neuroscience Letters* 763**,** 136192. doi: 10.1016/j.neulet.2021.136192.

Cisbani, G., Poggini, S., Laflamme, N., Pons, V., Tremblay, M.-È., Branchi, I., et al. (2021). The Intellicage system provides a reproducible and standardized method to assess behavioral changes in cuprizone-induced demyelination mouse model. *Behavioural Brain Research* 400**,** 113039. doi: 10.1016/j.bbr.2020.113039.

Grieco, F., Bernstein, B.J., Biemans, B., Bikovski, L., Burnett, C.J., Cushman, J.D., et al. (2021). Measuring Behavior in the Home Cage: Study Design, Applications, Challenges, and Perspectives. *Frontiers in Behavioral Neuroscience* 15**,** 735387. doi: 10.3389/fnbeh.2021.735387.

Iman, I.N., Ahmad, N.A.Z., Mohd Yusof, N.A., Talib, U.N., Norazit, A., Kumar, J., et al. (2021). Mitragynine (Kratom)-Induced Cognitive Impairments in Mice Resemble Delta9-THC and Morphine Effects: Reversal by Cannabinoid CB(1) Receptor Antagonism. *Front Pharmacol* 12**,** 708055. doi: 10.3389/fphar.2021.708055.

Iman, I.N., Yusof, N.A.M., Talib, U.N., Ahmad, N.A.Z., Norazit, A., Kumar, J., et al. (2021). The IntelliCage System: A Review of Its Utility as a Novel Behavioral Platform for a Rodent Model of Substance Use Disorder. *Frontiers in Behavioral Neuroscience* 15**,** 683780. doi: 10.3389/fnbeh.2021.683780.

Jablonska, J., Szumiec, L., Zielinski, P., and Parkitna, J.R. (2021). Time elapsed between choices in a probabilistic task correlates with repeating the same decision. *European Journal of Neuroscience* 53(8), 2639-2654. doi: 10.1111/ejn.15144.

Kahnau, P., Guenther, A., Boon, M.N., Terzenbach, J.D., Hanitzsch, E., Lewejohann, L., et al. (2021). Lifetime Observation of Cognition and Physiological Parameters in Male Mice. *Frontiers in Behavioral Neuroscience* 15, 709775. doi: 10.3389/fnbeh.2021.709775.

Kandadai, K.S., Kotur, M.B., Dokalis, N., Amrein, I., Keller, C.W., Munz, C., et al. (2021). ATG5 in microglia does not contribute vitally to autoimmune neuroinflammation in mice. *Autophagy* 17(11), 3566-3576. doi: 10.1080/15548627.2021.1883880.

Koskela, M., Piepponen, T.P., Andressoo, J.-O., Võikar, V., and Airavaara, M. (2021). Female C57BL/6J Mice Show Alcohol-Seeking Behaviour after Withdrawal from Prolonged Alcohol Consumption in the Social Environment. *Alcohol and Alcoholism*, agab032. doi: 10.1093/alcalc/agab032.

Koskela, M., Piepponen, T.P., Lindahl, M., Harvey, B.K., Andressoo, J.-O., Võikar, V., et al. (2021). The overexpression of GDNF in nucleus accumbens suppresses alcohol-seeking behavior in group-housed C57Bl/6J female mice. *Journal of Biomedical Science* 28(1), 87. doi: 10.1186/s12929-021-00782-y.

Kraft, V., Schmitz, K., Wilken-Schmitz, A., Geisslinger, G., Sisignano, M., and Tegeder, I. (2021). Trehalose Reduces Nerve Injury Induced Nociception in Mice but Negatively Affects Alertness. *Nutrients* 13(9), 2953. doi: 10.3390/nu13092953.

Lopez-Caperuchipi, S., Kürzinger, L., Hopp-Krämer, S., Albert-Weißenberger, C., Paul, M.M., Sirén, A.-L., et al. (2021). Posttraumatic learning deficits correlate with initial trauma severity and chronic cellular reactions after closed head injury in male mice. *Experimental Neurology* 341, 113721. doi: 10.1016/j.expneurol.2021.113721.

Markova, E.V., and Knyazheva, M.A. (2021). IMMUNE CELLS AS A POTENTIAL THERAPEUTIC AGENT IN THE TREATMENT OF DEPRESSION. *Medical Immunology (Russia)* 23(4), 699-704. doi: 10.15789/1563-0625-ICA-2277.

Meng, H.-R., Suenaga, T., Edamura, M., Fukuda, A., Ishida, Y., Nakahara, D., et al. (2021). Functional MHCI deficiency induces ADHD-like symptoms with increased dopamine D1 receptor expression. *Brain, Behavior, and Immunity* 97, 22-31. doi: 10.1016/j.bbi.2021.05.015.

Mifflin, M.A., Winslow, W., Surendra, L., Tallino, S., Vural, A., and Velazquez, R. (2021). Sex differences in the IntelliCage and the Morris water maze in the APP/PS1 mouse model of amyloidosis. *Neurobiology of Aging* 101, 130-140. doi: 10.1016/j.neurobiolaging.2021.01.018.

Nakamura, T., Nakajima, K., Kobayashi, Y., Itohara, S., Kasahara, T., Tsuboi, T., et al. (2021). Functional and behavioral effects of <i>de novo</i> mutations in calcium-related genes in patients with bipolar disorder. *Human Molecular Genetics* 30(19), 1851-1862. doi: 10.1093/hmg/ddab152.

Picard, K., Bisht, K., Poggini, S., Garofalo, S., Golia, M.T., Basilico, B., et al. (2021). Microglial-glucocorticoid receptor depletion alters the response of hippocampal microglia and neurons in a chronic unpredictable mild stress paradigm in female mice. *Brain, Behavior, and Immunity* 97, 423-439. doi: 10.1016/j.bbi.2021.07.022.

Poggini, S., Matte Bon, G., Golia, M.T., Ciano Albanese, N., Viglione, A., Poleggi, A., et al. (2021). Selecting antidepressants according to a drug-by-environment interaction: A comparison of fluoxetine and minocycline effects in mice living either in enriched or stressful conditions. *Behavioural Brain Research* 408, 113256. doi: 10.1016/j.bbr.2021.113256.

Rankovic, V., Vogl, C., Dörje, N.M., Bahader, I., Duque-Afonso, C.J., Thirumalai, A., et al. (2021). Overloaded Adeno-Associated Virus as a Novel Gene Therapeutic Tool for Otoferlin-Related Deafness. *Frontiers in Molecular Neuroscience* 13, 600051. doi: 10.3389/fnmol.2020.600051.

Rivero, O., Alhama-Riba, J., Ku, H.-P., Fischer, M., Ortega, G., Álmos, P., et al. (2021). Haploinsufficiency of the Attention-Deficit/Hyperactivity Disorder Risk Gene St3gal3 in Mice Causes Alterations in Cognition and Expression of Genes Involved in Myelination and Sialylation. *Frontiers in Genetics* 12, 688488. doi: 10.3389/fgene.2021.688488.

Ruffini, N., Müller, M.B., Schmitt, U., and Gerber, S. (2021). IntelliPy: a GUI for analyzing IntelliCage data. *Bioinformatics* 37(21), 3972-3973. doi: 10.1093/bioinformatics/btab682.

Schroeder, S., Hofer, S.J., Zimmermann, A., Pechlaner, R., Dammbrueck, C., Pendl, T., et al. (2021). Dietary spermidine improves cognitive function. *Cell Reports* 35(2), 108985. doi: 10.1016/j.celrep.2021.108985.

Schumann, L., Wilken-Schmitz, A., Trautmann, S., Vogel, A., Schreiber, Y., Hahnefeld, L., et al. (2021). Increased Fat Taste Preference in Progranulin-Deficient Mice. *Nutrients* 13(11), 4125. doi: 10.3390/nu13114125.

Simmons, K.E., Healey, K.L., Li, Q., Moore, S.D., and Klein, R.C. (2021). Effects of sex and genotype in human <i>APOE</i> -targeted replacement mice on alcohol self-administration measured with the automated IntelliCage system before and after repeated mild traumatic brain injury. *Alcoholism: Clinical and Experimental Research* 45(11), 2231-2245. doi: 10.1111/acer.14717.

Simmons, K.E., White, M.A., Healey, K.L., and Klein, R.C. (2021). Comparison of Enriched Housing Conditions on Automated Activity Monitoring and Behavior Following Repeated Mild Traumatic Brain Injury in Female C57BL/6 Mice. *SSRN Electronic Journal*. doi: 10.2139/ssrn.3899303.

Srimat Kandadai, K., Kotur, M.B., Dokalis, N., Amrein, I., Keller, C.W., Münz, C., et al. (2021). ATG5 in microglia does not contribute vitally to autoimmune neuroinflammation in mice. *Autophagy* 17(11), 3566-3576. doi: 10.1080/15548627.2021.1883880.

Stefaniuk, M., Pawłowska, M., Nowicka, K., Barański, M., Zielinski, Z., Bijoch, Ł., et al. (2021). A whole-brain imaging-based systems approach to understand origin of addiction in binge-like drinking model. *bioRxiv*. doi: 10.1101/2021.02.17.431586.

Stetter, C., Lopez-Caperuchipi, S., Hopp-Krämer, S., Bieber, M., Kleinschnitz, C., Sirén, A.-L., et al. (2021). Amelioration of Cognitive and Behavioral Deficits after Traumatic Brain Injury in Coagulation Factor XII Deficient Mice. *International Journal of Molecular Sciences* 22(9), 4855. doi: 10.3390/ijms22094855.

Sun, L., Verkaik-Schakel, R.-N., Biber, K., Plösch, T., and Serchov, T. (2021). Antidepressant treatment is associated with epigenetic alterations of Homer1 promoter in a mouse model of chronic depression. *Journal of Affective Disorders* 279, 501-509. doi: 10.1016/j.jad.2020.10.040.

Tikhonova, M.A., Amstislavskaya, T.G., Ho, Y.-J., Akopyan, A.A., Tenditnik, M.V., Ovsyukova, M.V., et al. (2021). Neuroprotective Effects of Ceftriaxone Involve the Reduction of Aβ Burden and Neuroinflammatory Response in a Mouse Model of Alzheimer's Disease. *Frontiers in Neuroscience* 15, 736786. doi: 10.3389/fnins.2021.736786.

Tran, B.N., Valek, L., Wilken-Schmitz, A., Fuhrmann, D.C., Namgaladze, D., Wittig, I., et al. (2021). Reduced exploratory behavior in neuronal nucleoredoxin knockout mice. *Redox Biology* 45, 102054. doi: 10.1016/j.redox.2021.102054.

Volkmann, P., Stephan, M., Krackow, S., Jensen, N., and Rossner, M.J. (2021). PsyCoP – A Platform for Systematic Semi-Automated Behavioral and Cognitive Profiling Reveals Gene and

Environment Dependent Impairments of Tcf4 Transgenic Mice Subjected to Social Defeat. *Frontiers in Behavioral Neuroscience* 14**,** 618180. doi: 10.3389/fnbeh.2020.618180.

Wen, Y., Ding, X., Guan, Q., Hu, W., Wang, B., Hu, Q., et al. (2021). Effects of exposure to urban particulate matter SRM 1648a during pregnancy on the neurobehavioral development of offspring mice. *Ecotoxicology and Environmental Safety* 215**,** 112142. doi: 10.1016/j.ecoenv.2021.112142.

Wilke, J.B.H., Hindermann, M., Berghoff, S.A., Zihsler, S., Arinrad, S., Ronnenberg, A., et al. (2021). Autoantibodies against NMDA receptor 1 modify rather than cause encephalitis. *Molecular Psychiatry*. doi: 10.1038/s41380-021-01238-3.

Winslow, W., McDonough, I., Tallino, S., Decker, A., Vural, A.S., and Velazquez, R. (2021). IntelliCage Automated Behavioral Phenotyping Reveals Behavior Deficits in the 3xTg-AD Mouse Model of Alzheimer's Disease Associated With Brain Weight. *Frontiers in Aging Neuroscience* 13**,** 720214. doi: 10.3389/fnagi.2021.720214.

Xing, L., Kubik-Zahorodna, A., Namba, T., Pinson, A., Florio, M., Prochazka, J., et al. (2021). Expression of human-specific ARHGAP11B in mice leads to neocortex expansion and increased memory flexibility. *The EMBO Journal* 40(13). doi: 10.15252/embj.2020107093.

Yang, Y., Sun, K., Liu, W., Li, X., Tian, W., Shuai, P., et al. (2021). The phosphatidylserine flippase β-subunit Tmem30a is essential for normal insulin maturation and secretion. *Molecular Therapy* 29(9)**,** 2854-2872. doi: 10.1016/j.ymthe.2021.04.026.

Yang, Y., Wang, H., Song, S., Liu, Y., Zhang, W., Zhang, J., et al. (2021). STAT3 Involved in Cellular Vulnerability to Isoflurane. *Research Square*.

2022

Barranco, A., Garcia, L., Gruart, A., Delgado-Garcia, J.M., Rueda, R., and Ramirez, M. (2022). Effects of β-Hydroxy β-Methylbutyrate Supplementation on Working Memory and Hippocampal Long-Term Potentiation in Rodents. *Nutrients* 14(5)**,** 1090. doi: 10.3390/nu14051090.

Ben-Simon, Y., Kaefer, K., Velicky, P., Csicsvari, J., Danzl, J.G., and Jonas, P. (2022). A direct excitatory projection from entorhinal layer 6b neurons to the hippocampus contributes to spatial coding and memory. *Nature Communications* 13(1)**,** 4826. doi: 10.1038/s41467-022-32559-8.

Coulibaly, A.P. (2022). Neutrophil modulation of behavior and cognition in health and disease: The unexplored role of an innate immune cell. *Immunological Reviews* 311(1)**,** 177-186. doi: 10.1111/imr.13123.

Esmaeili, A., Antonova, A., Sitnikova, E., and Smirnov, K. (2022). Whisker trimming during infanthood modifies the development of spike-wave discharges and behavioral sequences in IntelliCage impulsivity paradigm in adult WAG/Rij rats. *Behavioural Brain Research* 418**,** 113627. doi: 10.1016/j.bbr.2021.113627.

Goncerzewicz, A., Górkiewicz, T., Dzik, J.M., Jędrzejewska-Szmek, J., Knapska, E., and Konarzewski, M. (2022). Brain size, gut size and cognitive abilities: the energy trade-offs tested in artificial selection experiment. *Proceedings of the Royal Society B: Biological Sciences* 289(1972)**,** 20212747. doi: 10.1098/rspb.2021.2747.

Hahnefeld, L., Vogel, A., Gurke, R., Geisslinger, G., Schäfer, M.K.E., and Tegeder, I. (2022). Phosphatidylethanolamine Deficiency and Triglyceride Overload in Perilesional Cortex Contribute to Non-Goal-Directed Hyperactivity after Traumatic Brain Injury in Mice. *Biomedicines* 10(4)**,** 914. doi: 10.3390/biomedicines10040914.

Hühne, A., Echtler, L., Kling, C., Stephan, M., Schmidt, M.V., Rossner, M.J., et al. (2022). Circadian gene × environment perturbations influence alcohol drinking in *Cryptochrome*-deficient mice. *Addiction Biology* 27(1). doi: 10.1111/adb.13105.

Juskeviciene, R., Fritz, A.-K., Brilkova, M., Akbergenov, R., Schmitt, K., Rehrauer, H., et al. (2022). Phenotype of Mrps5-Associated Phylogenetic Polymorphisms Is Intimately Linked to Mitoribosomal Misreading. *International Journal of Molecular Sciences* 23(8)**,** 4384. doi: 10.3390/ijms23084384.

Kahnau, P., Jaap, A., Diederich, K., Gygax, L., Rudeck, J., and Lewejohann, L. (2022). Determining the value of preferred goods based on consumer demand in a home-cage based test for mice. *Behavior Research Methods*. doi: 10.3758/s13428-022-01813-8.

Kempermann, G., Lopes, J.B., Zocher, S., Schilling, S., Ehret, F., Garthe, A., et al. (2022). The individuality paradigm: Automated longitudinal activity tracking of large cohorts of genetically identical mice in an enriched environment. *Neurobiology of Disease* 175**,** 105916. doi: 10.1016/j.nbd.2022.105916.

Klein, C.J.M.I., Budiman, T., Homberg, J.R., Verma, D., Keijer, J., and van Schothorst, E.M. (2022). Measuring Locomotor Activity and Behavioral Aspects of Rodents Living in the Home-Cage. *Frontiers in Behavioral Neuroscience* 16. doi: 10.3389/fnbeh.2022.877323.

Leite-Almeida, H., Castelhano-Carlos, M.J., and Sousa, N. (2022). New Horizons for Phenotyping Behavior in Rodents: The Example of Depressive-Like Behavior. *Frontiers in Behavioral Neuroscience* 15**,** 811987. doi: 10.3389/fnbeh.2021.811987.

Lipp, H.-P., and Wolfer, D.P. (2022). Behavior is movement only but how to interpret it? Problems and pitfalls in translational neuroscience—a 40-year experience. *Frontiers in Behavioral Neuroscience* 16**,** 958067. doi: 10.3389/fnbeh.2022.958067.

Lopez-Caperuchipi, S. (2022). *Charakterisierung zellulärer Veränderungen und kognitiver Verhaltensweisen in einem Model vom Schädel-Hirn Trauma in männlichen Mäusen.* MD, Julius-Maximilians-Universität Würzburg,.

Markova, E.V., Knyazheva, M.A., Tikhonova, M.A., and Amstislavskaya, T.G. (2022). Structural and functional characteristics of the hippocampus in depressive-like recipients after transplantation of in vitro caffeine-modulated immune cells. *Neuroscience Letters* 786**,** 136790. doi: 10.1016/j.neulet.2022.136790.

Mätlik, K., Garton, D.R., Montaño-Rodríguez, A.R., Olfat, S., Eren, F., Casserly, L., et al. (2022). Elevated endogenous GDNF induces altered dopamine signalling in mice and correlates with clinical severity in schizophrenia. *Molecular Psychiatry* 27(8)**,** 3247-3261. doi: 10.1038/s41380-022-01554-2.

Morozova, M.V., Borisova, M.A., Snytnikova, O.A., Achasova, K.M., Litvinova, E.A., Tsentalovich, Y.P., et al. (2022). Colitis-associated intestinal microbiota regulates brain glycine and host behavior in mice. *bioRxiv*. doi: 10.1101/2022.03.07.483210.

Pagano, R., Salamian, A., Zielinski, J., Beroun, A., Nalberczak-Skóra, M., Skonieczna, E., et al. (2022). Arc controls alcohol cue relapse by a central amygdala mechanism. *Molecular Psychiatry*. doi: 10.1038/s41380-022-01849-4.

Pham, H., Yin, T., and D'Adamio, L. (2022). Initial assessment of the spatial learning, reversal, and sequencing task capabilities of knock- in rats with humanizing mutations in the Aβ-coding region of *App*. *bioRxiv*. doi: 10.1101/2022.01.24.477482.

Puścian, A., and Knapska, E. (2022). Blueprints for measuring natural behavior. *iScience* 25(7)**,** 104635. doi: 10.1016/j.isci.2022.104635.

Puścian, A., Winiarski, M., Borowska, J., Łęski, S., Górkiewicz, T., Chaturvedi, M., et al. (2022). Targeted therapy of cognitive deficits in fragile X syndrome. *Molecular Psychiatry*. doi: 10.1038/s41380-022-01527-5.

Schacke, S., Kirkpatrick, J., Stocksdale, A., Bauer, R., Hagel, C., Riecken, L.B., et al. (2022). Ezrin deficiency triggers glial fibrillary acidic protein upregulation and a distinct reactive astrocyte phenotype. *Glia* 70(12)**,** 2309-2329. doi: 10.1002/glia.24253.

Shishelova, A.Y., Smirnov, K., and Raevskiĭ, V.V. (2022). Influence of early social isolation on general activity and spatial learning in adult WAG/Rij rats. *Developmental Psychobiology* 64(7). doi: 10.1002/dev.22319.

Stephan, M., Schoeller, J., Raabe, F.J., Schmitt, A., Hasan, A., Falkai, P., et al. (2022). Spironolactone alleviates schizophrenia-related reversal learning in Tcf4 transgenic mice subjected to social defeat. *Schizophrenia* 8(1)**,** 77. doi: 10.1038/s41537-022-00290-4.

Syding, L.A. (2022). *Mouse models for Angelman syndrome: generation and characterization.* PhD, Charles University Prague.

Syding, L.A., Kubik-Zahorodna, A., Nickl, P., Novosadova, V., Kopkanova, J., Kasparek, P., et al. (2022). Generation and Characterization of a Novel Angelman Syndrome Mouse Model with a Full Deletion of the Ube3a Gene. *Cells* 11(18)**,** 2815. doi: 10.3390/cells11182815.

Tegeder, I., Vogel, A., Ueberbach, T., Wilken-Schmitz, A., Jungenitz, T., Schmid, T., et al. (2022). Optogenetic early life pain leads to cortical hyperexcitability, nociceptive hypersensitivity and repetitive behavior. *Research Square*.

Vasić, V., Barth, K., Bicker, F., Schumann, U., Maurer, C., Heinig, N., et al. (2022). Less is more - loss of EGFL7 improves memory by upregulation of VEGF-D. *bioRxiv*. doi: 10.1101/2022.04.07.487327.

Winiarski, M., Kondrakiewicz, L., Kondrakiewicz, K., Jędrzejewska-Szmek, J., Turzyński, K., Knapska, E., et al. (2022). Social deficits in BTBR T+ Itpr3tf/J mice vary with ecological validity of the test. *Genes, Brain and Behavior* 21(5). doi: 10.1111/gbb.12814.

Xiao, L., Jiang, S., Wang, Y., Gao, C., Liu, C., Huo, X., et al. (2022). Continuous high-frequency deep brain stimulation of the anterior insula modulates autism-like behavior in a valproic acid-induced rat model. *Journal of Translational Medicine* 20(1)**,** 570. doi: 10.1186/s12967-022-03787-9.

Yang, Y., Liu, Y., Zhu, J., Song, S., Huang, Y., Zhang, W., et al. (2022). Neuroinflammation-mediated mitochondrial dysregulation involved in postoperative cognitive dysfunction. *Free Radical Biology and Medicine* 178**,** 134-146. doi: 10.1016/j.freeradbiomed.2021.12.004.

Yesiltepe, M., Yin, T., Tambini, M.D., Breuillaud, L., Zehntner, S.P., and D'Adamio, L. (2022). Late-long-term potentiation magnitude, but not Aβ levels and amyloid pathology, is associated with behavioral performance in a rat knock-in model of Alzheimer disease. *Frontiers in Aging Neuroscience* 14**,** 1040576. doi: 10.3389/fnagi.2022.1040576.

2023 (January-October 15 2023)

Barth, K., Vasic, V., McDonald, B., Heinig, N., Wagner, M.C., Schumann, U., et al. (2023). EGFL7 loss correlates with increased VEGF-D expression, upregulating hippocampal adult neurogenesis and improving spatial learning and memory. *Cell Mol Life Sci* 80(2)**,** 54. doi: 10.1007/s00018-023-04685-z.

Caly, A., Ziolkowska, M., Pagano, R., Salamian, A., Sliwinska, M.A., Sotoudeh, N., et al. (2023). Autophosphorylation of alphaCaMKII regulates alcohol consumption by controlling sedative effects of alcohol and alcohol-induced loss of excitatory synapses. *Addict Biol* 28(5)**,** e13276. doi: 10.1111/adb.13276.

Chen, and de Hoz, L. (2023). The perceptual categorization of multidimensional stimuli is hierarchically organized. *iScience* 26**,** 18. doi: https://doi.org/10.1016/j.isci.2023.106941

Dzirkale, Z., Pilipenko, V., Pijet, B., Klimaviciusa, L., Upite, J., Protokowicz, K., Kaczmarek, L., and Jansone, B. (2023). Long-term behavioural alterations in mice following transient cerebral ischemia. Behav. Brain Res. 452, 114589. doi: 10.1016/j.bbr.2023.114589.

Frycz, B.A., Nowicka, K., Konopka, A., Hoener, M.C., Bulska, E., Kaczmarek, L., et al. (2023). Activation of trace amine-associated receptor 1 (TAAR1) transiently reduces alcohol drinking in socially housed mice. *Addict Biol* 28(7)**,** e13285. doi: 10.1111/adb.13285.

Gundersen, B.B., O'Brien, T.O., Schaffler, M.D., Schultz, M.N., Tsukahara, T., Martin Lorenzo, S., et al. (2023). Towards Preclinical Validation of Arbaclofen (R-baclofen) Treatment for 16p11.2 Deletion Syndrome. *bioRxiv***,** 2023.2005.2001.538987. doi: 10.1101/2023.05.01.538987.

Hühne-Landgraf, A., Laurent, K., Frisch, M.K., Wehr, M.C., Rossner, M.J., and Landgraf, D. (2023). Rescue of Comorbid Behavioral and Metabolic Phenotypes of Arrhythmic Mice by Restoring Circadian Cryptochrome1/2 Expression in the Suprachiasmatic Nucleus. *Biological Psychiatry Global Open Science*. doi: https://doi.org/10.1016/j.bpsgos.2023.06.002.

Ikuta, K., Joho, D., Kakeyama, M., and Matsumoto, M. (2023). Bifidobacterium animalis subsp. lactis and arginine mixture intake improves cognitive flexibility in mice. *Frontiers in Nutrition* 10. doi: 10.3389/fnut.2023.1164809.

Jetsonen, E., Didio, G., Winkel, F., Llach Pou, M., Boj, C., Kuczynski-Noyau, L., et al. (2023). Activation of TrkB in Parvalbumin interneurons is required for the promotion of reversal learning in spatial and fear memory by antidepressants. *Neuropsychopharmacology* 48(7)**,** 1021-1030. doi: 10.1038/s41386-023-01562-y.

Jörimann, M., Maliković, J., Wolfer, D.P., Pryce, C.R., Endo, T., Benner, S., et al. (2023). Bank Voles Show More Impulsivity in IntelliCage Learning Tasks than Wood Mice. *Neuroscience* 510**,** 157-170. doi: 10.1016/j.neuroscience.2022.11.011.

Kahnau, P., Jaap, A., Urmersbach, B., Diederich, K., and Lewejohann, L. (2023). Development of an IntelliCage based Cognitive Bias Test for Mice. *Open Research Europe 2022* 2:128. doi: https://doi.org/10.12688/openreseurope.15294.1.

Kahnau, P., Mieske, P., Wilzopolski, J., Kalliokoski, O., Mandillo, S., Hölter, S.M., et al. (2023). "Development and Application of Home Cage Monitoring in Laboratory Mice and Rats: a Systematic Review". bioRxiv).

Li, L., Wang, Q., Sun, X., Li, Z., Liu, S., Zhang, X., et al. (2023). Activation of RhoA pathway participated in the changes of emotion, cognitive function and hippocampal synaptic plasticity in juvenile chronic stress rats. *International Journal of Biological Macromolecules* 233**,** 123652. doi: https://doi.org/10.1016/j.ijbiomac.2023.123652.

Li, X., Gao, Y., Han, X., Tang, S., Li, N., Liu, X., et al. (2023). Maresin1 ameliorates postoperative cognitive dysfunction in aged rats by potentially regulating the NF-kappaB pathway to inhibit astrocyte activation. *Exp Gerontol* 176**,** 112168. doi: 10.1016/j.exger.2023.112168.

Mohammadi, F., Bertrand, N., and Rudkowska, I. (2023). C57bl/6 Mice Show Equivalent Taste Preferences toward Ruminant and Industrial Trans Fatty Acids. *Nutrients* 15(3). doi: 10.3390/nu15030610.

Nagaeva, E., Schäfer, A., Linden, A.-M., Elsilä, L.V., Ryazantseva, M., Umemori, J., et al. (2023). Somatostatin-expressing neurons in the ventral tegmental area innervate specific forebrain regions and are involved in the stress response. *bioRxiv*.

Nalberczak-Skóra, M., Beroun, A., Skonieczna, E., Cały, A., Ziółkowska, M., Pagano, R., et al. (2023). Impaired synaptic transmission in dorsal dentate gyrus increases impulsive alcohol seeking. *Neuropsychopharmacology* 48(3)**,** 436-447. doi: 10.1038/s41386-022-01464-5.

Niiranen, L., Stenback, V., Tulppo, M., Herzig, K.H., and Makela, K. (2023). Interplay between Learning and Voluntary Wheel Running in Male C57BL/6NCrl Mice. *International Journal of Molecular Sciences* 24(5). doi: ARTN 4259, 10.3390/ijms24054259.

Ojanen, S., Kuznetsova, T., Kharybina, Z., Voikar, V., Lauri, S.E., and Taira, T. (2023). Interneuronal GluK1 kainate receptors control maturation of GABAergic transmission and network synchrony in the hippocampus. *Mol Brain* 16(1)**,** 43. doi: 10.1186/s13041-023-01035-9.

Perschler, L. (2023). *Die Effekte von Mitragynin auf den Alkoholkonsum beim sozialen Trinken.* MD, Friedrich-Alexander-Universität Erlangen-Nürnberg.

Perschler, L. (2023). *Die Effekte von Mitragynin auf den Alkoholkonsum beim sozialen Trinken : Eine präklinische Studie im Mausmodell.*

Plum, T., Binzberger, R., Thiele, R., Shang, F., Postrach, D., Fung, C., et al. (2023). Mast cells link immune sensing to antigen-avoidance behaviour. *Nature*. doi: 10.1038/s41586-023-06188-0.

Poggini, S., Lopez, M.B., Albanese, N.C., Golia, M.T., Ibáñez, F.G., Limatola, C., et al. (2023). Minocycline treatment improves cognitive and functional plasticity in a preclinical mouse model of major depressive disorder. *Behavioural Brain Research***,** 114295. doi: 10.1016/j.bbr.2023.114295.

Pupikina, M., and Sitnikova, E. (2023). Sex Differences in Behavior and Learning Abilities in Adult Rats. *Life (Basel)* 13(2). doi: 10.3390/life13020547.

Radlicka, A., Jabłońska, J., Lenarczyk, M., Szumiec, Ł., Harda, Z., Bagińska, M., et al. (2023). Nonmotor symptoms associated with progressive loss of dopaminergic neurons in a mouse model of Parkinson's disease. *BioRxiv*. doi: 10.1101/2023.01.23.525182.

Radwańska K, Pagano R, Salamian A, Skonieczna E, Wojtas B, Gielniewski B, Harda Z, Cały A, Havekes R, Abel T. (2023). Molecular fingerprints in the hippocampus of alcohol seeking during withdrawal. Res. Sq. 2023, rs.3.rs-3337670. doi: 10.21203/rs.3.rs-3337670/v1.

Sasaki, T., Saito, H., Furukawa, Y., Tominaga, T., Kitajima, S., Kanno, J., et al. (2023). Exposure to bisphenol A or its phenolic analogs during early life induces different types of anxiety-like behaviors after maturity in male mice. *The Journal of Toxicological Sciences* 48(4)**,** 211-219. doi: 10.2131/jts.48.211.

Shemesh, Y., and Chen, A. (2023). A paradigm shift in translational psychiatry through rodent neuroethology. *Mol Psychiatry* 28(3)**,** 993-1003. doi: 10.1038/s41380-022-01913-z.

Stefaniuk, M., Pawlowska, M., Baranski, M., Nowicka, K., Zielinski, Z., Bijoch, L., et al. (2023). Global brain c-Fos profiling reveals major functional brain networks rearrangements after alcohol reexposure. *Neurobiol Dis* 178**,** 106006. doi: 10.1016/j.nbd.2023.106006.

Vogel, A., Ueberbach, T., Wilken-Schmitz, A., Hahnefeld, L., Franck, L., Weyer, M.-P., et al. (2023). Repetitive and compulsive behavior after Early-Life-Pain associated with reduced long-chain sphingolipid species.

Wu, N., Sun, T., Wu, X., Chen, H., and Zhang, Z. (2023). Modulation of GABAB receptors in the insula bidirectionally affects associative memory of epilectic rats in both spatial and non-spatial operant tasks. *Frontiers in Behavioral Neuroscience* 16**,** 1042227. doi: 10.3389/fnbeh.2022.1042227.

Yamamoto, H., Lee-Okada, H.C., Ikeda, M., Nakamura, T., Saito, T., Takata, A., et al. (2023). GWAS-identified bipolar disorder risk allele in the FADS1/2 gene region links mood episodes and unsaturated fatty acid metabolism in mutant mice. *Mol Psychiatry*. doi: 10.1038/s41380-023-01988-2.

Zhu, S., Shi, J., Jin, Q., Zhang, Y., Zhang, R., Chen, X., Wang, C., Shi, T., and Li, L. (2023). Mitochondrial dysfunction following repeated administration of alprazolam causes attenuation of hippocampus-dependent memory consolidation in mice. Aging 15. doi: 10.18632/aging.205087.
